# In Silico Evaluation of Antifungal Compounds from Marine Sponges against COVID-19-Associated Mucormycosis

**DOI:** 10.3390/md20030215

**Published:** 2022-03-20

**Authors:** Omkar Pokharkar, Hariharan Lakshmanan, Grigory Zyryanov, Mikhail Tsurkan

**Affiliations:** 1 Department of Organic & Bio-Molecular Chemistry, Chemical Engineering Institute, Ural Federal University, Mira St. 19, 620002 Yekaterinburg, Russia; omkarpokharkar93@gmail.com; 2 La Trobe Institute of Molecular Science, Plenty Rd & Kingsbury Dr., Bundoora, Melbourne, VIC 3086, Australia; harrygocam@icloud.com; 3 Postovsky Institute of Organic Synthesis of RAS (Ural Division), 22/20, S. Kovalevskoy/Akademicheskaya St., 620990 Yekaterinburg, Russia; 4 Leibniz Institute of Polymer Research, 01005 Dresden, Germany

**Keywords:** disseminated mucormycosis, COVID-19, SARS-CoV-2, CAM, bioactive compounds, molecular docking, marine drugs, antifungal, antiviral, marine sponges

## Abstract

The world is already facing the devastating effects of the SARS-CoV-2 pandemic. A disseminated mucormycosis epidemic emerged to worsen this situation, causing havoc, especially in India. This research aimed to perform a multitargeted docking study of marine-sponge-origin bioactive compounds against mucormycosis. Information on proven drug targets and marine sponge compounds was obtained via a literature search. A total of seven different targets were selected. Thirty-five compounds were chosen using the PASS online program. For homology modeling and molecular docking, FASTA sequences and 3D structures for protein targets were retrieved from NCBI and PDB databases. Autodock Vina in PyRx 0.8 was used for docking studies. Further, molecular dynamics simulations were performed using the IMODS server for top-ranked docked complexes. Moreover, the drug-like properties and toxicity analyses were performed using Lipinski parameters in Swiss-ADME, OSIRIS, ProTox-II, pkCSM, and StopTox servers. The results indicated that naamine D, latrunculin A and S, (+)-curcudiol, (+)-curcuphenol, aurantoside I, and hyrtimomine A had the highest binding affinity values of −8.8, −8.6, −9.8, −11.4, −8.0, −11.4, and −9.0 kcal/mol, respectively. In sum, all MNPs included in this study are good candidates against mucormycosis. (+)-curcudiol and (+)-curcuphenol are promising compounds due to their broad-spectrum target inhibition potential.

## 1. Introduction

Mucormycosis, also called zygomycosis and black fungus, is a rare, non-contagious fungal infection that can cause high morbidity and mortality. The mucormycetes are a notorious group of fungi belonging to the Mucorales order and Mucoromycotina subphylum [1]. These molds can commonly be found in the soil, on plant surfaces, and in decaying fruits, vegetables, bread, and other compost piles. A majority of mold species are harmless as they are incapable of tolerating human body temperature. Unfortunately, a thermotolerant type was recently identified in COVID-19 patients worldwide [2], especially in India. An individual can be infected by inhaling or through burns and open wounds on the skin coming into close contact with the spores [3]. Upon infection, the host’s blood vessels are blocked as the spores produce hyphae, which lead to swelling and eventual death of the surrounding tissues. This infection can affect the brain, lungs, skin, and sinuses [4,5], with the severity of the outcome depending on the immune system’s efficacy and certain underlying medical conditions. Certain medical conditions, such as diabetes mellitus, various types of cancers, organ or stem cell transplants, neutropenia, hemochromatosis, metabolic acidosis, and viral diseases such as HIV, and recently, SARS-CoV-2 [6], are known to enhance the risk of mucormycosis infection.

The mortality rate of mucormycosis before COVID-19 was around 54% [7], but disseminated mucormycosis caused 96% fatality [8]. The combination of SARS-CoV-2 and disseminated mucormycosis is, therefore, the worst-case scenario for any individual [9]. This mold infection is considered to be extremely opportunistic, causing severe damage to the host. India is the country most impacted by this sudden epidemic. Over 47,000 cases were reported in India by July 2021. Out of which, approximately 28,000 were active cases [10,11]. For the period of August 2021 to February 2022, the exact number of COVID-19-associated mucormycosis cases in India remains uncertain. However, besides India, at least 17 other countries reported CAM cases. These countries were the United States of America, Mexico, France, Germany, Austria, the Netherlands, United Kingdom, Czech Republic, Italy, Kuwait, Iran, Turkey, Lebanon, Brazil, Chile, China, Pakistan, and Bangladesh [12].

In particular, there is an uptrend in orbital mucormycosis cases post COVID-19 infection. The COVID-19-associated mucormycosis (CAM) primarily affects the sinus and the bony cavities holding the eyeballs. There is a connection between black fungus infection and the usage of steroids to treat COVID-19 patients. The steroids are used to reduce inflammation in the lungs of the patients but also cause blood sugar levels to rise abnormally, making an individual prone to mucormycosis infection [13]. CAM was found to be prevalent in the male population (78.9%). Cases that emerged due to diabetes mellitus and corticosteroid were reported to be 80% and 76.3%, respectively. Around 30.7% of CAM patients died [14]. Current medical treatment options include amphotericin B, isavuconazole, posaconazole, and invasive treatment for surgical removal of infected tissues [15]. These antifungal drugs and surgeries have serious side effects and limited efficacy. Therefore, safe alternatives to treat mucormycosis are urgently needed.

Many studies are being conducted around the globe testing natural bioactive compounds to treat SARS-CoV-2 and mucormycosis. Bioactive compounds from marine organisms hold the potential to inhibit different viruses and fungi [16,17,18,19,20,21,22]. The purpose of this in silico study was to investigate the antifungal potential of bioactive compounds originating from marine sponges. Our research included six different classes of compounds such as alkaloids (naamines, naamidines [22,23,24,25,26], hyrtimomines [27,28], and topsentins [29,30,31,32]), macrolides (latrunculins [33,34,35,36]), bioactive metabolites (xestodecalactones [37,38,39]), sesquiterpene phenols [(+)-curcudiol, (+)-curcuphenol] [40,41,42,43], hydroxpyran-2-ones (tetillapyrone and nortetillapyrone [44,45,46,47,48]), and tetratomic acid glycosides (aurantosides [49,50,51,52,53]). These compounds were probed for seven therapeutic targets belonging to *Rhizopus delemar*, *Candida albicans*, *Rhizopus arrhizus*, *Rhizopus chinensis*, and finally, *Rhizopus microsporus var. chinensis*. These targets were CotH3, mucoricin, lanosterol 14 alpha-demethylase, exo-1,3-beta-glucan synthase, RdRp, fungal lipase, and rhizopuspepsin, respectively. Three currently used drugs, amphotericin B, isavuconazole, and posaconazole were included to compare with selected antifungal candidates. This research pinpointed the suitable fungal targets and highlighted specific MNPs based on their potential targeted biological activity. Ultimately, the drug-likeness properties, as well as the potentially harmful and toxic properties of the MNPs above, were evaluated and elucidated in this work. 

## 2. Results

### 2.1. Drug Targets Selection

Table 1 summarizes relevant information about the targets, such as target name, NCBI and PDB accession IDs, subcellular localization probability, and finally, the role of each protein in contributing to the wellbeing of the fungus.

RNA-dependent RNA polymerase, part of a novel LTR retro transposon of Rhizopus is abbreviated as RdRp. It is responsible for synthesizing RNA from a template RNA. RdRp is a well-studied target for viruses and was recently recognized as a potential fungal target. Even though the RVT_1 region of RdRp is extracellularly situated, it can utilize cytoplasmic triphosphates such as ATP, GTP, UTP, and CTP for RNA replication. CotH3 spore coat protein in a fungus is considered a hallmark of pathogenicity; it is also present in Mucorales, the fungi causing mucormycosis. Therefore, this protein coating offers protection to fungi and is an important drug target. CotH region is situated outside the cell. A fungal CYP450-dependent enzyme called lanosterol 14 alpha-demethylase converts lanosterol to ergosterol. This enzyme is necessary for the fungi’s survival as ergosterol maintains the integrity of the fungal plasma membrane. This target is located on the plasma membrane. Mucoricin is a ricin-like toxin responsible for the fungus pathogenicity. This toxin enhances the permeability of the blood vessel walls, leading to host cell necrosis and apoptosis. This protein is situated in the cytoplasm of the cell. Certain disease-causing features of fungi are dependent on the enzymes such as lipase and rhizopuspepsin. These proteins impart virulence to the fungi [54,55]. Thus, they can be considered promising target candidates. Both fungal lipase and rhizopuspepsin are situated in the extracellular region. Ultimately, exo-1,3-beta-glucan synthase is potentially an important target as it contributes directly to the construction of the cell wall by synthesizing beta-glucan, an abundant polysaccharide present in the fungal cell wall. 

### 2.2. Energy Minimization

Mentioned below is the outcome for the YASARA and UCSF Chimera energy minimization of the modeled structures. In Figure S1, both energy-minimized and non-energy-minimized 3D structures obtained from YASARA are visualized. There is no visual output from UCSF Chimera software.

In YASARA, for CotH3 modeled structure, initial energy was about −147,415.0 KJ/mol. After minimization, the energy was about −153,062.8 KJ/mol. In the case of mucoricin, the initial energy was −98,072.7 KJ/mol. Post minimization process, the energy decreased to −99,161.0 KJ/mol. For RdRp, before minimization, the energy was −125,912.5 KJ/mol, and after minimization, the energy was lowered to −133,822.3 KJ/mol. Finally, the energy of lanosterol 14 alpha-demethylase structure before minimization was −267,875.3 KJ/mol and after minimization, it was −275,767.5 KJ/mol.

In UCSF Chimera, for CotH3 protein, the initial energy was approximately −8168.08 KJ/mol. After minimization, the energy was lowered to −15,377.7 KJ/mol. The energy of mucoricin initially was −5530.4 KJ/mol and post minimization the energy obtained was −9353.01 KJ/mol. In the case of RdRp, initially the energy was −15,475.7 KJ/mol. After minimization, the final energy was –22,214.7 KJ/mol. Finally, for the structure of lanosterol 14 alpha-demethylase, the energy was −33,485.2 KJ/mol, which after minimization changed to −46,402.8 KJ/mol. 

### 2.3. Structure Analysis and Quality Estimation

**Figure 1:**
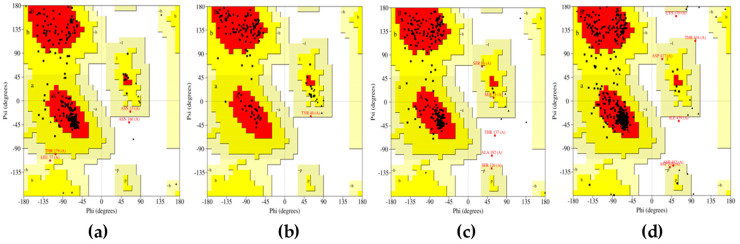
Ramachandran plots for energy-minimized proteins from YASARA: (**a**) CotH3; (**b**) mucoricin; (**c**) RdRp; (**d**) lanosterol 14 alpha-demethylase.

Structure analysis outcomes of energy-minimized modeled structures from YASARA were obtained. The CotH region from CotH3 showed that 88.1% of residues were present in the allowed region (Figure 1). In the additional allowed region, there were 10.1% of residues present. About 8.3% of residues were present in the generally permitted region. Only 0.4% of the residues were in the non-favorable region. In the case of mucoricin, the obtained structure had 85.6% of its residues in the favorable region. In the additional region, around 13.6% were in the additional allowed region. Only 0.8% of residues were in the generally permitted region. There were no residues present in the disallowed region of the plot. The modeled RVT_1 region structure of RdRp indicated that 89.7% of the residues were in the favored area. The additional allowed region consisted of 8% of the residues, and generally allowed regions had 1.3% of residues. Only 0.9% of residues were present in the disallowed region. Lanosterol 14 alpha-demethylase modeled structure indicated that 89% of the residues were located in the favorable region. In the additional allowed region, 9.7% of the residues were present. In the generally allowed area, only 0.9% of residues were present. The remaining 0.4% of the residues were found in the unfavorable region. Thus, all the modeled 3D protein structures were of good quality, as the maximum amount of residues were in favored regions and were a minority in the non-favored areas.

**Figure 2:**
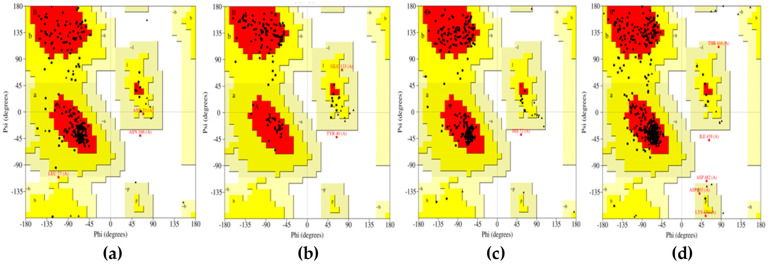
Ramachandran plots for energy-minimized proteins from UCSF Chimera: (**a**) CotH3; (**b**) mucoricin; (**c**) RdRp; (**d**) lanosterol 14 alpha-demethylase.

In the case of energy-minimized structures from UCSF Chimera (Figure 2), the outcomes were slightly different. The CotH region protein structure showed 84.6% of the amino acids in the favorable region. In the additional allowed region, 14.1% of residues were present. In the generally allowed region, only 0.9% of residues were present. Only 0.4% of amino acid residues were located in the non-favorable region. The mucoricin structure showed 85.6% residues in the favorable region. Approximately 12.9% of residues were found to be present in the additional allowed region. The generally allowed and non-favorable regions consisted of 0.8% of amino acids each. Further, the structure of RVT_1 region of RdRp displayed 89.7% residues in the desired region. About 9.8% of residues were present in the additional allowed region. However, the generally allowed region had no residues present. Only 0.4% of residues were located in the unfavorable region. Finally, the lanosterol 14 alpha-demethylase structure presented 89.4% amino acids in the favorable region. In total, 9.4% of residues were present in the generally allowed region. About 0.7% residues were present in the generally allowed region, and 0.4% residues were found in the non-favorable region.

All in all, the Ramachandran plot outcome for mucoricin and RdRp was similar to YASARA. However, in the case of CotH3, the results indicated lower quality. On the contrary, the output for lanosterol 14 alpha-demethylase was slightly better as compared to YASARA.

**Figure 3:**
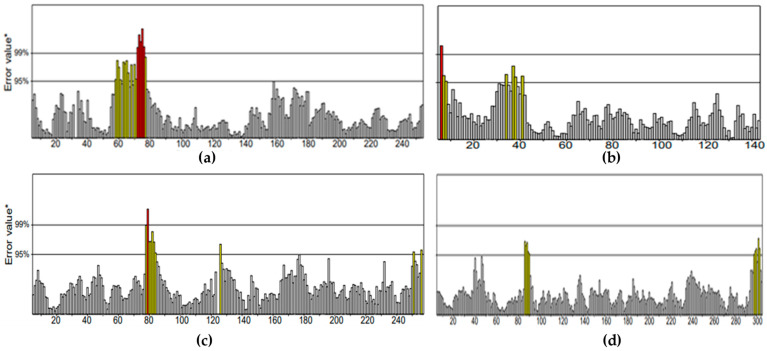
ERRAT plots for energy-minimized proteins from YASARA: (**a**) CotH3; (**b**) mucoricin; (**c**) RdRp; (**d**) lanosterol 14 alpha-demethylase. (* The Y-axis is the error axis with two lines which indicates the confidence with the possibility to reject regions that exceed error value).

The ERRAT plot (Figure 3) estimated the quality factor for energy-minimized protein structures obtained from YASARA. CotH3, mucoricin, RdRp, and lanosterol 14 alpha-demethylase were 93.145%, 94.928%, 95.984%, and 96.061%, respectively. It can be concluded that all the structures were of good resolution. Comparatively, RdRp and lanosterol 14-alpha demethylase were the best. 

**Figure 4:**
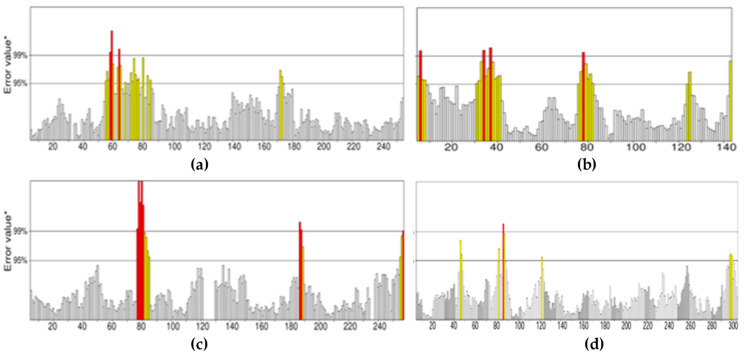
ERRAT plots for energy-minimized proteins from UCSF Chimera: (**a**) CotH3; (**b**) mucoricin; (**c**) RdRp; (**d**) lanosterol 14 alpha-demethylase. (* The Y-axis is the error axis with two lines which indicates the confidence with the possibility to reject regions that exceed error value).

Figure 4 shows that in the case of energy-minimized structures from UCSF Chimera, the quality factor for CotH3, mucoricin, RdRp, and lanosterol 14 alpha-demethylase was 91.532%, 82.014%, 93.802%, and 97.012%, respectively. The structures for CotH3, mucoricin, and RdRp cannot be considered to be high-resolution structures. However, lanosterol 14 alpha-demethylase structure from Chimera was found to be of the highest resolution as compared to YASARA. On the other hand, the structures of CotH3, mucoricin, and RdRp from YASARA were found to be of high resolution as compared to Chimera. In sum, all high-quality structures were selected and used in the study further. It was discovered that performing energy minimization before protein structure and quality analysis improved the outcome of Ramachandran and ERRAT plots. 

**Figure 5:**
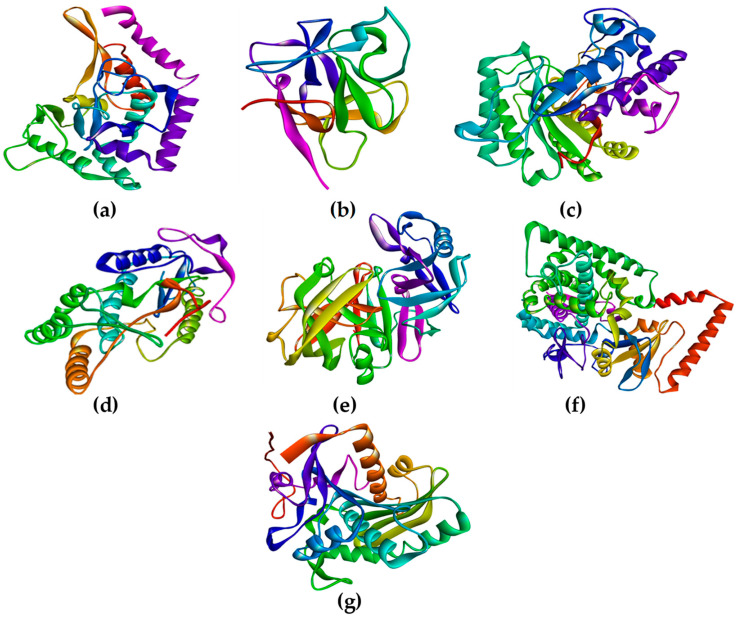
Three-dimensional protein target structures: (**a**) CotH3; (**b**) mucoricin; (**c**) exo-1,3-beta-glucan synthase (**d**) RdRp; (**e**) rhizopuspepsin; (**f**) lanosterol 14 alpha-demethylase; (**g**) fungal lipase.

Upon visualization of structures in Chimera and Discovery Studio Visualizer, it was found that all structures consisted of one single chain; chain A (Figure 5). The figure below represents the finalized structures that were further used for CAST-p analysis, followed by the molecular docking procedure. 

### 2.4. CAST-p Active Site Prediction

**Figure 6:**
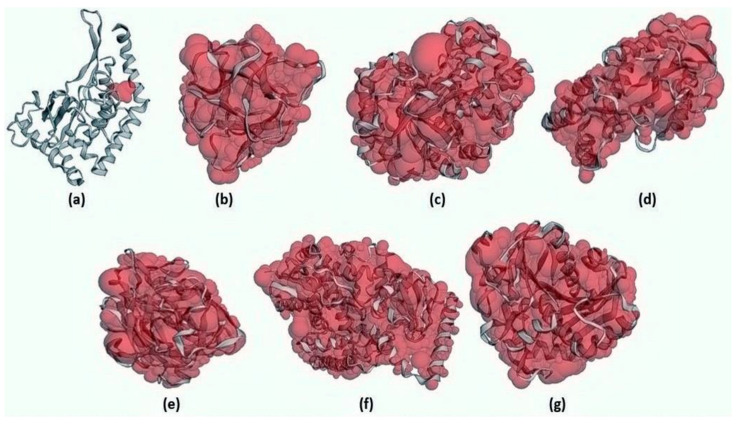
CAST-p pocket estimation: (**a**) CotH3 (**b**) mucoricin (**c**) exo-1,3-beta-glucan synthase (**d**) RdRp (**e**) rhizopuspepsin (**f**) lanosterol 14 alpha-demethylase (**g**) fungal lipase.

The surface areas (SA) for the targets CotH3, mucoricin, exo-1,3-beta-glucan synthase, RdRp, rhizopuspepsin, lanosterol 14 alpha-demethylase, and lipase were estimated to be 79.476 Å, 8922.343 Å, 26,926.662 Å, 13,919.246 Å, 19,017.162 Å, 33,721.828 Å, and 18,104.027 Å, respectively. Figure 6 depicts these numbers in the form of high-resolution 3D output. 

### 2.5. Ligands Screening and Selection Criteria

The candidate MNPs were found via a literature search. There are hundreds of marine-sponge-based antifungal compounds. In order to limit the length of this study, seven categories of sponge MNPs belonging to different classes were considered. The focus was to explore naamines, naamidines, hyrtimomines, xestodecalactones, topsentins, latrunculins, and the aurantosides in detail. The database search output showed name A-G, naamidine A-J, hyrtimomine A-K, xestodecalactone A-F, topsentin A, C, D, latrunculin A, B, C, D, M, S, T, and finally, aurantosides A-K. A total of 60 MNPs were discovered. There were two conditions to filter the suitable MNPs.

1. Must be discoverable in databases, such as PubChem and ChemSpider.
2. Must pass the PASS online screening (accuracy > 80%)—show inhibition potential against the chosen targets. The value for the probability of being active must be higher than the value for being inactive.

**Figure 7:**
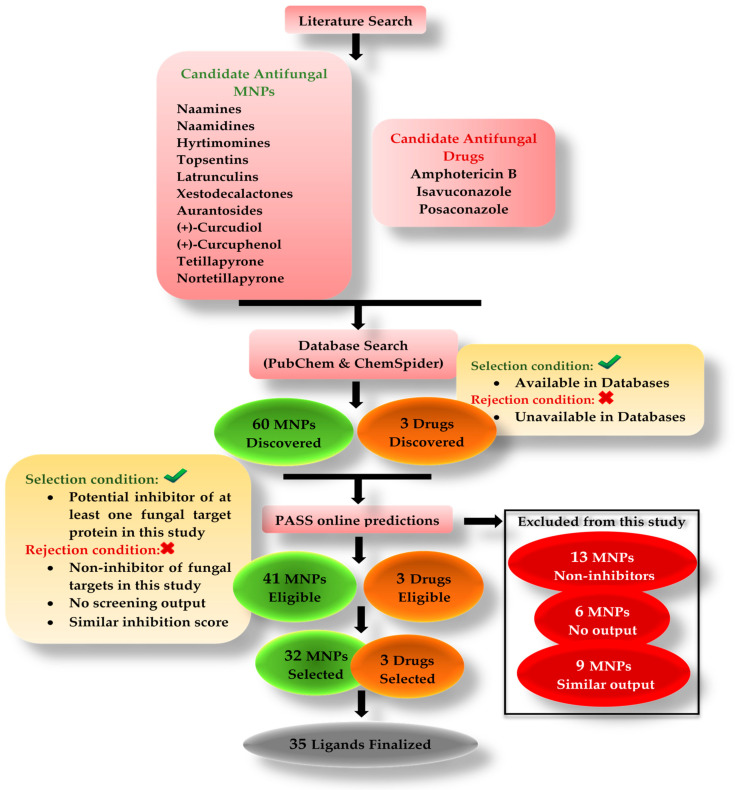
Ligand selection criteria—a schematic representation (MNPs—marine natural products).

The 60 MNPs that met these requirements were considered in this study. Thirteen MNPs failed to show inhibition potential, and six MNPs were inconclusive due to no PASS online output. In this case, 41 MNPs were eligible. The aurantoside group comprising 11 MNPs showed very close proximity in the PASS online inhibition score output. Thus, nine MNPs belonging to the aurantoside group were excluded (Table 2). Only aurantoside I and K were selected based on the top inhibition scores to avoid a lengthy study. For instance, among the group, aurantoside I had the highest probability of being active (pa) = 0.098 as a 1,3 beta glucan synthase inhibitor. On the other hand, aurantoside K had the highest probability of being active (pa) = 0.550 as a beta-glucuronidase inhibitor. It was noted that the majority of aurantosides are potential inhibitors of both 1,3 beta glucan synthase and a beta-glucuronidase. However, aurantoside G and J might not be capable of inhibiting 1,3 beta-glucan synthase. All in all, 32 MNPs were selected for this study (Figure 7). 

In the case of antifungal drugs, all three were qualified for the conditions and were proven effective against mucormycosis.

Approximately 69% out of the total selected MNPs were found to be potential inhibitors of one or more targets. The remaining 31% fell under non-inhibitors and no PASS online outcomes. Six MNPs belonging to the hyrtimomine group did not obtain results due to the nature of their SMILES line notations. However, because other hyrtimomine MNPs qualified as potential inhibitors, there is a 50% probability that these six MNPs might also act as potential inhibitors, but it is inconclusive. In our opinion, the reason for such a high percentage of potential target inhibitors is due to the fact that this study included proven antifungal MNPs only. In addition, a wide range of therapeutic targets was selected for this work. Thus, the probability of determining protein-target-neutralizing MNPs increased.

#### PASS Online Analysis

The PASS online server provided information about the biological activities of the marine-based ligands for docking studies. The table below presents the PASS online outcomes for the selected MNPs.

These compounds fit the scope of this study because not only do all the compounds have antifungal, antiviral, and anti-infective properties, certain compounds also offer additional beneficial properties. These properties consist of anti-asthmatic, anti-inflammatory, antiseptic, anti-eczematic, mucolytic, antibiotic, bronchodilator, mucositis treatment, and respiratory distress relief treatment. All the properties above might be beneficial in both fungal and viral infections such as mucormycosis and COVID-19. On the other hand, the biological activities of medical drugs appeared to be very specific. Only amphotericin B has potent antiviral activity. Nonetheless, it is an antifungal enhancer. Only the compounds in the latrunculin group were found to be antifungal enhancers.

Different target inhibition properties were also present in both compounds and drugs, as mentioned in Table 3. These include the kinase and histidine kinase inhibition, alpha and beta-glucuronidase inhibition, exo-1,3-beta-glucan-synthase inhibition, lanosterol 14 alpha-demethylase inhibition, rhizopuspepsin inhibition, and RNA-directed RNA polymerase (RdRp) inhibition. The medical drugs exhibit a similar inhibition profile. However, amphotericin B seemed suitable for target exo-1,3-beta-glucan-synthase. Isavuconazole and posaconazole work best against the target lanosterol 14 alpha-demethylase.

Tables S1 and S2 summarize the list of ligands chosen after the PASS online screening procedure, including their smiles line notations, PubChem CIDs, and ChemSpider IDs. The compounds were categorized based on chemical classification. The 3D chemical structures of the ligands and the medical drugs in Figures S2 and S3 were visualized in the BIOVIA Discovery Studio Visualizer. All 32 MNPs and drugs, based on the target specificity, are summarized in Table 4 below.

### 2.6. Molecular Docking and Interaction Studies

Molecular docking and interaction analysis helped determine binding affinities of the compounds with all seven target proteins under investigation. All 32 MNPs and 3 drugs were subjected to docking protocol. However, for docking output based on the PASS online screening, only the MNPs with the potential to inhibit their respective targets (in Table 4) are presented and discussed below. Binding affinities for other MNPs are presented (in Tables S3, S5, S7, S9, S11, S13 and S15). Additionally, all interacting amino acid residues for all ligands are mentioned (in Tables S4, S6, S8, S10, S12 and S14).

#### 2.6.1. CotH3

**Figure 8:**
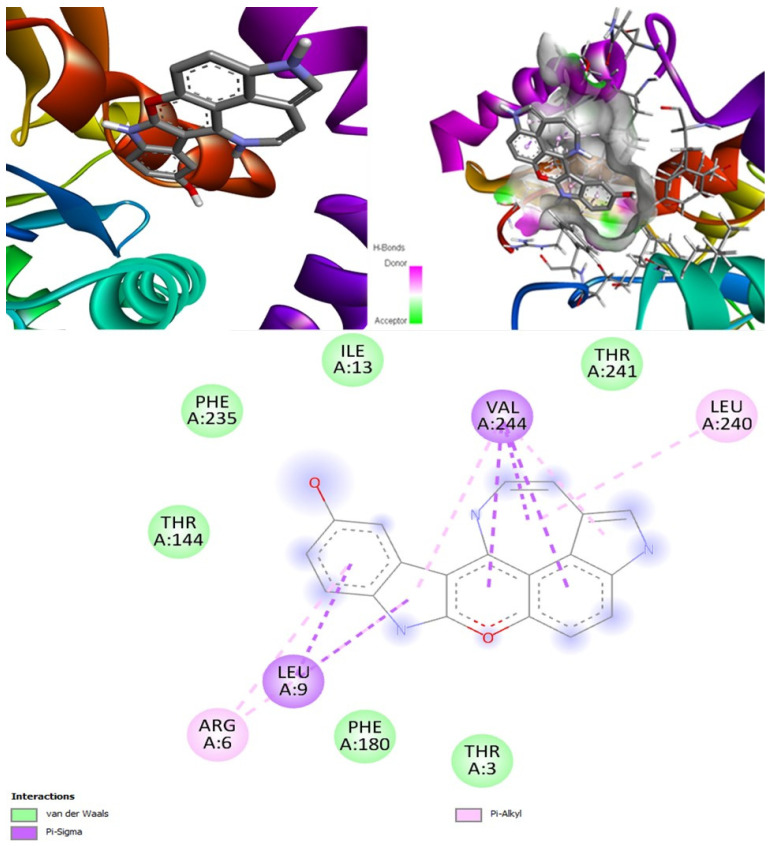
CotH3-hyrtimomine A docked complex and interaction map.

A recently published manuscript reported five compounds that might inhibit CotH3. Out of which two compounds such as 12, 28-oxamanzamine A with the binding affinity −10.2 kcal/mol and haliclonacyclamine B with an affinity of −9.2 kcal/mol can be considered the best identified so far [56]. Our molecular docking analysis revealed that hyrtimomine A had the highest binding affinity with the lowest energy, −9 kcal/mol (Figure 8). The second-best binding affinity was observed for deoxytopsentin/topsentin A with energy of −8.8 kcal/mol. This affinity value was close to that identified for deoxytopsentin from literature, −8.5 kcal/mol. The third-best interaction was for topsentin D with energy of −8.2 kcal/mol. The lowest binding affinity was observed for (+)-curcudiol with the highest energy value of −5.8 kcal/mol, closely followed by tetillapyrone and nortetillapyrone at −6.1 and −6.3 kcal/mol (Table 5). 

On the other hand, all three drug comparisons showed good binding affinities ranging from −8.8 to −8.0 kcal/mol. However, only isavuconazole is a potential inhibitor of CotH3, but hyrtimomine A outperformed the drug isavuconazole.

#### 2.6.2. Docking Mucoricin

**Figure 9:**
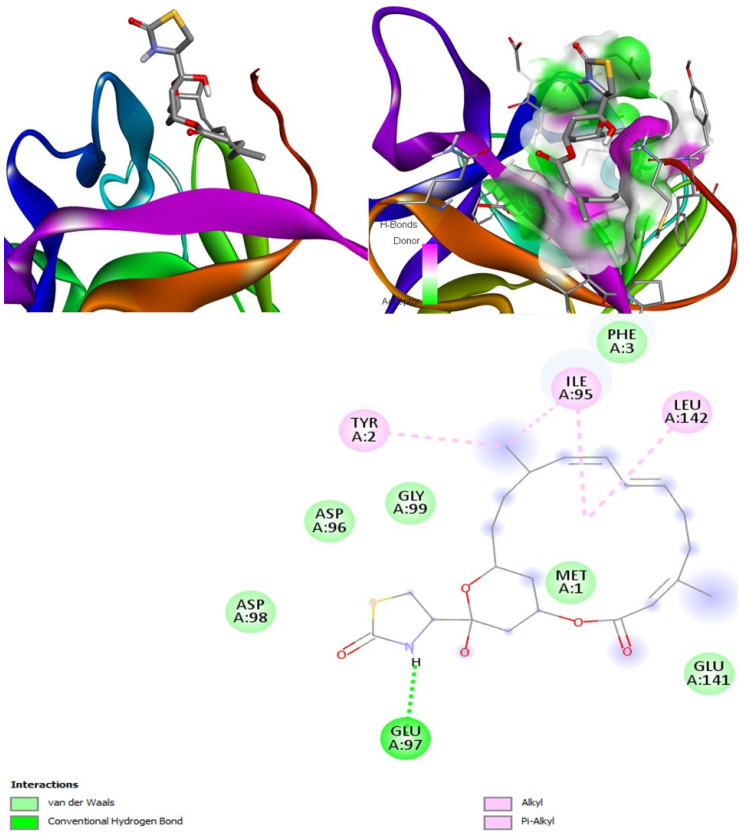
Mucoricin-latrunculin A docked complex and interaction map.

Molecular docking output for the target mucoricin summarized in Table 5 revealed the best binding affinity of latrunculin A with energy of −8.6 kcal/mol (Figure 9). From the literature, 12, 28-oxamanzamine A had shown a similar binding affinity [56]. The second-best binding affinity was shown by hyrtimomine B, −8.2 kcal/mol. Similar binding affinity was noticed for parsiguine, halicyclamine A, and tetrahydrohaliclonacyclamine A, in the literature. The third-best binding score was seen in the case of hesperidin (8.0 kcal/mol), identified in the literature [56]. This was followed by naamine E and hyrtimomine G with −7.6 kcal/mol. The worst-performing compound was again (+)-curcudiol at −5.9 kcal/mol (Table 6).

In the drugs category, posaconazole showed the best binding affinity of −7.8 kcal/mol. However, none of the three drugs showed the potential to inhibit the enzyme mucoricin. Thus, latrunculin A showed comparatively better binding, and hence, it was considered the best-performing MNP with regards to the target, mucoricin.

#### 2.6.3. exo-1,3-beta-glucan Synthase

**Figure 10:**
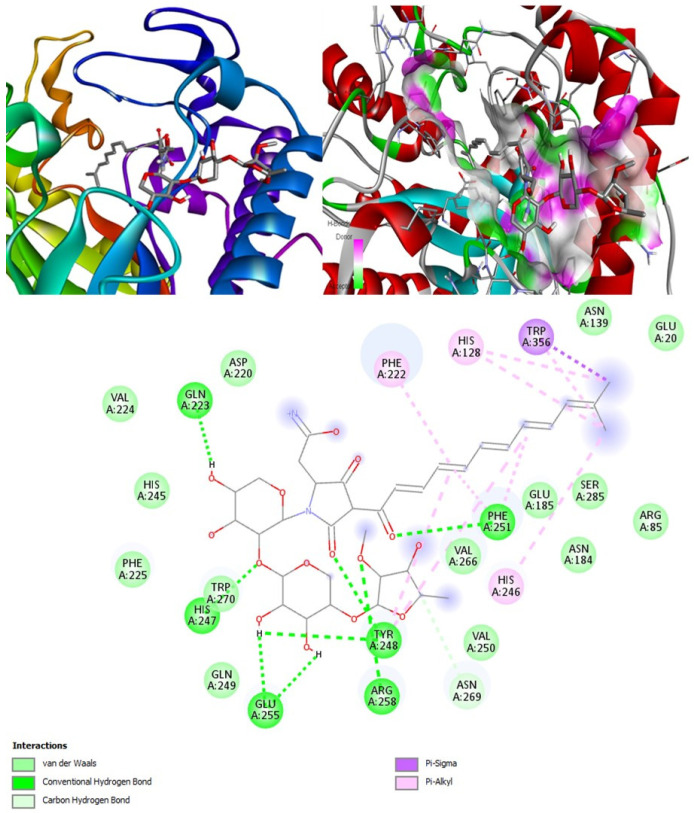
exo-1,3-beta-glucan synthase-aurantoside I docked complex and interaction map.

Molecular docking output for the target exo-1,3-beta-glucan synthase in Table 7 indicated that aurantoside I had the highest binding affinity energy of −11.4 kcal/mol (Figure 10). Aurantoside K scored the second-best binding affinity of −8.9 kcal/mol, followed by (+)-curcuphenol at −8 kcal/mol. The fourth- and fifth-best binding affinities were showcased by tetillapyrone and nortetillapyrone at −7.8 and −7.7 kcal/mol, respectively. The least binding affinity was demonstrated by (+)-curcudiol at −7.4 kcal/mol (Table 7). One compound identified from the literature, 1, 8 cineole docked with exo-1,3 glucan beta synthase had shown good binding affinity [57]. However, it might not be capable of inhibiting this target. According to the PASS online program, this compound is a potential inhibitor of rhizopuspepsin enzyme only.

Concerning the drug’s performance, posaconazole showed the best binding affinity of −10.8 kcal/mol. However, it might be incapable of inhibiting exo-1,3-beta-glucan synthase. Amphotericin B, on the other hand, with the binding affinity of −9.4 kcal/mol, is more likely to inhibit this enzyme, as observed during the PASS online analysis. Aurantoside I outperformed all compounds and the drugs and is considered the best for the target exo-1,3-beta-glucan synthases.

#### 2.6.4. Docking RNA-Directed RNA Polymerase (RdRp)

**Figure 11:**
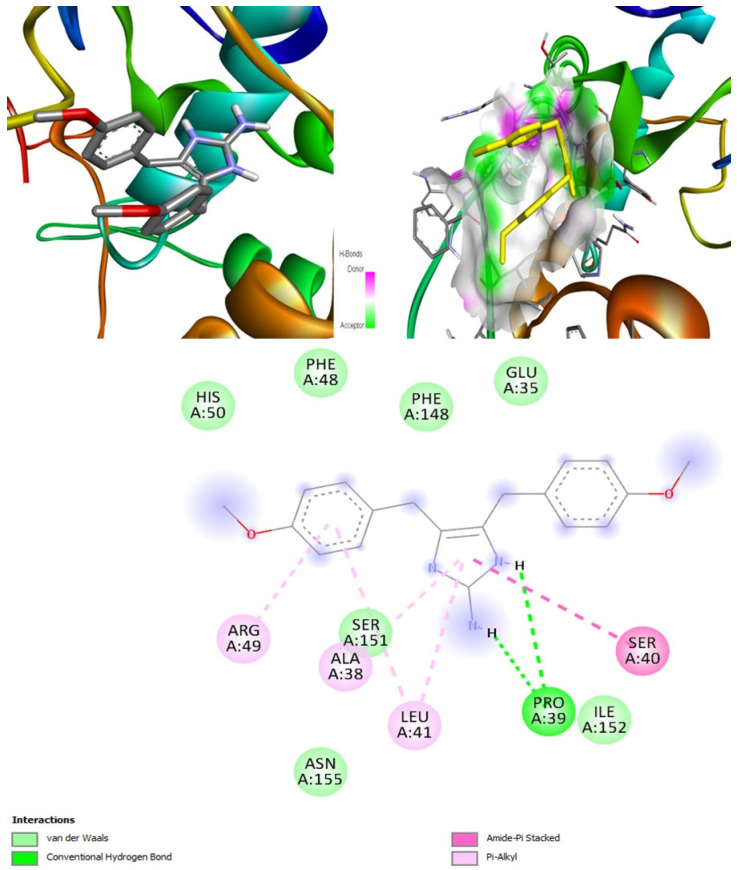
RdRp-naamine D docked complex and interaction map.

Naamine D showed the best binding affinity for molecular docking output for the target RdRp with energy of −8.8 kcal/mol (Figure 11). The second-best binding affinity was observed for hyrtimomine G, with energy of −7.1 kcal/mol. The third-best binding affinity value had tetillapyrone with energy of −6.5 kcal/mol, which was closely followed by (+)-curcuphenol with energy of −6.3 kcal/mol. The least binding affinity was seen for (+)-curcudiol and nortetillapyrone at −6.1 kcal/mol (Table 8). Two antiviral medications, sofosbuvir and ribavirin, were found in the literature which were docked with fungal RdRp [58]. These two compounds showed an average binding affinity of −6.1 and −6.6 kcal/mol, respectively. However, upon PASS online analysis, it was found that the drug ribavirin might also inhibit mucoricin and 1,3 beta glucan synthase.

Among the three drugs, amphotericin B was the best, with an affinity of −8.6 kcal/mol; however, no drug was found to be an inhibitor of this particular enzyme. Thus, naamine D has outperformed all the drugs and the compounds. 

#### 2.6.5. Docking Rhizopuspepsin

**Figure 12:**
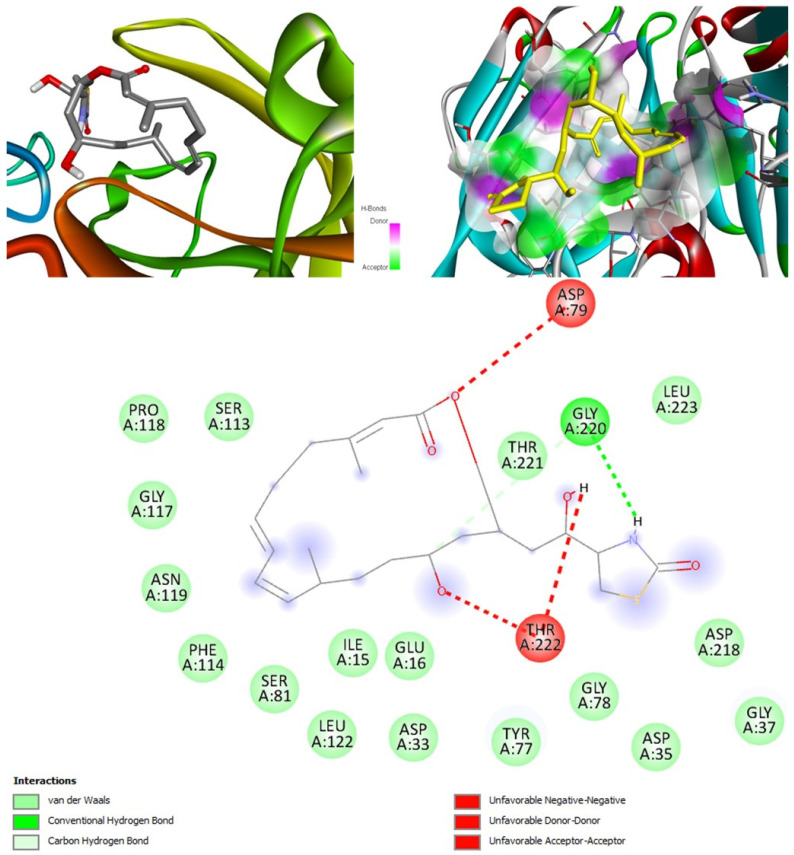
Rhizopuspepsin-latrunculin S docked complex and interaction map.

The molecular docking output for rhizopuspepsin as the target revealed latrunculin S as having the highest binding affinity of −9.8 kcal/mol (Figure 12). The second-best binding affinity was shown by hyrtimomine G with −8.4 kcal/mol. The third-best affinity was for (+)-curcuphenol with energy of −6.7 kcal/mol. The least binding affinity was observed for naamine D, −6.3 kcal/mol (Table 9). Three compounds identified from literature docked with rhizopuspepsin such as cajanone, diosgenin and piperine showed binding affinity of −9.1, −8.7, and −7.8 kcal/mol [59]. However, none of the three are capable of inhibiting rhizopuspepsin enzyme, as indicated by PASS online analysis.

Posaconazole showed the best affinity amongst the drug group, −8.7 kcal/mol, closely followed by amphotericin B, −8.6 kcal/mol. Thus, many MNPs have shown significantly better affinity to rhizopuspepsin compared to the drugs, with latrunculin S being the best.

#### 2.6.6. Lanosterol 14 Alpha-Demethylase

**Figure 13:**
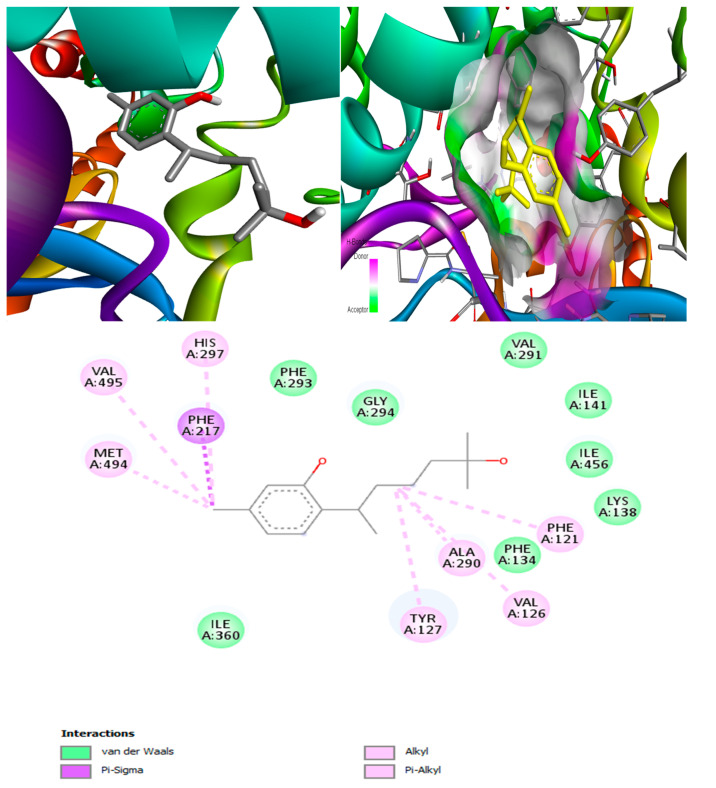
Lanosterol 14 alpha-demethylase-(+)-curcudiol docked complex and interaction map.

Only two MNPs in this study were found to be effective against this target [56]. The molecular docking output for the target lanosterol 14 alpha-demethylase showed that (+)-curcudiol had the best binding affinity of −11.4 kcal/mol (Figure 13). Pramiconazole, identified in the literature, also showed a similar binding affinity. This was followed by 12,28-oxomanzamine A, fascioquinol D, saperconazole, and finally, fascioquinol C. (+)-Curcuphenol in our study also showed good binding affinity, but certainly not the best (Table 10). All ligands mentioned in the table above are capable of inhibiting lanosterol 14 alpha demethylase enzyme.

The worst-performing drug with this target was amphotericin B, with the binding affinity 44.2 kcal/mol. This indicated that amphotericin B is not suitable for this particular target. Moreover, it might not inhibit the lanosterol 14 alpha demethylase enzyme, while isavuconazole showed strong binding affinity, −9.0 kcal/mol, closely followed by posaconazole with energy of −8.8 kcal/mol. These two drugs are potential inhibitors of this enzyme. In the end, (+)-curcudiol was found to be the best candidate.

#### 2.6.7. Docking Fungal Lipase

**Figure 14:**
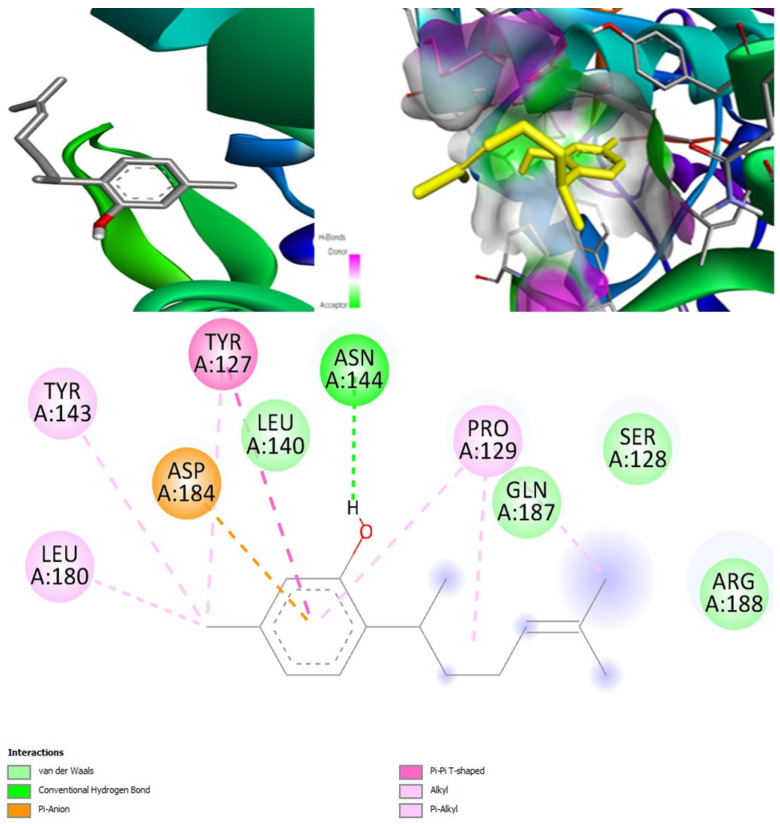
Fungal lipase-(+)-curcuphenol docked complex and interaction map.

For the fungal lipase target, the most suitable candidate was found to be (+)-curcuphenol, as it had the highest binding affinity of −8 kcal/mol (Figure 14). The binding affinity of (+)-curcudiol was low, −5.6 kcal/mol. Three other compounds found in the literature such as cajanone, diosgenin, and piperine docked with fungal lipase showed above average binding affinity of −7.6, −8.1, and −6.6 kcal/mol [59]. However, PASS online program suggests that these compounds might be incapable of inhibiting fungal lipase enzyme. Cajanone and diosgenin were found to be potential inhibitors of CotH3 and mucoricin. However, diosgenin is a strong RdRp stimulant, which can be problematic. Finally, the piperine was found to be an inhibitor of mucoricin only.

Posaconazole showed the highest binding affinity of −7.8 kcal/mol when compared to isavuconazole and amphotericin B. Nevertheless, it was slightly lower than the best-validated compound (+)-curcuphenol (Table 11). None of the three drugs are inhibitors of fungal lipase.

To summarize, the ligands identified from the literature that are potentially effective against CotH3, mucoricin and lanosterol 14 alpha-demethylase are relevant as they have good binding affinities as well as potential to inhibit the targets that can be confirmed by PASS online analysis. The docking values obtained for the compounds in the literature are not directly comparable to our values because different software and docking parameters can influence the output. Moreover, the source of the modeled protein structures and the method by which the energy minimization was performed may change the final outcome. Other compounds identified from the literature for exo-1,3-beta-glucan synthase, RdRp, rhizopuspepsin, and fungal lipase are not relevant. They have shown good binding affinities, but PASS online screening showed that they might not inhibit the docking targets. For the first time, this paper suggests cogent candidates from marine sponges against the abovementioned four targets to tackle mucormycosis.

Based on the molecular docking outcomes in this study, the top ranking docked complexes are summarized in Table 12. These docked complexes were further subjected to rapid molecular dynamic simulation in IMODS server. The selection of docked complex for simulation was on the basis of the highest binding affinity and the potential to inhibit the respective target.

#### 2.6.8. Molecular Dynamic Simulations

The IMODS server utilizes the normal mode analysis (NMA) to determine the stability of top-ranked docked complexes based on internal coordinates. The stability is presented in the form of graphs describing several factors such as deformability, B-factors, eigenvalues, covariance maps, and elastic network models.

##### CotH3-hyrtimomine A Complex

**Figure 15:**
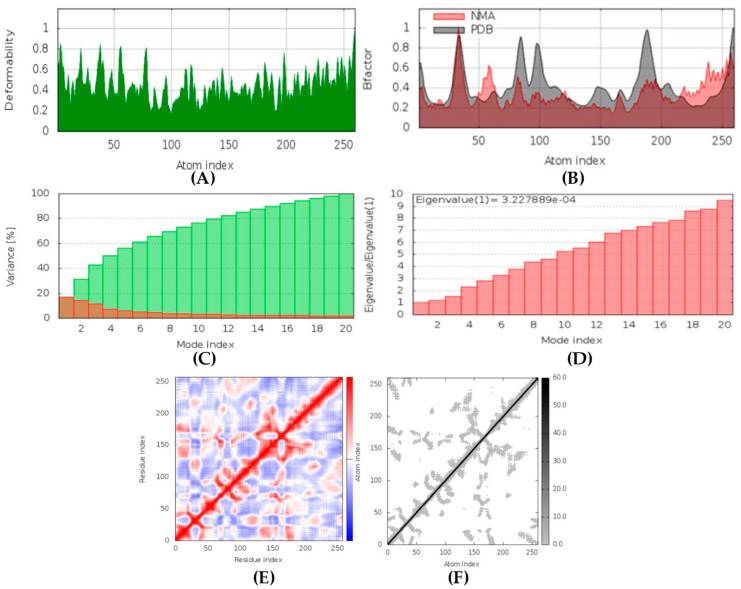
MD simulation output for CotH3-hyrtimomine A docked complex: (**A**) deformability; (**B**) B-factor; (**C**) variance; (**D**) eigenvalue; (**E**) covariance map; (**F**) elastic network model.

In MD simulation output of CotH3-hyrtimomine A docked complex formation (Figure 15), the main chain deformability graph shows levels of deformability for all residues, indicating medium to high deformability. The pointed regions called hinges are the points where the protein CotH3 and hyrtimomine A interacted with each other. The B-factor graph shows peaks for the B-factor and the NMA simulations. The pattern of the peaks obtained is almost similar, which means that the simulation results and the experimental results from PDB are similar. Next, the graph of variance shows that the individual variance level is higher (approx. 18%), which means that the eigenvalue is lower. The eigenvalue is indicative of the motion stiffness of the protein residues and displays the energy required to deform the structure. The eigenvalue obtained for this complex was 3.2 × 10^−4^, which can be considered low. This indicates that the interaction between CotH3 and hyrtimomine A was very good. Thus, stable complex was formed. Further, the covariance map shows that this complex had almost equal interacting and non-interacting residues as it shows prominent dark red and blue colors. The elastic network graph indicates the stiffness of the target CotH3. This protein can be readily deformed because the stiffness indicated was low, as is displayed by the light grey color.

##### Mucoricin-latrunculin A Complex

**Figure 16:**
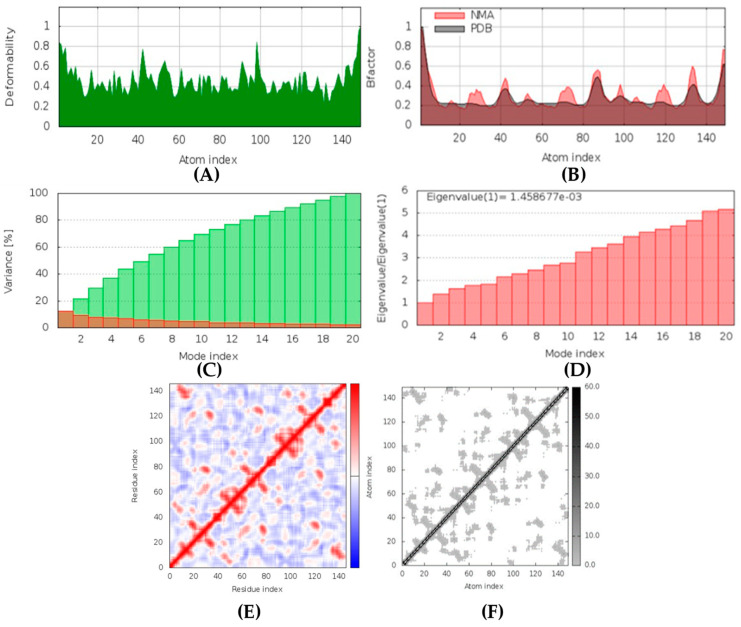
MD simulation output for mucoricin-latrunculin A docked complex: (**A**) deformability; (**B**) B-factor; (**C**) variance; (**D**) eigenvalue; (**E**) covariance map; (**F**) elastic network model.

In MD simulation output of mucoricin-latrunculin A docked complex, the main chain deformability graph indicates very high deformability (Figure 16). The pointed regions called hinges are the points where the protein mucoricin and latrunculin A interacted with each other. The B-factor graph indicates that the peak patterns obtained are very similar. The graph of variance shows that the individual variance level is high (approx. 16%), which indicates a lower eigenvalue. The eigenvalue obtained for this complex was 1.4 × 10^−3^; this can be considered very low. It indicates that the interaction between mucoricin and latrunculin A was excellent, forming a stable complex. Further, the covariance map shows that this complex had a mix of more high and low interactive residues as compared to non-interacting ones as it shows prominent dark red and light blue colors. The elastic network graph indicates that the stiffness for the target mucoricin was very low as light grey color can be observed, indicating that this protein can be easily deformed.

##### exo-1,3-beta-glucan Synthase-aurantoside I Complex

**Figure 17:**
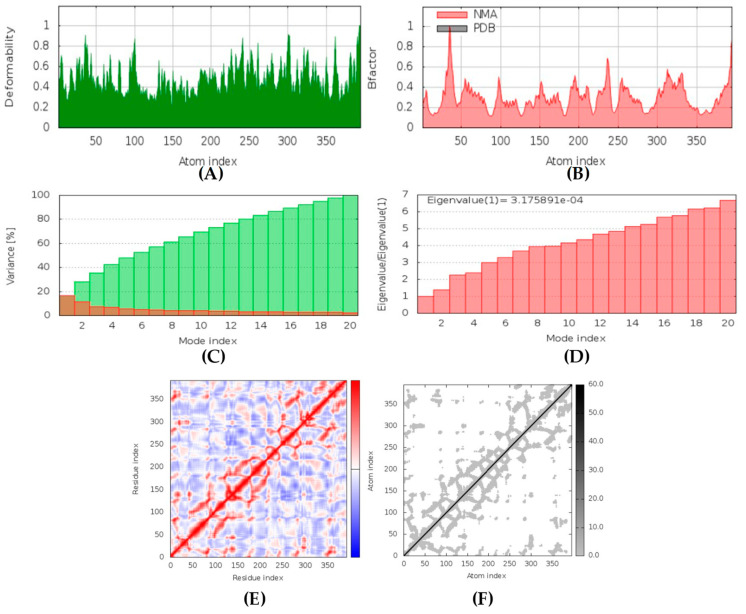
MD simulation output for exo-1,3-beta-glucan synthase-aurantoside I docked complex: (**A**) deformability; (**B**) B-factor; (**C**) variance; (**D**) eigenvalue; (**E**) covariance map; (**F**) elastic network model.

In MD simulation output for exo-1,3-beta-glucan synthase-aurantoside I docked complex formation (Figure 17), the main chain deformability graph indicates very high deformability. The pointed regions called hinges are the points where the protein exo-1,3-beta-glucan synthase, and aurantoside I interacted with each other. The B-factor graph did not indicate the peak patterns for the target (PDB ID: 4M80) as some PDB models may not have B-factors. The graph of variance shows that the individual variance level was high (approx. 18%), which indicates a low eigenvalue. The eigenvalue obtained for this complex was 3.1 × 10^−4^; this can be considered low. It indicates that the interaction between exo-1,3-beta-glucan synthase and aurantoside I was very good, thus forming a stable complex. Further, the covariance map shows that this complex had balanced high and low interactive residues as it shows dark red and white colors. The elastic network graph indicates that the stiffness for the target exo-1,3-beta-glucan synthase was very low as light grey color can be observed. Thus, this protein can be easily deformed. 

##### RdRp-naamine D Complex

**Figure 18:**
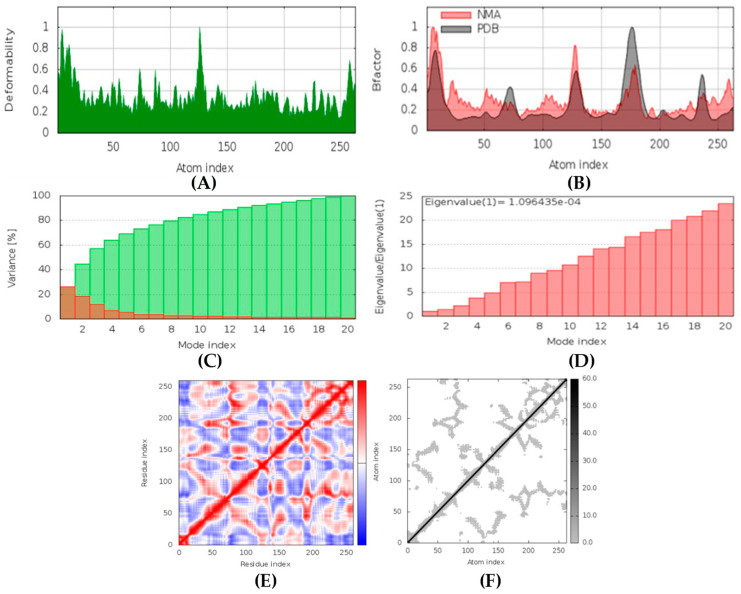
MD simulation output for RdRp-naamine D docked complex: (**A**) deformability; (**B**) B-factor; (**C**) variance; (**D**) eigenvalue; (**E**) covariance map; (**F**) elastic network model.

In MD simulation output of RdRp-naamine D docked complex, the main chain deformability graph indicates overall high deformability (Figure 18). The pointed regions called hinges are the points where the RdRp and naamine D interacted with each other. The B-factor graph indicates that the peak patterns obtained are somewhat similar. The graph of variance shows that the individual variance level is high (approx. 25–30% range), which indicates a lower eigenvalue. The eigenvalue obtained for this complex was 1.09 × 10^−4^; this can be considered very low. It indicates that the interaction between RdRp and naamine D was excellent. Thus, a very stable complex was formed. Further, the covariance map shows that this complex had a higher number of interacting and non-interacting residues as it shows prominent dark red and dark blue colors. The elastic network graph indicates that the stiffness for the target RdRp was extremely low as light grey color can be observed, revealing that this protein can be very easily deformed. 

##### Rhizopuspepsin-latrunculin S Complex

**Figure 19:**
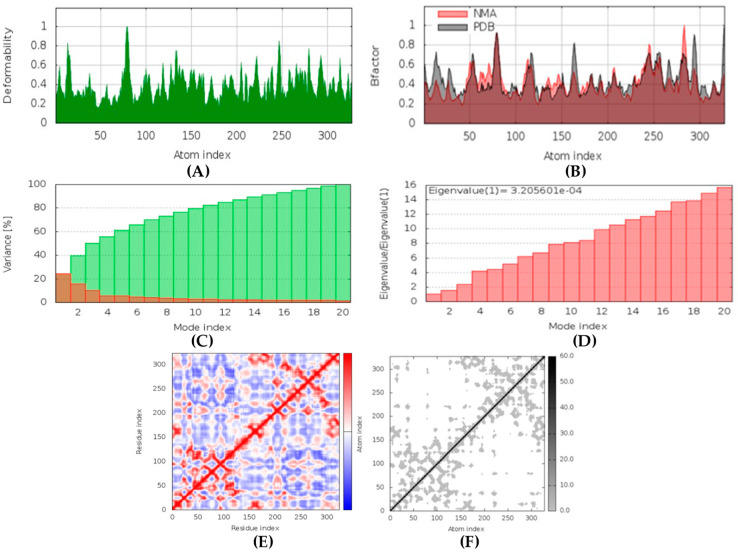
MD simulation output for rhizopuspepsin-latrunculin S docked complex: (**A**) deformability; (**B**) B-factor; (**C**) variance; (**D**) eigenvalue; (**E**) covariance map; (**F**) elastic network model.

In MD simulation output of rhizopuspepsin-latrunculin S docked complex (Figure 19), the main chain deformability graph indicates overall high deformability. The pointed regions, called hinges, are the points where the rhizopuspepsin and latrunculin S interacted with each other. The B-factor graph indicates that the peak patterns obtained are extremely similar. The graph of variance shows that the individual variance level is high (approx. 20–25% range), which indicates a lower eigenvalue. The eigenvalue obtained for this complex was 3.2 × 10^−4^; this can be considered low. It indicates that the interaction between rhizopuspepsin and latrunculin S was plausible, forming a very stable complex. Further, the covariance map shows that this complex had a comparatively higher number of low and non-interactive residues than high interactive residues, as it shows prominent dark blue and red colors. The elastic network graph indicates that the stiffness for the target rhizopuspepsin was low, as light grey color can be observed. Thus, this protein can be very easily deformed. 

##### Lanosterol 14 alpha-demethylase-(+)-curcudiol Complex

**Figure 20:**
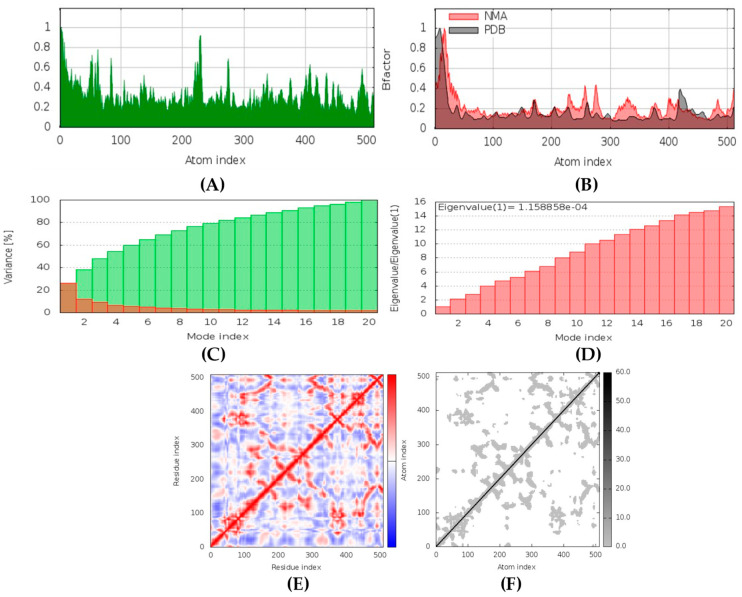
MD simulation output for lanosterol 14 alpha-demethylase-(+)-curcudiol docked complex: (**A**) deformability; (**B**) B-factor; (**C**) variance; (**D**) eigenvalue; (**E**) covariance map; (**F**) elastic network model.

In MD simulation output of lanosterol 14 alpha-demethylase-(+)-curcudiol docked complex (Figure 20), the main chain deformability graph indicates overall low deformability. The pointed regions called hinges are the points where the lanosterol 14 alpha-demethylase and (+)-curcudiol interacted with each other. The B-factor graph indicates that the peak patterns obtained are very similar. The graph of variance shows that the individual variance level is high (approx. 25–30% range), which indicates a lower eigenvalue. The eigenvalue obtained for this complex was 1.15 × 10^−4^ and can be considered very low. It indicates that the interaction between lanosterol 14 alpha-demethylase and (+)-curcudiol was excellent, thus forming a very stable complex. Further, the covariance map shows that this complex had a higher number of interacting and non-interacting residues as it shows prominent dark red and dark blue colors. The elastic network graph indicates that the stiffness for the target lanosterol 14 alpha-demethylase was extremely low as light grey color can be observed. Thus, this protein can be very easily deformed. 

##### Fungal Lipase-(+)-curcuphenol Complex

**Figure 21:**
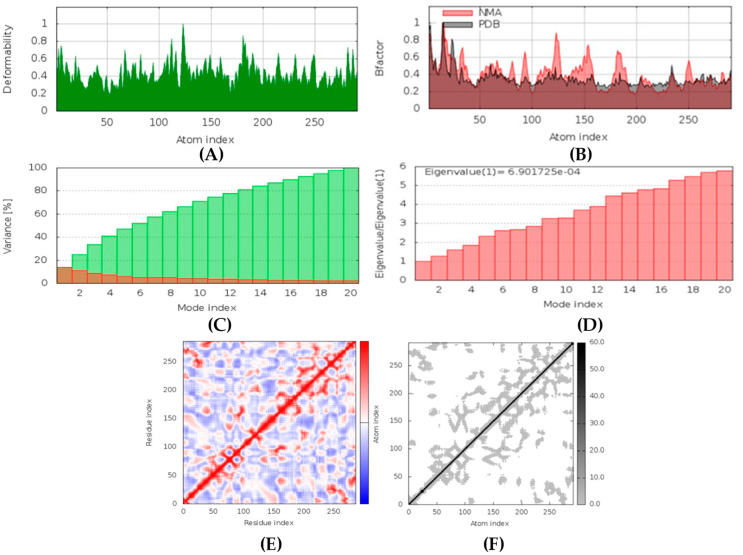
MD simulation output for fungal lipase-(+)-curcuphenol docked complex: (**A**) deformability; (**B**) B-factor; (**C**) variance; (**D**) eigenvalue; (**E**) covariance map; (**F**) elastic network model.

In MD simulation output of fungal lipase-(+)-curcuphenol docked complex (Figure 21), the main chain deformability graph indicates overall high deformability. The pointed regions called hinges are the points where the fungal lipase and (+)-curcuphenol interact with each other. The B-factor graph indicates that the peak patterns obtained are almost similar. The graph of variance shows that the individual variance level was high (approx. 15–18%), which indicated a higher eigenvalue. The eigenvalue obtained for this complex was the highest of all complexes, 6.9 × 10^−4^; this can be considered high. It indicates that the interaction between fungal lipase and (+)-curcuphenol is probably good but not the best. Thus, a comparatively less stable complex was formed. Further, the covariance map shows that this complex had a majority of highly interactive and low interactive residues as it shows prominent dark red and white colors. The elastic network graph indicates that the stiffness for the target fungal lipase was low as light grey color can be observed. Thus, this protein can be easily deformed.

### 2.7. Drug-Likeness Assessment

#### 2.7.1. Lipinski’s Rule of Five Analysis

Lipinski’s rule of five (RO5) parameters were validated to examine drug-likeness (Table 13 and Table S17). The first parameter is the molecular weight limit (MW ≤ 500). Out of seven top-performing ligands, six MNPs followed Lipinski parameters. The molecular weight of the majority of the other compounds is lower than 500 g/mol, but aurantoside I and K did not follow this rule. In the case of medical drugs, amphotericin B and posaconazole also failed this parameter as the molecular weight exceeded the limit. The second and the third parameters limit the H bond donors (HBD ≤ 5) and acceptors (HBA ≤ 10), and 30 out of 32 compounds passed the condition. Again, aurantosides I and K failed this condition. Likewise, amphotericin B also did not qualify because the hydrogen bond donors were over 5 and the hydrogen bond acceptors exceeded the maximum limit of 10. The final parameter is the limit of log *p* value (log *p* ≤ 5); all compounds and medical drugs did not exceed this value. However, negative values were found for nortetillapyrone, aurantoside I, and aurantoside K. Amphotericin B also possessed negative values, indicating that these compounds have higher affinities towards the aqueous phase. Compounds that followed all five rules do not entirely guarantee drug-likeness but have a higher probability of having higher oral bioavailability. Likewise, failure of these parameters also does not indicate a complete failure of drug potency.

Two additional drug-likeness parameters, such as the number of rotatable bonds (RB ≤ 10), and TPSA values [(Å^2^) ≤ 140)], provided more concrete estimations regarding the potential oral bioavailability. Thirty compounds except aurantoside I and K failed both the parameters as the number of rotatable bonds exceeded 10, and also the TPSA value was more than 140 Å^2^. The outcome was in line with the Lipinski rule of five. On the contrary, amphotericin B passed the parameter of rotatable bonds but failed the second parameter as the TPSA value was much higher. Moreover, posaconazole failed the rotatable bond condition. In summary, amphotericin B, posaconazole, and aurantoside I and K cannot be considered suitable for oral administration.

#### 2.7.2. Swiss-ADME Output

**Figure 22:**
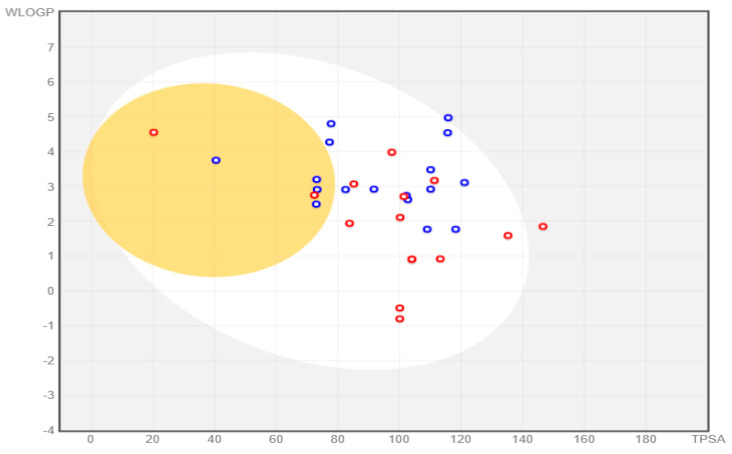
Swiss-ADME boiled egg graph showing permeability of all 35 compounds.

Swiss-ADME parameters revealed additional characteristics of the examined compounds (Table 14 and Table S18). Naamine D, G, naamidine A, B, C, hyrtimomine A–C, and the topsentin group had the worst solubility. Naamine A, B, E, F, (+)-curcudiol, and (+)-curcuphenol are not readily soluble. Hyrtimomine F and G are moderately soluble. Latrunculin A, B, S, xestodecalactone A, B, C, F, tetillapyrone, nortetillapyrone, and aurantoside I and K were found to be highly soluble. In the case of medical drugs, only amphotericin B appeared to be highly soluble. The other two drugs are poorly soluble. In total, 30 out of 32 compounds showed high bioavailability scores. Hyrtimomine B, in particular, had the highest score among all; whereas aurantoside I and K had the lowest score for oral bioavailability. Amphotericin B and posaconazole had a low bioavailability score among the drugs category, but it was higher than aurantosides. Isavuconazole, on the other hand, had the highest score in the drug category. The GI absorption was high for all MNPs except the aurantosides and hyrtimomine G. Amphotericin B had low absorption whereas posaconazole had high absorption. In the case of isavuconazole, despite a high bioavailability score, the absorption was found to be low. Figure 22 shows the boiled egg graph (plotted with TPSA on the *X*-axis and LogP on the *Y*-axis) obtained for all the compounds, including the drugs. Only 6 out of 35 compounds (including three drugs) were found to be capable of crossing the blood-brain barrier (BBB). Twenty-seven compounds were capable of being absorbed passively by the GI tract; only isavuconazole and hyrtimomine G were found to be suitable for GI tract absorption.

#### 2.7.3. OSIRIS Analysis

Osiris analysis provided information regarding the compound’s probable ill-effects, such as mutagenicity, tumorigenicity, irritancy, and the ability to affect the reproductive system (Table 15 and Table S19).

The compounds such as xestodecalactone E and F, (+)-curcuphenol, and aurantoside I and K possess a high risk of being irritants. All other compounds and drugs were found to have a low risk. Only posaconazole was found to be of highly mutagenic and tumorigenic potential. Aurantosides I and K are highly capable of affecting the reproductive system. Furthermore, the drug-likeness score indicated druggability, meaning the higher the drug-likeness score, the better chance of the compound’s efficacy. The highest drug score was obtained for naamidine B and the lowest score was observed for latrunculin B. The drug score in OSIRIS is a cumulative value derived from the combination of clogP, logS, molecular weight, toxicity risks, and drug-likeness values. The lowest drug score was identified for aurantoside K among the list of compounds. On the other hand, the highest drug score was observed for naamine B. Posaconazole was the lowest of all in terms of the drug score. Overall, only 13 out of the 32 compounds were deemed to be non-druggable. In terms of medical drugs, amphotericin B and isavuconazole were also found to be non-druggable.

### 2.8. Toxicity Investigation

#### 2.8.1. ProTox-II

ProTox-II analysis is the classification of compounds into different toxicity classes based on the LD50 value, which is an estimation of the median lethal dose at which 50% of the test subjects die when exposed orally (Table 16 and Table S20). In addition, three parameters considered in pkCSM highlighted the potential organ toxicity, *T. pyriformis* toxicity, and minnow toxicity. The table below presents the obtained results from the said analyses.

According to the GHS standards, the compounds are allocated in six classes, with the highest class having the lower probability of being toxic and/or harmful (Table 16 and Table S20). Class 1 (LD50 ≤ 5) and 2 (5 < LD50 ≤ 50) denote that the compounds are deadly and most likely will cause fatality. None of the compounds in this study have been found to belong to classes 1 and 2. Class 3 (50 < LD50 ≤ 300), denotes possible toxicity risks upon oral exposure. Tetillapyrone, nortetillapyrone, and hyrtimomine G, as well as the drug amphotericin B, were found to be in class 3. Class 4 (300 < LD50 ≤ 2000) categorizes most likely harmful compounds when exposed orally. Among the list of 32 compounds, 19 compounds were classified in this group. Two drugs, isavuconazole, and posaconazole belonged to class 4. Class 5 (2000 < LD50 ≤ 5000) categorizes the compounds that are less likely to be harmful upon oral exposure. Ten compounds in the list fall in this category. Finally, class 6 (LD50 > 5000) represents the non-toxic compounds. No compounds in this study were found to be class 6 members. 

Additional toxicity parameters in pkCSM revealed that among 32 compounds, roughly half of them are capable of causing hepatotoxicity (Table 16 and Table S20). Among the drugs, only amphotericin B is not hepatotoxic. Further, *T. pyriformis* and minnow toxicity values indicated that all compounds had different thresholds to exhibit toxic effects on the ciliate *T. pyriformis* and fathead minnows. In comparison, only two compounds, (+)-curcudiol and (+)-curcuphenol at higher doses of 1.468 and 1.876 (log μg/L), respectively, might cause toxicity to the ciliate. Drugs require almost similar doses of 0.285 (log μg/L). Minnow toxicity outcomes showed that (+)-curcudiol causes toxicity at dose 0.175 (log mM), whereas (+)-curcuphenol received a negative value of −0.277 (log mM). Aurantoside I and K were found to be toxic at a much higher dose of 9.082 and 9.573 (log mM), respectively. Similarly, amphotericin B may become toxic to minnows at a dose of 11.261 (log mM). 

#### 2.8.2. Acute Toxicity Analysis

This analysis was to investigate whether the compounds are capable of causing any type of acute toxicity upon exposure.

All MNPs studied in this work did not show signs of acute inhalation toxicity (Table 17 and Table S21). Ten out of thirty-two compounds were found to be positive for acute oral toxicity. Moreover, the drug posaconazole can cause acute oral toxicity. In the case of dermal toxicity, among all the MNPs, only the xestodecalactones A, B, C, D, and F were found to be positive. Skin sensitization was predicted to be positive in the case of nine compounds belonging to the naamine group, naamine A, B, E, F, and G. In addition, xestodecalactone E and F, (+)-curcudiol, and (+)-curcuphenol might also inflict skin sensitization upon exposure. Finally, most of the compounds have shown the ability to cause irritation of either eyes or skin, while naamine A may inflict irritation on both. Only eight out of thirty-two compounds were found to be negative in both eyes and skin irritation and corrosion. 

**Table 17:** StopTox acute toxicity output for best ligands.

StopTox Acute Toxicity Analysis					
Ligands	Inhalation Toxicity	Oral Toxicity	Dermal Toxicity	Skin Sensitization	Irritation and Corrosion
Hyrtimomine A	No	No	No	No	Eyes (No), Skin (No)
Latrunculin A	No	No	No	No	Eyes (Yes), Skin (No)
Aurantoside I	No	No	No	No	Eyes (Yes), Skin (No)
Naamine D	No	Yes	No	No	Eyes (Yes), Skin (No)
Latrunculin S	No	No	No	No	Eyes (Yes), Skin (No)
(+)-Curcudiol	No	No	No	Yes	Eyes (Yes), Skin (No)
(+)-Curcuphenol	No	No	No	Yes	Eyes (Yes), Skin (No)
**Drugs**					
Amphotericin B	No	No	No	No	Eyes (Yes), Skin (No)
Isavuconazole	No	No	No	No	Eyes (Yes), Skin (No)
Posaconazole	No	Yes	No	No	Eyes (Yes), Skin (No)

## 3. Discussion

SARS-CoV-2 mortality and cases are much higher than originally thought [60]. This virus has shown flexibility in evolutionary terms and most likely may continue to exist for several years, in its current form or even in an evolved form [61]. As long as SARS-CoV-2 exists, the risk of mucormycosis infections might remain a subject of major concern. The world is now desperately looking for relief from the SARS-CoV-2 pandemic and mucormycosis epidemic. This paper proposes the idea of considering marine-sponge-based antifungal compounds against mucormycosis. 

According to the PASS online program, some compounds belonging to the naamine group were antifungal, anti-asthmatic, anti-infective, antiviral, and can be used for mucositis treatment. These features help deal with both viral and fungal infections. Naamines are also kinase inhibitors and histidine kinase inhibitors. This indicates that naamines can inhibit the target CotH3. Furthermore, they were found to inhibit the beta-glucuronidase enzyme. This makes them a potential inhibitor of mucoricin target. Moreover, they are capable of inhibiting rhizopuspepsin and the RdRp enzyme. Based on the molecular docking outcomes, naamine D had the highest binding affinity, −8.8 kcal/mol with RdRp. Molecular dynamic simulation in IMODS server also suggested that the RdRp-naamine D formed a stable complex. Drug-likeness analysis for naamines showed that they all followed Lipinski parameters, which makes them good drug candidates. Swiss-ADME outcome suggests that naamine A, B, E, and F are moderately soluble and only naamine D is poorly soluble. All naamines had a bioavailability of 0.55 and were highly absorbable by the GI tract. Naamine A, B, and D can permeate the blood-brain barrier. In addition, OSIRIS analysis showed that all naamines pose low to no risk of being an irritant, mutagenic agent, or tumorigenic agent, and they do not affect the reproductive system. ProTox-II indicated that naamines belonged to classes 4 and 5. PkCSM analysis labeled naamine A, B, F, G as being hepatotoxic. However, naamine D and E are not hepatotoxic. Naamines were not found to cause acute inhalation and dermal toxicity. However, they can cause acute oral toxicity. Naamine D does not cause skin sensitization, whereas other naamines may cause skin sensitization. All naamines can cause eye irritation and corrosion. 

Naamidines A, B, and C are shown to possess antifungal, anti-asthmatic, anti-eczematic, and antiviral properties. In addition, they are kinase and histidine kinase inhibitors with good inhibition of CotH3 protein. Molecular docking analysis showed that naamidines overall had good binding affinities with some of the targets. Drug-likeness analysis for naamidines showed that they both followed Lipinski parameter, highlighting the potential to become a drug. Swiss-ADME outcome suggests that naamidine A, B, and C are poorly soluble. All naamidines had a bioavailability of 0.55 and are highly absorbable by the GI tract. None of them can permeate the blood-brain barrier. OSIRIS analysis showed that both naamidines pose a low risk of being an irritant, mutagenic agent, or tumorigenic agent, and they do not affect the reproductive system. PkCSM analysis suggests that naamidines A, B, and C are hepatotoxic. ProTox-II indicates that naamidines are class 5 compounds. Moreover, the StopTox outcome suggests that they do not cause acute inhalation or dermal toxicity, and there is no indication of skin sensitization. However, naamidines might cause eye irritation and corrosion. Among all of them, only naamidine C might exhibit oral toxicity.

Hyrtimomines A, B, C, F, and G are potential antifungal, anti-infective, and anti-inflammatory agents that might help with COVID-19 and mucormycosis symptoms. They are strong inhibitors of kinase and histidine kinase (pa > 0.3 and pa > 0.7). In addition, they can also inhibit the beta-glucuronidase enzyme, indicating that they can inhibit both CotH3 and mucoricin proteins. The docking analysis showed that hyrtimomine A had the highest binding affinity, −9 kcal/mol, with CotH3 protein. MD simulation indicated that a stable complex was formed. Drug-likeness estimation suggests that hyrtimomines do not violate Lipinski’s rules. Thus, they are potential drug-like molecules. However, Swiss-ADME showed that they have poor solubility except hyrtimomine G. The bioavailability value was high, 0.55, and hyrtimomine B had 0.56. High GI absorption was indicated for hyrtimomine A, B, C, and F but not in the case of hyrtimomine G. No blood-brain barrier permeability was shown. OSIRIS analysis showed that hyrtimomines pose low risk of being an irritant or mutagenic agent, and they do not affect the reproductive system. However, hyrtimomine F might be tumorigenic. PkCSM analysis suggests that they are hepatotoxic. ProTox-II indicates that they belong to class 4, except hyrtimomine G, which is a class 3 MNP. StopTox found that they do not cause acute inhalation and dermal toxicity. Hyrtimomine C might be toxic in the case of oral exposure. Hyrtimomine B, C, F, and G might cause irritation and corrosion of eyes, while hyrtimomine A does not.

Topsentins were found to be antifungal and anti-infective. They are good inhibitors of kinase and histidine kinase which means they can inhibit CotH3 protein. Molinspiration analysis also confirmed the potential kinase inhibition potential of topsentins (Table S22). Topsentins group showed good binding affinities in the molecular docking analysis. Drug-likeness estimation suggests that topsentins do not violate Lipinski rules and, therefore, they might meet necessary drug requirements. However, Swiss-ADME showed that they have poor solubility and bioavailability value was high, 0.55. High GI absorption indicated that topsentin D is blood-brain-barrier permeable. OSIRIS analysis showed that topsentins pose low risk of being an irritant, mutagenic agent, or tumorigenic agent, and they do not affect the reproductive system. PkCSM analysis suggests that only topsentin D is hepatotoxic. ProTox-II indicates that they belong to class 4. StopTox found that topsentins might not cause acute inhalation and dermal toxicity. However, topsentin A and D can cause oral toxicity. In addition, topsentin A and D are expected to be non-corrosive to the eye and skin.

Latrunculin A, B, and S were found to be antifungal, anti-eczematic, antiviral, antibiotic, and antifungal enhancers. Apart from this, they can provide respiratory distress relief, making them a suitable candidate for COVID-19-associated mucormycosis. They are beta-glucuronidase and rhizopuspepsin inhibitors. Molecular docking showed that latrunculin S had the highest binding affinity of −9.8 kcal/mol with target rhizopuspepsin. Furthermore, latrunculin A had the highest binding affinity with the target mucoricin, −8.6 kcal/mol. MD simulations for both complexes were suggestive of a very stable conformation being formed. Drug-likeness estimation suggests that latrunculins do not violate Lipinski rules. Thus, they have drug-like properties. Swiss-ADME indicated that they have good solubility and bioavailability, 0.55. High GI absorption indicated that they are not able to cross the blood-brain barrier. OSIRIS analysis showed that latrunculins A, B, and S pose low to no risk of being an irritant, mutagenic agent, or tumorigenic agent, and they do not affect the reproductive system. PkCSM analysis suggests that latrunculin A, B, and S can be hepatotoxic. ProTox-II indicates that they belong to class 4. StopTox found that latrunculin should not cause acute inhalation or oral and dermal toxicity, but they can be corrosive to the eyes.

Xestodecalactones A, B, C, D, E, and F are predicted to be antifungal, anti-eczematic, antiviral, and anti-infective. In addition, they are kinase, histidine kinase, and beta-glucuronidase inhibitors; thus, they can inhibit CotH3 and mucoricin proteins. Molecular docking output for xestodecalactones was satisfactory. Drug-likeness estimation suggests that xestodecalactones do not violate Lipinski rules and are suitable to be called drug-like. Swiss-ADME analysis indicated that they have good solubility, except xestodecalactone E, which is moderately soluble. The bioavailability was 0.55, and GI absorption also indicated no potential to cross the blood-brain barrier. OSIRIS analysis showed xestodecalactones A, B, C, and D pose low to no risk of being an irritant, mutagenic agent, or tumorigenic agent, and they do not affect the reproductive system. However, E and F pose a high risk of being an irritant. PkCSM analysis suggests that xestodecalactones A, B, C, D, E, and F are non-hepatotoxic. ProTox-II indicates that the majority of them belong to class 4 and only xestodecalactone F is class 5. StopTox found that xestodecalactone A, B, and C should not cause skin sensitization, acute inhalation, and oral toxicity. However, xestodecalactone D might cause dermal toxicity. Xestodecalactone E might cause skin sensitization. Xestodecalactone F might cause both dermal toxicity and skin sensitization. Xestodecalactone A, B, and C can be corrosive to the eyes. On the other hand, D, E, and F should not cause eye and skin irritation and corrosion.

(+)-Curcudiol was found to be antifungal, anti-eczematic, antiviral, anti-infective, and antiseptic, and it is also a bronchodilator. In addition, it is histidine kinase, exo-1,3-beta-glucan synthase, RdRp, lipase, lanosterol 14 alpha-demethylase, and alpha and beta-glucuronidase inhibitor. This means it might inhibit six out of seven targets involved in this study. Molecular docking confirmed the suspicion; (+)-curcudiol had the highest binding affinity, −11.4 kcal/mol, with lanosterol 14 alpha-demethylase. MD simulation also suggested that the complex formed between the two was stable. Drug-likeness estimation suggested that (+)-curcudiol does not violate Lipinski rules: thus, it might have a drug-like tendency. Swiss-ADME indicated that it has moderate solubility and bioavailability, 0.55. High GI absorption indicated the ability to cross the blood-brain barrier. OSIRIS analysis showed (+)-curcudiol poses low to no risk of being an irritant, mutagenic agent, or tumorigenic agent, and it does not affect the reproductive system. PkCSM analysis suggests that (+)-curcudiol is non-hepatotoxic. ProTox-II indicates that it belongs to class 5. StopTox found that (+)-curcudiol might not cause acute inhalation or dermal and oral toxicity. However, it can cause skin sensitization. It is corrosive to the eyes and not the skin.

(+)-Curcuphenol was predicted to be antifungal, anti-eczematic, antiviral, anti-infective, anti-inflammatory, a bronchodilator, mucolytic, and antiseptic. In addition, (+)-curcuphenol is histidine kinase alpha and beta-glucuronidase, lanosterol 14 alpha-demethylase, lipase, exo-1,3-beta-glucan-synthase, RdRp, and rhizopuspepsin inhibitor. This compound has been found to have broad-spectrum activity, as it has the potential to inhibit all seven targets involved in this study. These properties could be very helpful in addressing the COVID pandemic era. Molecular docking studies showed that (+)-curcuphenol had a good binding affinity with fungal lipase target, −8 kcal/mol. However, MD simulation indicated that the complex formed might be slightly unstable. Drug-likeness estimation suggested that (+)-curcuphenol does not violate Lipinski rules, thus having drug-like tendencies. Swiss-ADME indicated that it has moderate solubility and bioavailability of 0.55. High GI absorption was indicated, with the ability to cross the blood-brain barrier. OSIRIS analysis showed (+)-curcuphenol can be an irritant. PkCSM analysis suggests that (+)-curcuphenol is non-hepatotoxic. ProTox-II indicates that it belongs to class 4. StopTox found that (+)-curcuphenol should not cause acute inhalation or dermal and oral toxicity, but it can cause skin sensitization. It can be corrosive to the eyes but not to the skin. 

Tetillapyrone and nortetillapyrone are potentially antifungal, anti-eczematic, antiviral, and anti-infective. In addition, they are histidine kinase, beta-glucuronidase, RdRp, and exo-1,3-beta-glucan-synthase inhibitors. This means they can inhibit CotH3, mucoricin, and two other target proteins. They showed mostly average and some good interactions in docking studies in comparison to other compounds. Both compounds do not violate Lipinski’s rules, thus having drug-like tendencies. They have high solubility and bioavailability of 0.55. Although a high GI absorption was indicated, they cannot cross the blood-brain barrier. OSIRIS analysis showed they are both harmless. PkCSM analysis suggests that they both are non-hepatotoxic. ProTox-II indicates that they belong to class 3. StopTox found that they might not cause acute inhalation or dermal and oral toxicity. In addition, they may not cause skin sensitization and corrosion of skin and eyes.

Aurantosides I and K are predicted to be antifungal, antiviral, antibiotic, and anti-inflammatory. In addition, they can inhibit beta-glucuronidase and exo-1,3-beta-glucan-synthase enzymes. Docking results have shown that the interaction between aurantoside I and exo-1,3-beta-glucan-synthase obtained the highest binding affinity value, −11.4 kcal/mol. MD simulations have also indicated the formation of a stable complex. According to Swiss-ADME, they both have high solubility, and the bioavailability was lowest compared to all other compounds. As a result, the GI tract absorption is also found below. They are not blood-brain-barrier permeable. OSIRIS output suggests they are potential irritants and might affect the reproductive system. PkCSM indicates aurantoside I might be hepatotoxic. They belong to class 5 compounds in ProTox-II. StopTox found that they should not cause acute inhalation, dermal and oral toxicity, or skin sensitization. However, they can cause irritation and corrosion of the eyes.

Concerning medical drugs in this study, amphotericin B was predicted to be an antifungal, antiviral, anti-inflammatory, and anti-infective enhancer. By contrast, isavuconazole and posaconazole are only antifungals. The mechanism of action of these drugs is destroying the fungal cell wall by inhibiting the synthesis of beta-glucan. Our calculations revealed that both isavuconazole and posaconazole inhibit a common target, lanosterol 14 alpha-demethylase, thus preventing the conversion of lanosterol into ergosterol, leading to the damage of the plasma membrane of the fungi. Additionally, isavuconazole might inhibit CotH3, thus being a kinase inhibitor, while posaconazole, in OSIRIS analysis, was found to be potentially mutagenic and tumorigenic.

All the compounds were categorized based on target-specific biological activity (see Table 4). The compounds in this study were shown to be capable of inhibiting different targets to varying degrees. We have found that some MNPs from sponges presented better target-specific inhibition as compared to the three antifungal drugs, highlighting their pharmaceutical potential. All 32 MNPs are worth investigating. However, (+)-curcudiol and (+)-curcuphenol showed promising potential to inhibit the majority of the fungal targets mentioned in this study. In addition, these MNPs showed satisfactory drug-likeness properties with the capability to permeate the blood-brain barrier. This is important as COVID-19-associated mucormycosis also affects the brain in some cases [62]. Moreover, the findings of this study suggest that they might pose overall low toxicity risks. We recommend further laboratory interventions and experimental investigations to recognize the true capability of target inhibition by in vitro and in vivo studies.

## 4. Materials and Methods

### 4.1. Targets and Ligands Retrieval

Information on relevant targets was obtained through published literature. Seven targets were considered in this research. In the RCSB-PDB database [63], the 3D structures were available for three of the seven selected targets, rhizopuspepsin (PDB ID: 1UH9), lipase (PDB ID: 6A0W), and exo-1,3-beta-glucan synthase (PDB ID: 4M80) [64,65,66,67]. However, in the case of the remaining four targets, such as RdRp (GenBank Accession ID: BAH03542.1) [58], cotH3 (NCBI Accession ID: EIE87171), lanosterol 14 alpha-demethylase (NCBI Accession ID: EIE87079), and mucoricin (NCBI Accession ID: EIE81863) [56,68], the structures were not available. To solve this problem, FASTA sequences for these targets were retrieved from the NCBI database [69] using respective accession IDs obtained during the literature search [56,57,58,59,67,68]. In the case of target RdRp, the FASTA sequence for the RVT_1 region was retrieved. Likewise, in the case of target cotH3, region CotH was obtained in the form of a FASTA sequence. Further, the trRosetta server was used for 3D structure prediction, utilizing the obtained FASTA sequences.

A thorough literature search was conducted to trace the potential antifungal MNPs from sponges and prescribed antifungal drugs that are currently used for mucormycosis treatment. Six different classes of MNPs such as alkaloids (naamines, naamidines, hyrtimomines, and topsentins), macrolides (latrunculins), bioactive metabolites (xestodecalactones), sesquiterpene phenols [(+)-curcudiol, (+)-curcuphenol], hydroxpyran-2-ones (tetillapyrone and nortetillapyrone), and finally tetramic acid glycosides (aurantosides) were considered for this study. MNPs not found in PubChem and ChemSpider databases were excluded. The PubChem [70] and ChemSpider databases were searched. Three-dimensional structures and SMILES line notations of available MNPs were obtained and used further for analysis. The potential bioactivity predictions of candidate MNPs were carried out using the PASS online program [71] to check their ability to inhibit the chosen fungal target proteins. A total of 32 out of 60 MNPs and three drugs were finalized as suitable ligands. The remaining 28 MNPs were excluded because of non-inhibitory potential and due to no PASS online output. MNPs with similarities in output were excluded to limit the size of this study. The screening and selection criteria are described in detail (Figure 7 and Table 2). In addition, potential ligands suggested in published papers against mucormycosis targets mentioned in this study were identified, compared, and discussed. 

### 4.2. Visualization Tools

All 3D structures for both proteins and ligands were visualized using UCSF Chimera 1.16 [72] and BIOVIA Discovery Studio Visualizer 4.0 [73] software applications.

### 4.3. Energy Minimization and Quality Check

The 3D structures obtained from trRosetta for the four targets were subjected to energy minimization (EM). EM was first performed in the YASARA [74] server using the YASARA force field and then in UCSF Chimera software by applying AMBER ff14SB force field for all predicted 3D structures. The energy minimization step was important to achieve structures with lower delta G conformations, making them suitable for docking procedures. The usefulness of 3D structures was determined by a structure analysis and verification server called PROCHECK [75,76]. Ramachandran and ERRAT plots were generated to check the quality of obtained models and their stereochemical properties. A comparison was carried out between the outcomes of YASARA server and Chimera software. In the end, only the best quality structures were selected for further analysis.

### 4.4. Active Site Prediction

CAST-p stands for computed atlas surface topography of proteins. CAST-p is a web server [77] specifically used to predict the volume, area, and shape of active sites for ligand binding. This server was used to determine the location and the surface area of the active site for all targets. Protein targets were uploaded on the server in the PDB format. The probe radius was set to the default setting, 1.4 angstroms. The outcomes proved to be essential to conduct accurate docking procedures. 

### 4.5. Molecular Docking

The 3D structures of targets were prepared by removing water molecules and unnecessary chemical complexes, adding required charges and hydrogen bonds, and correcting bonds and missing side chains using the UCSF Chimera software. Molecular docking studies were performed in an automated fashion using PyRx 0.8 software [78]. All targets were loaded in the software and were set as macromolecules. Then, all 35 ligands were loaded for preparation to be docked with the macromolecules in the PDBQT format. Based on the results of CAST-p, grid boxes were set according to the location of active sites. The position of the grid box: rhizopuspepsin (*X* = 39.661, *Y* = 62.896, *Z* = 101.851), lipase (*X* = −39.135, *Y* = −16.661, *Z* = 12.511), exo-1,3-beta-glucan synthase (*X* = 3.308, *Y* = 68.613, *Z* = 9.839), RdRp (*X* = −4.429, *Y* = 5.902, *Z* = −20.215), cotH3 (*X* = 1.099, *Y* = −8.896, *Z* = −7.961), lanosterol 14 alpha-demethylase (*X* = 82.286, *Y* = 53.711, *Z* = 65.410), mucoricin (*X* = 12.549, *Y* = 3.752, *Z* = −10.521). Docking procedure was repeated over ten times with exhaustiveness set at 8. Two-dimensional and three-dimensional interaction maps were generated using BIOVIA Discovery Studio Visualizer.

### 4.6. MD Simulations

Protein-ligand complexes with the highest binding affinities were further analyzed using the IMODS server [79]. IMODS is a web server that was used to perform rapid molecular dynamic simulations of the selected complexes. All seven top-ranking docked complexes were uploaded in the pdb format to the server. The coarse-grained level for the atomic model was Cα-based, which was a default parameter used to run all simulations. This analysis provided the deformability, B-factors, eigenvalues, covariance maps, and elastic network models for the residues (derived from amber94 force field) involved in the interaction on the basis of NMA mobility. IMODS estimated the stability of complexes based on detailed coordinate investigation. 

### 4.7. Drug-Likeness Physicochemical Property Analysis

The drug-likeness properties for all compounds were determined using Swiss-ADME [80] server by inserting canonical SMILES. Under the Lipinski rules of five frameworks, four parameters (Ro5) were considered. The first is the molecular weight (MW ≤ 500). The second is the consensus log *p*-value (log *p* ≤ 5). The third is the number of hydrogen bond donors (HBD ≤ 5). The fourth is the number of hydrogen bond acceptors (HBA ≤ 10). Passing these parameters does not necessarily indicate that the compounds are orally active. Therefore, additional descriptors of drug-likeness were considered, such as topological polar surface area (TPSA ≤ 140), and the number of rotatable bonds (RB ≤ 10), which can provide better estimates on the probability of being active. Furthermore, the probability of the compounds being an irritant, mutagenic agent, or tumorigenic agent, and their reproductive effectivity in nature was determined using OSIRIS software obtained from the organic chemistry portal [81].

### 4.8. Toxicity Prediction

The compounds were first subjected to toxicity analysis to spot potential toxic properties using ProTox-II and pkCSM servers [82,83]. ProTox-II server provided the LD50 values in mg/kg body weight and toxicity classes for the compounds to be evaluated. Further, the pkCSM server was used to check for hepatotoxicity, *T. pyriformis* (log μg/L), and minnow (log mM) toxicity scores. *T. pyriformis* toxicity values were noted because it is considered as a toxic endpoint and minnow toxicity scores were relevant because this study involves compounds from marine sponges belonging to aquatic environments. Additionally, the StopTox server was used to investigate the acute toxicity caused by the compounds on exposure [84].

## 5. Conclusions

In conclusion, the in silico evaluation of target-specific therapeutic potential was aimed at narrowing the great selection of sponging metabolites to choose the best candidates for following pharmacological development [85,86]. Our results suggest that marine-sponge-based compounds might offer novel treatment against mucormycosis. In particular, the results in this in silico study indicated that naamine D, latrunculin A and S, (+)-curcudiol, (+)-curcuphenol, aurantoside I, and hyrtimomine A had better interactions with their respective targets compared to currently prescribed antifungal drugs, thus holding great antifungal potential that needs to be explored further. Only (+)-curcuphenol has shown some activity against all the seven therapeutic targets of this study. A combinatory approach may be required to treat the black fungi disease with significant improvement. Perhaps a precisely formulated cocktail of the different MNPs mentioned in this paper can prove beneficial in the efficient inhibition of mucormycosis. Our study has shown the methodology of how MNPs can be selected and investigated prior to their pharmaceutical development. The metabolites of other marine organisms should be screened in a similar way for antifungal and antiviral potencies against mucormycosis and SARS-CoV-2. It should be remembered that our work reveals only the pharmaceutical possibility of the screened compounds; laboratory interventions will be required to handle toxicity and enhance the drug-likeness before proceeding with in vitro and in vivo evaluations. 

## Figures and Tables

**Table 1:** Therapeutic target details and their significance.

Drug Targets			
Targets	NCBI Accession ID	Localization Probability	Function
RdRp (Region: RVT_1)	BAH03542.1	Extracellular	Replication of RNA
CotH3 (Region: CotH)	EIE87171	Extracellular	Protection
Lanosterol 14 alpha-demethylase	EIE87079	Plasma membrane	Ergosterol biosynthesis
Mucoricin	EIE81863	Cytoplasmic	Pathogenicity
	**PDB ID**		
Rhizopuspepsin	1UH9	Extracellular	Protein hydrolysis
Fungal Lipase	6A0W	Extracellular	Imparts virulence
exo-1,3-beta-glucan synthase	4M80	Extracellular	Beta-glucan production

(Localization probability estimated using CELLO2GO server.)

**Table 2:** The selection process of MNPs.

Databases Search	MNPs Selection for This Study			
PubChem and ChemSpider	MNPs Included	MNPs Excluded		
Marine Natural Products (MNPs)	Target Inhibitors	Target Non-Inhibitors	Pass Online (No Output)	Similar Output
Naamine A–G	A, B, D, E, F, G	C	-	-
Naamidine A–J	A, B, C	D-J	-	-
Hyrtimomine A–K	A, B, C, F, G	-	D, E, H, I, J, K	-
Xestodecalactone A–F	A–F	-	-	-
Topsentin, Topsentin A, C, D	Topsentin, A, D	C	-	-
Latrunculin A, B, C, D, M, S, T	A, B, S	C, D, M, T	-	-
Aurantoside A–K	A–K	-	-	A–H, J
(+)-Curcudiol, (+)-Curcuphenol	Yes	-	-	-
Tetillapyrone, Nortetillapyrone	Yes	-	-	-

**Table 3:** Bioactivity predictions for all chosen ligands.

PASS Online Predictions	
Ligands	Relevant Potential Biological Activities
Naamine A, B, D, E, F, G	Antifungal, anti-asthmatic, anti-infective, antiviral, mucositis treatment, a kinase inhibitor, a histidine kinase inhibitor, beta glucuronidase inhibitor, rhizopuspepsin RdRp inhibitor.
Naamidine A, B, C	Antifungal, anti-asthmatic, anti-infective, anti-eczematic, antiviral, a kinase inhibitor, histidine kinase inhibitor.
Hyrtimomine A, B, C, F, G	Antifungal, anti-infective, anti-inflammatory, mucositis treatment, a kinase inhibitor, a histidine kinase inhibitor, beta glucuronidase inhibitor, (hyrtimomine G-RdRp inhibitor, and rhizopuspepsin inhibitor).
Topsentin, Topsentin A, D	Antifungal, anti-infective, kinase inhibitor, mucositis treatment, histidine kinase inhibitor.
Latrunculin A, B, S	Antifungal, anti-eczematic, antiviral, anti-infective, antibiotic, respiratory distress relief syndrome treatment, antifungal enhancer, beta glucuronidase inhibitor, rhizopuspepsin inhibitor.
Xestodecalactone A, B, C, D, E, F	Antifungal, anti-eczematic, antiviral, anti-infective, a kinase inhibitor, a histidine kinase inhibitor, beta-glucuronidase inhibitor.
(+)-Curcudiol	Antifungal, anti-eczematic, antiviral, anti-infective, bronchodilator, mucositis treatment, antiseptic, histidine kinase inhibitor, a lipase inhibitor, exo-1,3-beta-glucan synthase inhibitor, RdRp inhibitor, lanosterol 14 alpha-demethylase inhibitor, alpha and beta-glucuronidase inhibitor.
(+)-Curcuphenol	Antifungal, anti-eczematic, antiviral, anti-infective, anti-inflammatory, bronchodilator, mucositis treatment, mucolytic, antiseptic, histidine kinase inhibitor, alpha and beta-glucuronidase inhibitor, lanosterol 14 alpha-demethylase inhibitor, a lipase inhibitor, exo-1, three beta-glucan-synthase inhibitors, RdRp inhibitor, rhizopuspepsin inhibitor.
Tetillapyrone	Antifungal, anti-eczematic, antiviral, anti-infective, histidine kinase inhibitor, beta-glucuronidase inhibitor, RdRp inhibitor, exo-1,3-beta-glucan-synthase inhibitors.
Nortetillapyrone	Antifungal, anti-eczematic, antiviral, anti-infective, mucolytic, histidine kinase inhibitor, alpha and beta-glucuronidase inhibitor, RdRp inhibitor, exo-1,3-beta-glucan-synthase inhibitors.
Aurantoside I, K	Antifungal, antiviral, antibiotic, anti-inflammatory, beta-glucuronidase inhibitor, exo-1,3-beta-glucan-synthase inhibitor.
**Drugs**	
Amphotericin B	Antifungal, antiviral, anti-inflammatory, anti-infective, antifungal enhancer, exo-1,3-beta-glucan-synthase inhibitor.
Isavuconazole	Antifungal, a kinase inhibitor, lanosterol 14 alpha-demethylase inhibitor.
Posaconazole	Antifungal, anti-eczematic, lanosterol 14 alpha-demethylase inhibitor.

(PASS online parameters—activity at pa > pi, pa > 0.3, and pa > 0.7; (pa) probability of being active).

**Table 4:** Ligands categorized based on their respective targets.

Targeted Biological Activity of Ligands		
CotH3	Mucoricin	exo-1,3-beta-glucan Synthase
Naamine A, B, E, F, G	Naamine A, B, E, F, G	(+)-Curcuphenol
Naamidine A, B, C	Hyrtimomine B, G	(+)-Curcudiol
Hyrtimomine A–C, F, G	Latrunculin A, B	Tetillapyrone
Topsentin	Xestodecalactone A–F	Nortetillapyrone
Topsentin A, D	Tetillapyrone	Aurantoside I, K
Xestodecalactone A–F	Nortetillapyrone	Amphotericin B (drug)
Tetillapyrone	(+)-Curcudiol	
Nortetillapyrone	(+)-Curcuphenol	
(+)-Curcudiol	Aurantoside I, K	
(+)-Curcuphenol		
Isavuconazole (drug)		
**RdRp**	**Rhizopuspepsin**	
Naamine D	Latrunculin S	
(+)-Curcudiol	Naamine D	
(+)-Curcuphenol	(+)-Curcuphenol	
Tetillapyrone	Hyrtimomine G	
Nortetillapyrone		
Hyrtimomine G		
**Fungal lipase**	**Lanosterol 14 alpha demethylase**	
(+)-Curcudiol	(+)-Curcudiol	
(+)-Curcuphenol	(+)-Curcuphenol	
	Isavuconazole (drug)	
	Posaconazole (drug)	

**Table 5:** Molecular docking output for CotH3-specific ligands.

CotH3					
Ligands	Binding Affinity (kcal/mol)	H Bond Residues	H Bonds	C-H Bonds	Hydrophobic Bonds
Naamine A	−6.9	ASN A: 237, PHE A: 235	2	2	3
Naamine B	−6.7	None	0	3	4
Naamine E	−6.8	SER A: 87, TRP A: 89, LEU A: 83, GLY A: 98	4	1	2
Naamine F	−6.7	PHE A: 235, ASN A: 23	2	0	5
Naamine G	−6.6	None	0	0	5
Naamidine A	−7.4	None	0	1	4
Naamidine B	−7.9	PHE A: 235	1	0	6
Naamidine C	−7.7	PHE A: 235, ASN A: 237	2	0	3
Hyrtimomine A	−9	None	0	0	4
Hyrtimomine B	−8	THR A: 241	1	0	4
Hyrtimomine C	−7.5	None	0	0	3
Hyrtimomine F	−8	THR A: 241	1	0	3
Hyrtimomine G	−7.5	LEU A: 143, THR A: 241	1	1	4
Topsentin	−8.1	LEU A: 143	1	0	5
Topsentin A	−8.8	PHE A: 235	1	1	3
Topsentin D	−8.2	None	0	0	6
Xestodecalactone A	−6.8	GLY A: 98, ASN A: 95	2	0	2
Xestodecalactone B	−6.6	ALA A: 91, GLY A: 98, ASP A: 84	3	0	1
Xestodecalactone C	−6.4	PHE A: 192	1	0	3
Xestodecalactone D	−6.7	LYS A: 109	1	0	2
Xestodecalactone E	−6.2	ALA A: 91, ASN A: 95	2	0	3
Xestodecalactone F	−6.2	THR A: 241	1	0	3
(+)-Curcudiol	−5.8	None	0	0	5
(+)-Curcuphenol	−6.5	None	0	0	4
Tetillapyrone	−6.1	ARG A: 6	1	0	1
Nortetillapyrone	−6.3	TYR A: 10	1	0	2
**Literature-Based Ligands Effective Against CotH3**					
12,28-Oxamanzamine A	−10.2	-	-	-	-
Haliclonacyclamine B	−9.2	-	-	-	-
Deoxytopsentin	−8.5	-	-	-	-
Vialinin B	−8.9	-	-	-	-
Olorofim	−8.6	-	-	-	-
**Drugs**					
Amphotericin B	−8.1	ASP A: 44, GLN A: 4, LEU A: 42	3	0	1
Isavuconazole	−8.0	ASN A: 237	1	0	4
Posaconazole	−8.8	LEU A: 193, THR A: 3	2	1	3

**Table 6:** Molecular docking output for mucoricin-specific ligands.

Mucoricin					
Ligands	Binding Affinity (kcal/mol)	H Bond Residues	H Bonds	C-H Bonds	Hydrophobic Bonds
Naamine A	−7.4	LEU A: 142, MET A: 1, GLU A: 97, PHE A: 3	4	0	1
Naamine B	−6.7	PHE A: 3	1	4	5
Naamine E	−7.6	LEU A: 142, PHE A: 3, GLU A: 141, GLU A: 97	4	0	2
Naamine F	−7.2	GLU A: 141, GLN A: 14, TRP A: 140	3	1	3
Naamine G	−7	PHE A: 3, GLU A: 97, LEU A: 142	3	1	2
Hyrtimomine B	−8.2	GLU A: 141, GLU A: 97	2	2	2
Hyrtimomine G	−7.6	PHE A: 3, ILE A: 95, MET A: 1, GLU A: 97	4	0	2
Latrunculin A	−8.6	GLU A: 97	1	0	3
Latrunculin B	−7.0	GLU A: 5	1	0	4
Xestodecalactone A	−6.6	GLU A: 97, MET A: 1	2	0	2
Xestodecalactone B	−6.9	MET A: 1	1	1	2
Xestodecalactone C	−7.5	LEU A: 142, GLU A: 97	2	0	1
Xestodecalactone D	−6.7	LEU A: 142	1	2	1
Xestodecalactone E	−6.3	LEU A: 142, MET A: 1, PHE A: 3	3	1	2
Xestodecalactone F	−6.1	ILE A: 95, MET A: 1	2	1	1
(+)-Curcudiol	−5.9	MET A: 1	1	0	2
(+)-Curcuphenol	−6.1	ILE A: 95	1	0	5
Tetillapyrone	−6.1	PHE A: 3, LEU A: 142, TRP A: 140	3	0	1
Nortetillapyrone	−6.2	GLN A: 14, PHE A:3	2	1	1
Aurantoside I	−6.8	ARG A: 130, SER A: 135, ASN A: 137	3	0	3
Aurantoside K	−7.1	PHE A: 3, GLU A: 5, ASN A: 52, GLU A: 97	4	0	4
**Literature-Based Ligands Effective against Mucoricin**					
Parsiguine	−8.2	-	-	-	-
Halicyclamine A	−8.2	-	-	-	-
Tetrahydrohaliclonacyclamine A	−8.2	-	-	-	-
Hesperidin	−8.0	-	-	-	-
12,28-Oxamanzamine A	−8.6	-	-	-	-
**Drugs**					
Amphotericin B	−6.8	GLN A: 76	1	0	1
Isavuconazole	−6.2	PHE A: 3, ILE A: 95	2	0	3
Posaconazole	−7.8	ARG A: 92, GLU A: 104, ASN A: 52, LEU A: 142, GLU A: 141	5	5	6

**Table 7:** Molecular docking output for exo-1,3-beta-glucan-synthase-specific ligands.

exo-1,3-beta-glucan Synthase					
Ligands	Binding Affinity (kcal/mol)	H Bond Residues	H Bonds	C-H Bonds	Hydrophobic Bonds
(+)-Curcudiol	−7.4	None	0	0	4
(+)-Curcuphenol	−8.0	GLU A: 185	1	0	4
Tetillapyrone	−7.8	ASN A: 139, TRP A: 356	2	0	2
Nortetillapyrone	−7.7	TYR A: 248, ASN A: 139, LEU A: 297	3	0	1
Aurantoside I	−11.4	GLN A:223, HIS A: 247, GLU A: 255, TYR A: 248, ARG A: 258, PHE A: 251	6	1	4
Aurantoside K	−8.9	THR A: 248, HIS A: 246, ASP A: 220, HIS A: 247	4	1	2
**Drugs**					
Amphotericin B	−9.4	HIS A: 246, PHE A: 225, ASP A: 273, ARG A: 258	4	0	0
Isavuconazole	−8.8	ASN A: 139, HIS A: 246, PHE A: 222, TRP A: 270	4	1	5
Posaconazole	−10.8	GLN A: 223, TYR A: 310	2	2	5

**Table 8:** Molecular docking output for RdRp-specific ligands.

RdRp					
Ligands	Binding Affinity (kcal/mol)	H Bond Residues	H Bonds	C-H Bonds	Hydrophobic Bonds
Naamine D	−8.8	PRO A: 39	1	0	4
(+)-Curcudiol	−6.1	PHE A: 133	1	0	3
(+)-Curcuphenol	−6.3	None	0	0	8
Tetillapyrone	−6.5	None	0	3	1
Nortetillapyrone	−6.1	HIS A: 50, SER A: 151	2	0	0
Hyrtimomine G	−7.1	GLU A: 35, SER A: 151, LEU A: 41, SER A: 40	4	0	1
**Literature-Based Ligands Effective against RdRp**					
Sofosbuvir (drug)	−6.1	-	-	-	-
Ribavirin (drug)	−6.6	-	-	-	-
**Drugs**					
Amphotericin B	−8.6	ARG A: 136, ASP A: 89, TRP A: 134	3	0	1
Isavuconazole	−7.8	PRO A: 10	1	0	4
Posaconazole	−8.2	TRP A: 134	1	2	4

**Table 9:** Molecular docking output for rhizopuspepsin-specific ligands.

Rhizopuspepsin					
Ligands	Binding Affinity (kcal/mol)	H Bond Residues	H Bonds	C-H Bonds	Hydrophobic Bonds
Naamine D	−6.3	ASN A: 13, VAL A: 277	2	1	3
Hyrtimomine G	−8.4	ASP A: 33, THR A: 222, ILE A: 15, ASP A: 79	4	1	2
Latrunculin S	−9.8	GLY A: 220	1	0	2
(+)-Curcuphenol	−6.7	ASP A: 218, ASP A: 35	2	0	5
**Drugs**					
Amphotericin B	−8.6	GLY A: 220	1	1	1
Isavuconazole	−6.4	SER A: 81, SER A: 113	2	0	3
Posaconazole	−8.7	ARG A: 192	1	3	2

**Table 10:** Molecular docking output for lanosterol 14 alpha-demethylase-specific ligands.

Lanosterol 14 Alpha-Demethylase					
Ligands	Binding Affinity (kcal/mol)	H Bond Residues	H Bonds	C-H Bonds	Hydrophobic Bonds
(+)-Curcudiol	−11.4	None	0	0	8
(+)-Curcuphenol	−9.2	LYS A: 466	1	0	3
**Literature-Based Ligands Effective against Lanosterol 14 Alpha Demethylase**					
Pramiconazole	−11.0	-	-	-	-
12,28-Oxamanzamine A	−10.9	-	-	-	-
Fascioquinol D	−10.8	-	-	-	-
Saperconazole	−10.8	-	-	-	-
Fascioquinol C	−10.4	-	-	-	-
**Drugs**					
Amphotericin B	44.2	ASP A: 176, ASN A: 503, PRO A: 501	3	1	2
Isavuconazole	−9.0	None	0	0	8
Posaconazole	−8.8	LYS A: 466, SER A: 147	2	1	6

**Table 11:** Molecular docking output for lipase-specific ligands.

Fungal Lipase					
Ligands	Binding Affinity (kcal/mol)	H Bond Residues	H Bonds	C-H Bonds	Hydrophobic Bonds
(+)-Curcudiol	−5.6	ASN A: 144	1	0	6
(+)-Curcuphenol	−8.0	ASN A: 144	1	0	5
**Drugs**					
Amphotericin B	−7.4	GLN A: 100, ASN A: 46, PRO A: 162, ASN A: 196, LYS A: 42	5	0	0
Isavuconazole	−6.7	ASP A: 217, VAL A: 216	2	2	3
Posaconazole	−7.8	GLN A: 100, TYR A: 78	2	4	4

**Table 12:** Summary of best-performing ligands and targets in this study.

Top-Ranking Complexes		
Targets	Ligands	Binding Affinity (Kcal/mol^−1^)
CotH3	Hyrtimomine A	−9.0
Mucoricin	Latrunculin A	−8.6
exo-1,3-beta-glucan synthase	Aurantoside I	−11.4
RdRp	Naamine D	−8.8
Rhizopuspepsin	Latrunculin S	−9.8
Lanosterol 14 alpha demethylase	(+)-Curcudiol	−11.4
Fungal lipase	(+)-Curcuphenol	−8.0

**Table 13:** Lipinski and additional parameters to examine drug-likeness of best ligands.

Drug-like Physicochemical Properties						
Ligands	Mol. Weight (g/mol)	Rotatable Bonds	H Bond Donors	H Bond Acceptors	C Log *p*	TPSA
	MW ≤ 500	RB ≤ 10	HBD ≤ 5	HBA ≤ 10	Log *p* ≤ 5	(Å^2^) ≤ 140
Hyrtimomine A	313.31	0	3	3	3.36	77.84
Latrunculin A	421.55	1	2	5	2.88	110.16
Aurantoside I	757.18	13	7	15	−0.53	257.23
Naamine D	323.39	6	2	3	3.05	73.16
Latrunculin S	423.57	3	3	5	2.88	121.16
(+)-Curcudiol	236.35	5	2	2	3.54	40.46
(+)-Curcuphenol	218.33	4	1	1	4.29	20.23
**Drugs**						
Amphotericin B	924.08	3	12	18	−0.39	319.61
Isavuconazole	437.47	6	1	7	3.82	115.86
Posaconazole	700.78	12	1	9	4.37	115.7

**Table 14:** Swiss-ADME selective parameters for best ligands.

Swiss-ADME Analysis				
Ligands	Water Solubility	Bioavailability	GI Absorption	BBB Permeant
Hyrtimomine A	Poor	0.55	High	No
Latrunculin A	Soluble	0.55	High	No
Aurantoside I	Soluble	0.11	Low	No
Naamine D	Poor	0.55	High	Yes
Latrunculin S	Soluble	0.55	High	No
(+)-Curcudiol	Moderate	0.55	High	Yes
(+)-Curcuphenol	Moderate	0.55	High	Yes
**Drugs**				
Amphotericin B	Soluble	0.17	Low	No
Isavuconazole	Poor	0.55	Low	No
Posaconazole	Poor	0.17	High	No

**Table 15:** OSIRIS output of potential harmful properties and druggability for best ligands.

OSIRIS							
Ligands	Irritant Potential	Mutagenic Potential	Tumorigenic Potential	Reproductive Effectivity	Drug Score	Drug Likeness	
	Risk Level					Score	Yes/No
Hyrtimomine A	Low risk	Low risk	Low risk	Low risk	0.40	0.79	Yes
Latrunculin A	Low risk	Low risk	Low risk	Low risk	0.29	−9.88	No
Aurantoside I	High risk	Low risk	Low risk	High risk	0.17	2.11	Yes
Naamine D	Low risk	Low risk	Low risk	Low risk	0.68	1.69	Yes
Latrunculin S	Low risk	Low risk	Low risk	Low risk	0.30	−9.72	No
(+)-Curcudiol	Low risk	Low risk	Low risk	Low risk	0.39	−5.06	No
(+)-Curcuphenol	High risk	Low risk	Low risk	Low risk	0.20	−5.62	No
**Drugs**							
Amphotericin B	Low risk	Low risk	Low risk	Low risk	0.27	−0.14	No
Isavuconazole	Low risk	Low risk	Low risk	Low risk	0.27	−3.87	No
Posaconazole	Low risk	High risk	High risk	Low risk	0.09	4.68	Yes

**Table 16:** Potential toxicity predictions using ProTox-II and pkCSM servers for best ligands.

	ProTox-II		PkCSM Toxicity Analysis		
Ligands	LD50 Value (mg/kg)	Toxicity Class	Hepatotoxicity	*T. pyriformis* (log μg/L)	Minnow (log mM)
Hyrtimomine A	400	4	Yes	0.285	0.191
Latrunculin A	560	4	Yes	0.313	2.123
Aurantoside I	5000	5	Yes	0.285	9.082
Naamine D	350	4	No	0.285	0.527
Latrunculin S	1000	4	Yes	0.295	2.304
(+)-Curcudiol	4000	5	No	1.468	0.175
(+)-Curcuphenol	1500	4	No	1.876	−0.277
**Drugs**					
Amphotericin B	100	3	No	0.285	11.261
Isavuconazole	1000	4	Yes	0.286	1.727
Posaconazole	320	4	Yes	0.285	−2.621

## Supplementary Materials

The following supporting information can be downloaded at: www.mdpi.com/article/10.3390/md20030215/s1, Figure S1: Energy minimized structures in YASARA; Figure S2: 3D structures of all Marine natural products; Figure S3: 3D structures of medical drugs; Table S1: List of ligands with respective SMILES line notations used in the study; Table S2: Ligands information and its chemical classification; Table S3: Molecular docking output for the target CotH3; Table S4: List of all interacting amino acids for CotH3; Table S5: Molecular docking output for the target Mucoricin; Table S6: List of all interacting amino acids for Mucoricin; Table S7: Molecular docking output for the target exo-1,3-beta-glucan synthase; Table S8: List of all interacting amino acids for fungal exo-1,3-beta-glucan synthase; Table S9: Molecular docking output for the target RdRp; Table S10: List of all interacting amino acids for RNA dependent/directed RNA polymerase; Table S11: Molecular docking output for the target Rhizopuspepsin; Table S12: List of all interacting amino acids for rhizopuspepsin; Table S13: Molecular docking output for the target lanosterol 14 alpha-demethylase; Table S14: List of all interacting amino acids for Lanosterol 14 alpha-demethylase; Table S15: Molecular docking output for the target lipase; Table S16: List of all interacting amino acids for fungal lipase; Table S17: Lipinski and additional parameters to examine drug-likeness; Table S18: Swiss-ADME selective parameters; Table S19: OSIRIS analysis for potential harmful properties and druggability; Table S20: Predictions for potential toxicity using ProTox-II and pkCSM servers; Table S21: StopTox acute toxicity analysis; Table S22: Molinspiration output for potential bioactivity.
