# Supplementary material for: In Silico Evaluation of Antifungal Compounds from Marine Sponges against COVID-19-Associated Mucormycosis
Source: Mar Drugs. 2022 Mar 20;20(3):215. doi: 10.3390/md20030215 (PMC8950821; doi:10.3390/md20030215)
Supplement: Supplementary file 1 [file marinedrugs-20-00215-s001.zip › marinedrugs-1575898-supplementary.pdf]

Supplementary Information Document

1. FASTA sequences: Protein targets

A. CotH3 (Cot H region):

```
>EIE87171.1:213-470 hypothetical protein RO3G_11882 [Rhizopus delemar RA 99-880]
DPTQIRERLYSDILHAMGTYANDATMVRLFINNQGFGTFNMLDDITQFSYINAKFYNGKPPATLGPLYDG
ASGADFLYHPGNLDGYSSWVANTANPNGEAYEALDPLCKAWNETTYTDNTAIANFEKMFDLDRFMRFMVI
EYLTADWDGYWMGQTNDGAYRDPTDNNKWYFLDQDFDGTFGVNLAAPEGNAFLDVSYKDFPSRYPGAVMI
NNLLQNADKKATFEKYLTETVRVLFNNVTLTNRVLALHNFLLPDLEWD
```

B. Mucoricin:

```
>EIE81863.1 hypothetical protein RO3G_06568 [Rhizopus delemar RA 99-880]
MYFEEGRLFFIKSQFNGRVLDVEDGSTEDDANIIVYTQKYEDCLNQLWRYENGYFINAKSAKVLDIRGGE
MQPESQIIQYAQKMVEEAANQRWAIDEDGYIFCEARPDLVLDIQGAEDEDCVPVILYERREGEVSANQRW
ELVPFEG
```

C. Exo-1,3-beta-glucan synthase:

```
>4M80_1|ChainA|EXO-1,3-BETA-GLUCANASE|Candidaalbicans
GGHNVAWDYDNNVIRGVNLGGWFVLEPYMTPSLFEPFQNGNDQSGVPVDEYHWTQTLGKEAALRILQKHWSTWITEQDFKQISNLGL
NFVRIPIGYWAFQLLDNDPYVQGQVQYLEKALGWARKNNIRVWIDLHGAPGSQNGFDNSGLRDSYNFQNGDNTQVTLNVLNTIFKKY
GGNEYSDVVIGIELLNEPLGPVLNMDKLKQFFLDGYNSLRQTGSVTPVIIHDAFQVFGYWNNFLTVAEGQWNVVVDHHHYQVFSGGE
LSRNINDHISVACNWGWDAKKESHWNVAGSWSAALTDCAKWLNGVNRGARYEGAYDNAPYIGSCQPLLDISQWSDEHKTDTRRYIEA
QLDAFEYTGGWVFWSWKTENAPEWSFQTLTYNGLFPQPVTDRQFPNQCGFH
```

D. RDRP (RVT_1 region):

```
>BAH03542.1:547-807 polyprotein [Rhizopus arrhizus]
IYKKGDPLQPANYRPISLTSVLRKLFELCLQTTLEDTAPSLDPVQGGFRHSRSALDQALCLNELCRQHAI
DHHGEPPVLAFLDIKSAYDTVDRAIIWRALETYISPALLGLLQCLFDKVSIEVLVSGFTSPAFWPRTGVL
QGSILSPFLYSIYINSLPALLRSVRLPISARYYSSNPQREFDGLWLNCLLYADDVVLIGAPEVMPRLLKA
AEEHSFSLGYRWNPAKCVVLNSAFSLGGPQLKLYGDDIPIQSTFNYLGIPF
```

E. Rhizopuspepsin:

```
>1UH9_1|Chain A|rhizopuspepsin I|Rhizopus microsporus var. chinensis
AGVGTVPMTDYGNDIEYYGQVTIGTPGKKFNLDFDTGSSDLWIASTLCTNCGSRQTKYDPNQSSTYQADGRTWSISYGDGSSASGIL
AKDNVNLGGLLIKGQTIELAKREAASFASGPNDGLLGLGFDTITTVRGVKTPMDNLISQGLISRPIFGVYLGKAKNGGGGEYIFGGY
DSTKFKGSLTTVPIDNSRGWWGITVDRATVGTSTVASSFDGILDTGTTLLILPNNIAASVARAYGASDNGDGTYTISCDTSRFKPLV
FSINGASFQVSPDSLVFEEFQGQCIAGFGYGNWDFAIIGDTFLKNNYVVFNQGVPEVQIAPVAE
```

F. Lanosterol 14-alpha demethylase:

```
>EIE87079.1 lanosterol 14-alpha demethylase [Rhizopus delemar RA 99-880]
MAVISTLLPTLESIPLYAVLALGVFVIINILSQWFGPKNPKEPPVVFSWIPFMGNAIEFGINPIAFLQKC
QKKYGDVFTFYMVGKRVTVFLNADGNQFVFNAKQNLTSAADAYNHMTKHVFGPEVVYDAPHSVFMEQKRF
IKAGLNSESFRQHVPMIVEEVEGFFKNYKKPTGAFDAYHTLGELIICTASRCLMGKEIRASLDDSVAGLY
YDLDQGFKPINFIFPNLPLPSYRKRDVARQKMTDLYSSIIARRKAENDFSNADLLQALMDANYKDGSNVP
DHHIAGMMIAVLFGGQHTSATTSAWTLLELAARPDLIRDLREEQITKLGSLKADLTFDNLKELTLLDSCV
RETLRLHPPIFQMMRRVTANKVVFEKTGHEIPKGNFLCAVPGVTQVDSQYFNEPLKYDPLRWINLTDPVH
SMEAGDDSNIDYGFGAVGISSKNPFLPFGAGRHRCIGEQFGYLQIKTIIATIIRLFDIELEDGKGVPKSD
YTSMVVVPERPSNIKYTWRE
```

G. Lipase:

```
>6A0W_1|Chain A|Lipase|Rhizopus chinensis
DTETVGGMTLDLPENPPPIPATSTAPSSDSGEVVTATAAQIKELTNYAGVAATAYCRSVVPGTKWDCKQCLKYVPDGKLIKTFTSLL
TDTNGFILRSDAQKTIYVTFRGTNSFRSAITDMVFTFTDYSPVKGAKVHAGFLSSYNQVVKDYFPVVQDQLTAYPDYKVIVTGHSLG
GAQALLAGMDLYQREKRLSPKNLSIYTVGCPRVGNNAFAYYVDSTGIPFHRTVHKRDIVPHVPPQAFGYLHPGVESWIKEDPADVQI
CTSNIETKQCSNSIVPFTSIADHLTYFGINEGSCLGSSHHHHHH
```

Table S1: List of ligands with respective SMILES line notations used in the study

Ligands	SMILES
Naamine A	CN1C(=C(N=C1N)CC2=CC=C(C=C2)OC)CC3=CC=C(C=C3)O
Naamine B	CN1C(=C(N(C1=N)C)CC2=CC(=C(C=C2)OC)O)CC3=CC=C(C=C3)OC
Naamine D	COC1=CC=C(C=C1)CC2=C(N=C(N2)N)CC3=CC=C(C=C3)OC
Naamine E	CN1C(=C(N=C1N)CC2=CC=C(C=C2)OC)CC3=CC(=C(C(=C3)O)OC)O
Naamine F	CN1C(=C(N=C1N)CC2=CC=C(C=C2)OC)CC3=CC(=C(C=C3)O)OC
Naamine G	CN1C(=C(N=C1N)CC2=CC=C(C=C2)OC)CC3=CC(=C(C(=C3)OC)O)OC
Naamidine A	CN1C(=C(N=C1N=C2C(=O)N(C(=O)N2)C)CC3=CC=C(C=C3)OC)CC4=CC=C(C=C4)O
Naamidine B	CN1C(=C(N=C1N=C2C(=O)N(C(=O)N2)C)CC3=CC=C(C=C3)OC)CC4=CC(=C(C=C4)OC)O
*Naamidine C	CN1C(C(NC1N=C2C(=O)N(C(=O)N2C)C)CC3CCC(CC3)OC)CC4CCC(CC4)O
Hyrtimomine A	C1=CC2=C(C=C1O)C3=C(N2)OC4=C5C3=NC=CC6=CNC(=C65)C=C4
Hyrtimomine B	C1C(NC2=C3C(=CC=C4C3=C1C=N4)OC5=C2C6=C(N5)C=CC(=C6)O)C(=O)O
Hyrtimomine C	C1C(=O)C2=CNC3=C2C(=C(C=C3)O)C(=N1)C4=CNC5=C4C=C(C=C5)O
Hyrtimomine F	C1=CC2=C(C=C1O)C(=CN2)C(=O)C3C4=C(C=CC5=C4C(=CN5)C(=O)C(=O)N3)O
Hyrtimomine G	C1=CC2=C(C=C1O)C(=CN2)C(=O)C(C(C(=O)C3=CNC4=C3C=C(C=C4)O)O)O
Topsentin	C1=CC=C2C(=C1)C(=CN2)C3=CN=C(N3)C(=O)C4=CNC5=C4C=CC(=C5)O
Topsentin A	C1=CC=C2C(=C1)C(=CN2)C3=CN=C(N3)C(=O)C4=CNC5=CC=CC=C54
Topsentin D	C1C(NC(=N1)C(=O)C2=CNC3=CC=CC=C32)C4=CNC5=CC=CC=C54
Latrunculin A	CC1CCC2CC(CC(O2)(C3CSC(=O)N3)O)OC(=O)C=C(CCC=CC=C1)C
Latrunculin B	CC1CCC2CC(CC(O2)(C3CSC(=O)N3)O)OC(=O)C=C(CCC=C1)C
Latrunculin S	CC1CCC(CC(OC(=O)C=C(CCC=CC=C1)C)CC(C2CSC(=O)N2)O)O
Xestodecalactone A	CC1CCCC(=O)C2=C(CC(=O)O1)C=C(C=C2O)O
Xestodecalactone B	CC1CC(CC(=O)C2=C(CC(=O)O1)C=C(C=C2O)O)O
Xestodecalactone C	CC1CC(CC(=O)C2=C(CC(=O)O1)C=C(C=C2O)O)O
Xestodecalactone D	CC1CC(CC(=O)C2=C(C(=C(C=C2CC(=O)O1)O)OC)O)O
Xestodecalactone E	CCCCOC1CC(OC(=O)CC2=CC(=C(C(=C2C(=O)C1)O)OC)O)C
Xestodecalactone F	CCCCOC1CC(OC(=O)CC2=CC(=C(C(=C2C=C1)O)OC)O)C
(+)-Curcudiol	CC1=CC(=C(C=C1)C(C)CCCC(C)(C)O)O
(+)-Curcuphenol	CC1=CC(=C(C=C1)C(C)CCC=C(C)C)O
*Tetillapyrone	CC1CC(C(OC1=O)O)[C@@H]2C[C@H]([C@@H](O2)CO)O
*Nortetillapyrone	C1CC(OC(=O)C1[C@@H]2C[C@H]([C@@H](O2)CO)O)O
Aurantoside I	CC1C(C(C(O1)OC2COC(C(C2O)O)OC3C(C(COC3N4C(C(=O)C(=C(C=CC=CC=CC=CC=C(C)Cl)O)C4=O)CC(=O)N)O)O)OC)O

Aurantoside K	CC1C(C(C(O1)OC2COC(C(C2O)O)OC3C(C(COC3N4C(C(=O)C(=C(C=CC=CC=CC=CC=C(C)Cl)O)C4=O)CC(=O)N)O)O)O)O
**Drugs**	
Amphotericin B	CC1C=CC=CC=CC=CC=CC=CC=CC(CC2C(C(CC(O2)(CC(CC(C(CCC(CC(CC(=O)OC(C(C1O)C)C)O)O)O)O)O)O)O)C(=O)O)OC3C(C(C(C(O3)C)O)N)O
Isavuconazole	CC(C1=NC(=CS1)C2=CC=C(C=C2)C#N)C(CN3C=NC=N3)(C4=C(C=CC(=C4)F)F)O
Posaconazole	CCC(C©O)N1C(=O)N(C=N1)C2=CC=C(C=C2)N3CCN(CC3)C4=CC=C(C=C4)OCC5CC(OC5)(CN6C=NC=N6)C7=C(C=C(C=C7)F)F

(*Naamidine C, Tetillapyrone and Nortetillapyrone – Isomeric SMILES from Chemspider database)

Table S2: Ligands information and its chemical classification

Ligand Information			
**Ligands**	**Formulae**	**Pubchem CID**	**Class**
Naamine A	$C_{19}H_{21}N_3O_2$	10019087	**Alkaloids**
Naamine B	$C_{21}H_{25}N_3O_3$	11233956	
Naamine D	$C_{19}H_{21}N_3O_2$	482905	
Naamine E	$C_{20}H_{23}N_3O_4$	10970661	
Naamine F	$C_{20}H_{23}N_3O_3$	21578946	
Naamine G	$C_{21}H_{25}N_3O_4$	11153300	
Naamidine A	$C_{23}H_{23}N_5O_4$	135455949	
Naamidine B	$C_{24}H_{25}N_5O_5$	135511072	
*Naamidine C	$C_{24}H_{25}N_5O_4$	10313314	
Hyrtimomine A	$C_{19}H_{11}N_3O_2$	71681276	
Hyrtimomine B	$C_{20}H_{13}N_3O_4$	136267825	
Hyrtimomine C	$C_{19}H_{13}N_3O_3$	136267826	
Hyrtimomine F	$C_{20}H_{13}N_3O_5$	73774545	
Hyrtimomine G	$C_{20}H_{16}N_2O_6$	73774544	
Topsentin	$C_{20}H_{14}N_4O_2$	72457	
Topsentin A	$C_{20}H_{14}N_4O$	183527	
Topsentin D	$C_{20}H_{16}N_4O$	12018820	
Latrunculin A	$C_{22}H_{31}NO_5S$	445420	**Macrolides**
Latrunculin B	$C_{20}H_{29}NO_5S$	6436219	
Latrunculin S	$C_{22}H_{33}NO_5S$	10093792	
Xestodecalactone A	$C_{14}H_{16}O_5$	637028	**Bioactive Metabolites**
Xestodecalactone B	$C_{14}H_{16}O_6$	9943580	
Xestodecalactone C	$C_{14}H_{16}O_6$	11033245	
Xestodecalactone D	$C_{15}H_{18}O_7$	60154235	
Xestodecalactone E	$C_{19}H_{26}O_7$	60154236	
Xestodecalactone F	$C_{19}H_{26}O_6$	139584779	
(+)-Curcudiol	$C_{15}H_{24}O_2$	184024	**Sesquiterpene phenols**
(+)-Curcuphenol	$C_{15}H_{22}O$	156118	
*Tetillapyrone	$C_{11}H_{14}O_6$	10213954	**Hydroxypyran-2-ones**
*Nortetillapyrone	$C_{10}H_{12}O_6$	10213956	
Aurantoside I	$C_{34}H_{45}ClN_2O_{15}$	54723407	**Tetramic acid glycosides**
Aurantoside K	$C_{33}H_{43}ClN_2O_{15}$	86575300	
		**Chemspider ID**	
Amphotericin B	$C_{47}H_{73}NO_{17}$	10237579	**Drugs**
Isavuconazole	$C_{22}H_{17}F_2N_5OS$	5293682	
Posaconazole	$C_{37}H_{42}F_2N_8O_4$	411709	

(*Naamidine C, Tetillapyrone, and nortetillapyrone obtained from Chemspider Database)

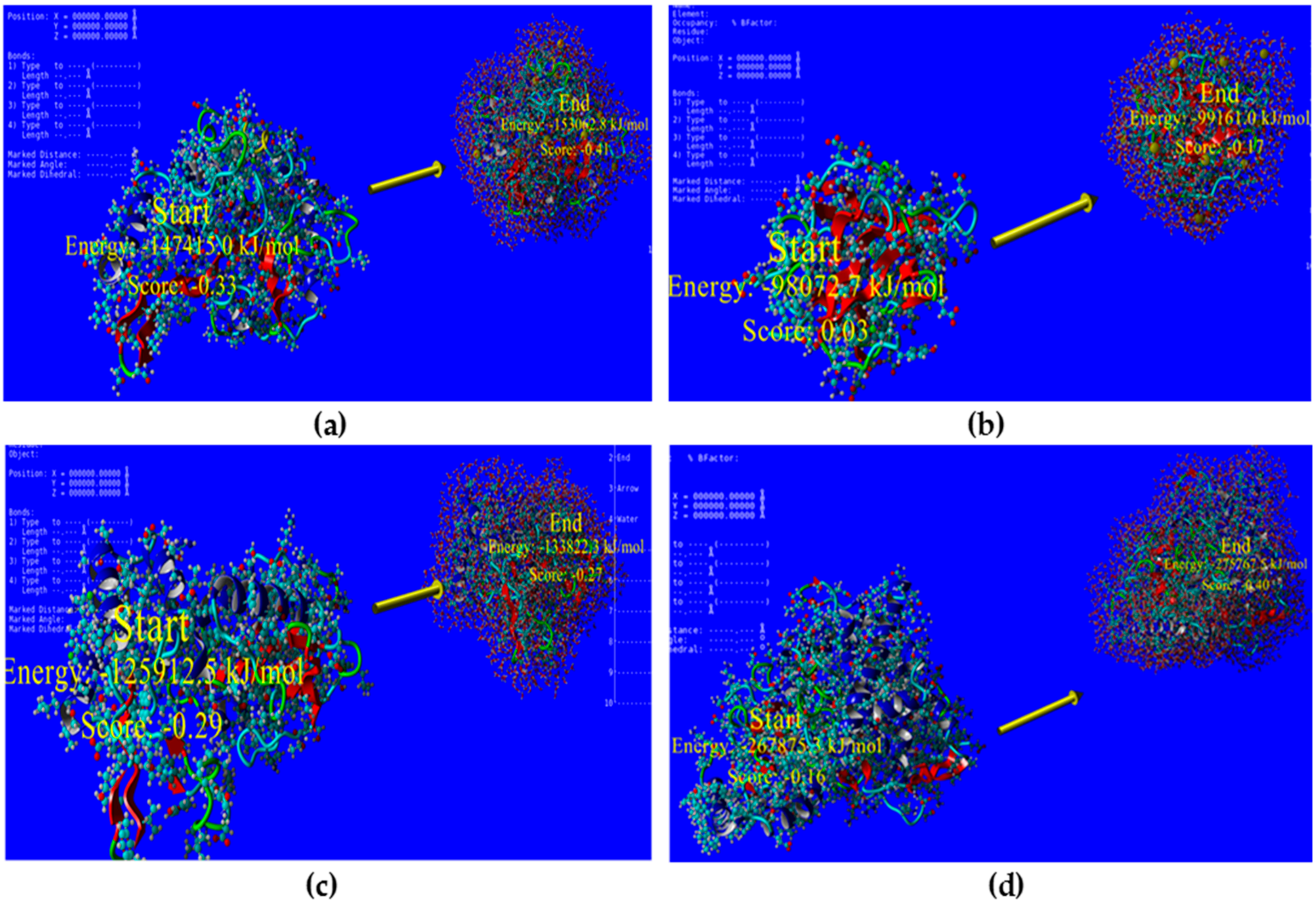

Figure S1. Energy minimized structures in YASARA (a) CotH3; (b) Mucoricin; (c) RdRp; (d) Lanosterol 14 alpha-demethylase.

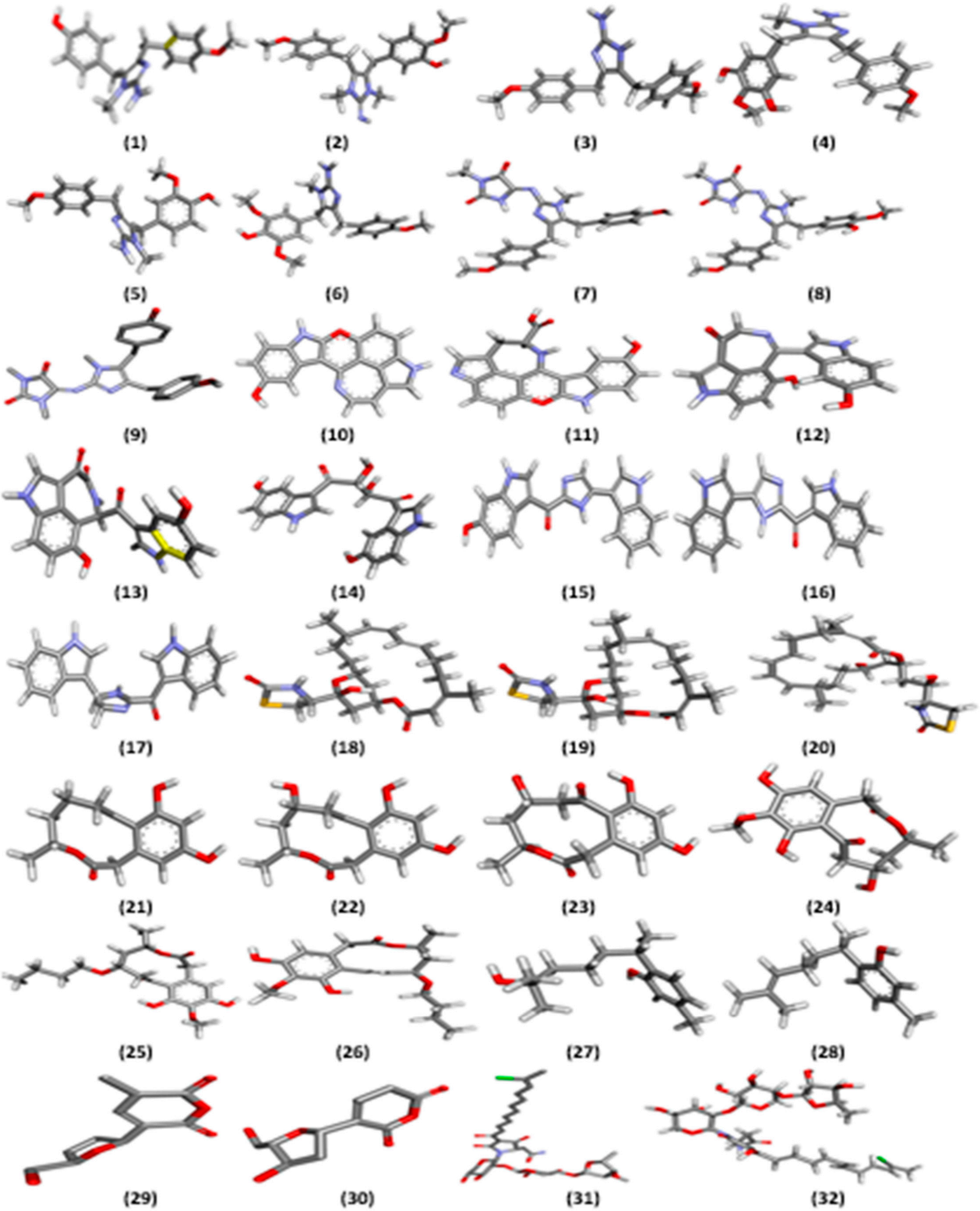

Figure S2. MNPs 3D structures (1) Naamine A, (2) Naamine B, (3) Naamine D, (4) Naamine E, (5) Naamine F, (6) Naamine G, (7) Naamidine A, (8) Naamidine B, (9) Naamidine C, (10) Hyrtimomine A, (11) Hyrtimomine B, (12) Hyrtimomine C, (13) Hyrtimomine F, (14) Hyrtimomine G, (15) Topsentin, (16) Topsentin A, (17) Topsentin D, (18) Latrunculin A, (19) Latrunculin B, (20) Latrunculin S, (21) Xestodecalactone A, (22) Xestodecalactone B, (23) Xestodecalactone C, (24) Xestodecalactone D, (25) Xestodecalactone E, (26) Xestodecalactone F, (27) (+)-Curcudiol, (28) (+)-Curcuphenol, (29) Tetillapyrone, (30) Nortetillapyrone, (31) Aurantoside I, (32) Aurantoside K.

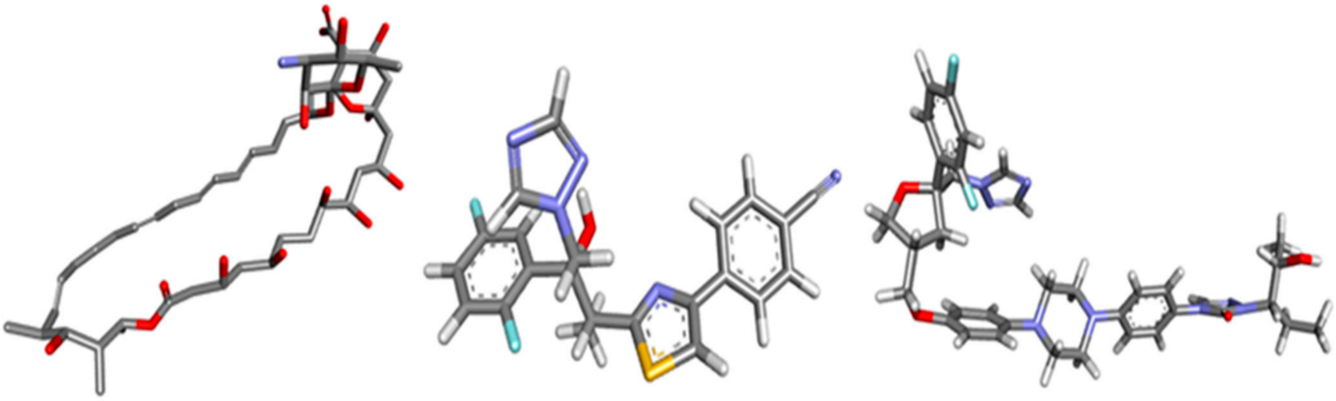

(a) (b) (c)

Figure S3: 3D structures of medical drugs (a) Amphotericin B, (b) Isavuconazole, (c) Posaconazole.

Table S3: Molecular docking output for the target CotH3

CotH3					
**Ligands**	**Binding affinity (Kcal/mol-1)**	**H bond residues**	**H bonds**	**C-H bonds**	**Hydrophobic bonds**
Naamine D	-6.7	ASN A: 237, PHE A: 235	2	0	5
Latrunculin A	-7.1	ASN A: 237	1	0	2
Latrunculin B	-7.2	None	0	1	5
Latrunculin S	-6.7	ALA A: 91, SER A: 87	2	0	0
Aurantoside I	-7.3	TRP A: 147, ASP A: 175, ASP A: 177, GLU A: 7, ASN A: 40	5	1	2
Aurantoside K	-7.9	GLN A: 4, THR A: 38, THR A: 3, PRO A: 2, HIS A:248	5	1	6

Table S4: List of all interacting amino acids for CotH3

CotH3	
**Ligands**	**Interacting residues**
Naamine A	ASN A: 237, PHE A: 235, PHE A: 180, LEU A: 240, LEU A: 9
Naamine B	LEU A: 9, PHE A: 180, LEU A: 240, LEU A: 193, THR A: 179, ARG A: 6, LEU A: 143
Naamine D	ASN A: 237, PHE A: 235, LEU A: 240, LEU A: 9, PHE A: 180, LEU A: 143, LEU A: 193
Naamine E	SER A: 87, TRP A: 89, LEU A: 83, GLY A: 98, ALA A: 100, ALA A: 91, ASN A: 95
Naamine F	PHE A: 235, ASN A: 237, ARG A: 6, LEU A: 9, VAL A: 244, PHE A: 180, LEU A: 240
Naamine G	LEU A: 240, PHE A: 180, LEU A: 9, THR A: 241, VAL A: 244
Naamidine A	LEU A: 240, PHE A: 180, VAL A: 244, ARG A: 6, THR A: 3, THR A: 144
Naamidine B	PHE A: 235, LEU A: 193, VAL A: 231, PHE A: 180, ARG A: 6, LEU A: 9, LEU A: 240
Naamidine C	PHE A: 235, ASN A: 237, LEU A: 240, PHE A: 180, VAL A: 244
Hyrtimomine A	LEU A: 9, VAL A: 244, ARG A: 6, LEU A: 240
Hyrtimomine B	THR A: 241, LEU A: 9, PHE A: 180, ARG A: 6, VAL A: 244
Hyrtimomine C	LEU A: 143, LEU A: 227, TYR A: 197
Hyrtimomine F	THR A: 241, PHE A: 180, LEU A: 240, VAL A: 244
Hyrtimomine G	LEU A: 143, THR A: 241, VAL A: 231, VAL A: 244, LEU A: 240, PHE A: 180, THR A:144
Topsentin	LEU A: 143, VAL A: 231, LEU A: 240, PHE A: 180, LEU A: 9, ARG A: 6,
Topsentin A	PHE A: 235,PHE A: 110, LEU A: 240, LEU A: 9, ARG A: 6
Topsentin D	PHE A: 180, LEU A: 240, LEU A: 143, LEU A: 9, VAL A: 244, PHE A: 235
Latrunculin A	ASN A: 237, LEU A: 240, LEU A: 9
Latrunculin B	VAL A: 231, LEU A: 143, PHE A: 235, PHE A: 200, LEU A: 193, THR A: 228
Latrunculin S	ALA A: 91, SER A: 87

Xestodecalactone A	GLY A: 98, ASN A: 95, ALA A: 91, ALA A: 100
Xestodecalactone B	ALA A: 91, GLY A: 98, ASP A: 84, ALA A: 100
Xestodecalactone C	PHE A: 192, VAL A: 231, LEU A: 143, PHE A: 235
Xestodecalactone D	LYS A: 109, PRO A: 106, ALA A: 110
Xestodecalactone E	ALA A: 91, ASN A: 95, VAL A: 90, TYR A: 101, ALA A: 100
Xestodecalactone F	THR A: 241, VAL A: 244, PHE A: 180, LEU A: 240
(+)-Curcudiol	PHE A: 235, PHE A: 180, LEU A: 240, LEU A: 9, ARG A: 6
(+)-Curcuphenol	PHE A: 480, LEU A: 240, LEU A: 9, ARG A: 6
Tetillapyrone	ARG A: 6
Nortetillapyrone	TYR A: 10, VAL A: 244, PHE A: 180
Aurantoside K	GLN A: 4, THR A: 38, THR A: 3, PRO A: 2, HIS A:248, ASP A: 258, LEU A: 240, PHE A: 235, VAL A: 244, LEU A: 9, PHE A: 180, ARG A: 6
Aurantoside I	TRP A: 147, ASP A: 175, ASP A: 177, GLU A: 7, ASN A: 40, ASP A: 148, ASP A: 146, GLY A: 178
**Drugs**	
Amphotericin B	ASP A : 44, GLN A: 4, LEU A: 42, ASP A: 43
Isavuconazole	ASN A: 237, LEU A: 143, PHE A: 180, LEU A: 240, LEU A: 193
Posaconazole	LEU A: 193, THR A: 3, PHE A: 180, LEU A: 240, ARG A: 6, LEU A: 9

Table S5: Molecular docking output for the target Mucoricin

**Mucoricin**					
**Ligands**	**Binding affinity (Kcal/mol-1)**	**H bond residues**	**H bonds**	**C-H bonds**	**Hydrophobic bonds**
Naamine D	-7.1	GLU A: 97, PHE A: 3	2	0	3
Naamidine A	-7.9	PHE A: 3, ARG A: 139, GLN A: 14	3	2	5
Naamidine B	-7.8	ARG A: 139, GLN A: 14	2	2	6
Naamidine C	-8.0	GLN A: 14, TRP A: 140	2	1	7
Hyrtimomine A	-7.5	GLU A: 97	1	1	3
Hyrtimomine C	-7.2	ASP A: 96, ARG A: 139, GLN A: 14, GLU : 97	4	1	2
Hyrtimomine F	-7.6	ILE A: 113, ARG A: 130, GLU A: 133, GLU A: 74	4	2	1
Topsentin	-8.4	LEU A: 142, GLU A: 97	2	1	3
Topsentin A	-8.3	GLY A: 97, LEU A: 142	2	1	3
Topsentin D	-8.2	None	0	2	4
Latrunculin S	-6.2	ALA A: 116, ILE A: 113	2	0	1

Table S6: List of all interacting amino acids for Mucoricin

Mucoricin	
**Ligands**	**Interacting residues**
Naamine A	LEU A: 142, MET A: 1, GLU A: 97, PHE A: 3, ILE A: 95
Naamine B	PHE A: 3, MET A: 1, ILE A: 95, LEU A: 142, VAL A: 143, TYR A: 2, GLU A: 141, TRP A: 140, GLN A: 14, GLU A: 97
Naamine D	GLU A: 97, PHE A: 3, ILE A: 95, MET A: 1, VAL A: 143
Naamine E	LEU A: 142, PHE A: 3, GLU A: 141, GLU A: 97, MET A: 1, ILE A: 95
Naamine F	GLU A: 141, GLN A: 14, TRP A: 140, MET A: 1, LEU A: 142, ILE A: 95, TYR A: 2
Naamine G	PHE A: 3, GLU A: 97, LEU A: 142, TYR A: 2, ILE A: 95, MET A: 1
Naamidine A	PHE A: 3, ARG A: 139, GLN A: 14, GLU A: 97, TRP A: 140, GLU A: 141, MET A: 1, VAL A: 143, LEU A: 142, ILE A: 95
Naamidine B	ARG A: 139, GLN A: 14, GLU A: 141, MET A: 1, ILE A: 95, VAL A: 143, LEU A: 142, TYR A: 2, TRP A: 140, GLU A: 97
Naamidine C	GLN A: 14, TRP A: 140, GLU A: 97, ARG A: 139, GLU A: 141, MET A: 1, ILEA: 95, VAL A: 143, LEU A: 142, TYR A: 2
Hyrtimomine A	GLU A: 97, LEU A: 142, ILE A: 95, TYR A: 2, MET A: 1
Hyrtimomine B	GLU A: 141, GLU A: 97, VAL A: 143, LEU A: 142, ILE A: 95, MET A: 1
Hyrtimomine C	ASP A: 96, ARG A: 139, GLN A: 14, GLU : 97, LEU A: 142, ILE A: 92, GLU A: 141
Hyrtimomine F	ILE A: 113, ARG A: 130, GLU A: 133, GLU A: 74, ASP A: 112, SER A: 135, ALA A: 116
Hyrtimomine G	PHE A: 3, ILE A: 95, MET A: 1, GLU A: 97, ALA A: 94, TYR A: 2
Topsentin	LEU A: 142, GLU A: 97, TYR A: 2, ILE A: 95, GLU A: 141, MET A: 1
Topsentin A	GLY A: 97, LEU A: 142, ASP A: 96, TYR A: 2, ILE A: 95, GLU A: 141
Topsentin D	ILE A: 95, TYR A: 2, MET A: 1, GLU A: 141, GLU A: 97, ASP A: 96
Latrunculin A	GLU A: 97, TYR A: 2, ILE A: 95, LEU A: 142
Latrunculin B	GLU A:: 5, TYR A: 50, PHE A: 3, ILE A: 95, LEU A: 142
Latrunculin S	ALA A: 116, ILE A: 113, ASP A: 112
Xestodecalactone A	GLU A: 97, MET A: 1, LEU A: 142, ILE A: 95
Xestodecalactone B	MET A: 1, GLY A: 99, ILE A: 95, LEU A: 142
Xestodecalactone C	LEU A: 142,GLU A: 97, ILE A: 95
Xestodecalactone D	LEU A: 142, MET A: 1, GLU A: 141, ILE A: 95
Xestodecalactone E	LEU A: 142, MET A: 1, PHE A: 3, VAL A: 143, ILE A: 95, GLU A: 141
Xestodecalactone F	ILE A: 95, MET A: 1, TYR A: 2, GLU A: 97
(+)-Curcudiol	MET A: 1, ILE A: 95, TYR A: 2
(+)-Curcuphenol	ILE A: 95, TYR A: 2, TYR A: 50, LEU A: 142, PHE A: 2, PHE A: 50
Tetillapyrone	PHE A: 3, LEU A: 142, TRP A: 140, ILE A: 95
Nortetillapyrone	GLN A: 14, PHE A:3, GLY A: 99, GLU A: 97
Aurantoside K	PHE A: 3, GLU A: 5, ASN A: 52, GLU A: 97, VAL A: 143, LEU A: 142, ILE A: 95, MET A: 1
Aurantoside I	ARG A: 130, SER A: 135, ASN A: 137, ILE A: 125, TYR A: 127, GLU A: 133
**Drugs**	
Amphotericin B	GLN A: 76, ARG A: 67
Isavuconazole	PHE A: 3, ILE A: 95, ALA A: 94, PHE A: 102, TYR A: 2
Posaconazole	ARG A: 92, GLU A: 104, ASN A: 52, LEU A: 142, GLU A: 141, GLU A: 5, ILE A: 95, TYR A: 50, ASP A: 96, MET A: 1, ALA A: 94, TYR A: 2, GLU A: 97, TRP A: 140, ASP A: 98, GLN A: 14

Table S7: Molecular docking output for the target Exo-1, 3 beta-glucan synthase

Exo-1,3 beta-glucan synthase					
**Ligands**	**Binding affinity (Kcal/mol-1)**	**H bond residues**	**H bonds**	**C-H bonds**	**Hydrophobic bonds**
Naamine A	-9.2	ASP A: 138	1	1	5
Naamine B	-9.1	ARG A: 305, ASN A: 298, ASP A: 138, LEU A: 297	4	2	4
Naamine D	-9.2	ARG A: 305, SER A: 285, ASP A: 138	3	2	5
Naamine E	-9.3	ASN A: 298, TYR A: 248, GLU A: 185	3	0	3
Naamine F	-9.3	ARG A: 305, ASP A: 338, SER A: 285	3	3	5
Naamine G	-8.8	LEU A: 297, ARG A: 305	2	2	6
Naamidine A	-10.6	HIS A: 128, LEU A: 297, ASP A: 138	3	2	4
Naamidine B	-9.7	GLU A: 185, ASN A: 298, ARG A: 302	3	0	6
Naamidine C	-10.7	TYR A: 22, SER A: 285, TYR A: 248, ARG A: 305	4	2	5
Hyrtimomine A	-11.2	GLU A: 185, SER A: 285	2	0	3
Hyrtimomine B	-11.0	TYR A: 248, GLU A: 185	2	0	3
Hyrtimomine C	-10.6	ASN A: 139	1	0	7
Hyrtimomine F	-9.9	ASN A: 139, LEU A: 297, GLY A: 136	3	0	4
Hyrtimomine G	-10.0	GLU A: 185, TYR A: 22, GLU A: 20, ASN A: 139, ASP A: 138	5	1	4
Topsentin	-10.3	LEU A: 297, ASP A: 138, ARG A: 305, GLU A: 185	4	2	2
Topsentin A	-10.1	ASP A: 138, LEU A: 297, GLU A: 185	3	2	2
Topsentin D	-10.1	LEU A: 297, GLU A: 185, GLY A: 136, PHE A: 137	4	0	2
Latrunculin A	-8.9	None	0	0	2
Latrunculin B	-8.8	TYR A: 310	1	1	2
Latrunculin S	-8.3	ARG A: 258	1	0	2
Xestodecalactone A	-8.4	ASN A: 139, ARG A: 305	2	0	3
Xestodecalactone B	-8.3	ASN A: 139, LEU A: 297	2	0	1
Xestodecalactone C	-8.4	ASN A: 139	1	0	3
Xestodecalactone D	-8.7	ASN A: 139, LEU A: 297, ARG A: 305	3	1	3
Xestodecalactone E	-8.0	ASP A: 144, ASP A: 138	2	2	3
Xestodecalactone F	-8.9	ASP A: 138, ASN A: 139	2	2	3

Table S8: List of all interacting amino acids for fungal exo-1, 3-beta-glucan synthase

Exo-1,3-beta-glucan synthase	
**Ligands**	**Interacting residues**
Naamine A	ASP A: 138, GLU A: 185, PHE A: 137, PHE A: 251, HIS A: 266, HIS A: 128, TRP A: 356
Naamine B	ARG A: 305, ASN A: 298, ASP A: 138, LEU A: 297, ARG A: 184, GLU A: 185, PHE A: 251, TRP A: 356, HIS A: 128, HIS A: 246
Naamine D	ARG A: 305, SER A: 285, ASP A: 138, PHE A: 137, GLU A: 20, TRP A: 356, HIS A: 246, HIS A: 128, PHE A: 251, GLU A: 185
Naamine E	ASN A: 298, TYR A: 248, GLU A: 185, PHE A: 222, PHE A: 137, PHE A: 251
Naamine F	ARG A: 305, ASP A: 338, SER A: 285, ASN A: 298, TYR A: 310, GLU A: 185, PHE A: 251, PHE A: 137, TRP A: 356, HIS A: 128, HIS A: 246
Naamine G	LEU A: 297, ARG A: 305, ASN A: 298, GLU A: 185, PHE A: 137, PHE A: 222, HIS A: 246, HIS A: 128, PHE A: 251, TRP A: 356
Naamidine A	HIS A: 128, LEU A: 297, ASP A: 138, GLU A: 255, GLY A: 136, TRP A: 356, PHE A: 222, PHE A: 251, ASN A: 298
Naamidine B	GLU A: 185, ASN A: 298, ARG A: 302, TRP A: 356, TRP A: 366, TYR A: 248, PHE A: 251, PHE A: 222, PHE A: 137
Naamidine C	TYR A: 22, SER A: 285, TYR A: 248, ARG A: 305, GLU A: 20, HIS A: 128, TRP A: 356, HIS A: 247, PHE A: 137, GLU A: 185, PHE A: 251
Hyrtimomine A	GLU A: 185, SER A: 285, TRP A: 356, PHE A: 251, PHE A: 137
Hyrtimomine B	TYR A: 248, GLU A: 185, TRP A: 356, PHE A: 251, SER A: 285
Hyrtimomine C	ASN A: 139, TRP A: 356, TYR A: 248, GLU A: 20, PHE A: 251, PHE A: 137, ASP A: 138, GLU A: 185
Hyrtimomine F	ASN A: 139, LEU A: 297, GLY A: 136, ARG A: 305, ARG A: 302, PHE A: 137, ARG A: 143
Hyrtimomine G	GLU A: 185, TYR A: 22, GLU A: 20, ASN A: 139, ASP A: 138, GLY A: 136, TRP A: 356, PHE A: 137, ARG A: 302, PHE A: 251
Topsentin	LEU A: 297, ASP A: 138, ARG A: 305, GLU A: 185, GLY A: 136, PHE A: 137, PHE A: 251, ARG A: 302
Topsentin A	ASP A: 138, LEU A: 297, GLU A: 185, GLY A: 136, PHE A: 137, ARG A: 302, PHE A: 251
Topsentin D	LEU A: 297, GLU A: 185, GLY A: 136, PHE A: 137, ARG A: 302, PHE A: 251
Latrunculin A	PHE A: 251, PHE A: 137
Latrunculin B	TYR A: 310, PHE A: 251, PHE A: 137, ASN A: 298
Latrunculin S	ARG A: 258, PHE A: 222, TRP A: 270
Xestodecalactone A	ASN A: 139, ARG A: 305, TRP A: 356, TYR A: 22, PHE A: 251
Xestodecalactone B	ASN A: 139, LEU A: 297, PHE A: 137
Xestodecalactone C	ASN A: 139, PHE A: 137, PHE A: 251, LEU A: 297
Xestodecalactone D	ASN A: 139, LEU A: 297, ARG A: 305, ASN A: 298, PHE A: 251, PHE A: 137, TYR A: 310
Xestodecalactone E	ASP A: 144, ASP A: 138, ASN A: 135, TYR A: 146, PHE A: 137, PHE A: 251, ARG A: 302
Xestodecalactone F	ASP A: 138, ASN A: 139, ASN A: 298, TYR A: 248, PHE A: 137, TRP A: 356, ARG A: 143
(+)-Curcudiol	LEU A: 297, PHE A: 222, PHE A: 137, PHE A: 251
(+)-Curcuphenol	GLU A: 185, PHE A: 137, PHE A: 251, TRP A: 356, TYR A: 248
Tetillapyrone	ASN A: 139, TRP A: 356, PHE A: 137, PHE A: 251
Nortetillapyrone	TYR A: 248, ASN A: 139, LEU A: 297, TRP A: 356
Aurantoside I	GLN A:223, HIS A: 247, GLU A: 255, TYR A: 248, ARG A: 258, PHE A: 251, ASN A: 269, HIS A: 246, TRP A: 356, HIS A: 128, PHE A: 222
Aurantoside K	THR A: 248, HIS A: 246, ASP A: 220, HIS A: 247, SER A: 252, PHE A: 251, PHE A: 337
**Drugs**	
Amphotericin B	HIS A: 246, PHE A: 225, ASP A: 273, ARG A: 258
Isavuconazole	ASN A: 139, HIS A: 246, PHE A: 222, TRP A: 270, PHE A: 137, PHE A: 251, ASP A: 220, PHE A: 225, HIS A: 247
Posaconazole	GLN A: 223, TYR A: 310, VAL A: 224, PHE A: 222, PHE A: 225, HIS A: 247, HIS A: 245, PHE A: 137, PHE A: 251

Table S9: Molecular docking output for the target RdRp

RdRp					
**Ligands**	**Binding affinity (Kcal/mol-1)**	**H bond residues**	**H bonds**	**C-H bonds**	**Hydrophobic bonds**
Naamine A	-7.3	TRP A: 134	1	1	4
Naamine B	-6.0	PHE A: 148, GLU A: 35	2	2	1
Naamine E	-6.3	GLU A: 35, ALA A: 38	2	1	2
Naamine F	-7.5	TRP A: 134	1	0	6
Naamine G	-6.9	None	0	2	4
Naamidine A	-7.6	TYR A: 13, PRO A: 10	2	0	3
Naamidine B	-7.6	ARG A: 49, SER A: 40, PHE A: 48	3	1	3
Naamidine C	-7.4	None	0	3	3
Hyrtimomine A	-8.1	None	0	2	2
Hyrtimomine B	-8.5	PRO A: 10, ILE A: 121	2	0	3
Hyrtimomine C	-8.1	ASP A: 89	1	2	2
Hyrtimomine F	-8.3	SER A: 151, LEU A: 41, PHE A: 48	3	2	2
Topsentin	-8.6	ARG A: 136	1	0	4
Topsentin A	-8.6	ILE A:121	1	0	5
Topsentin D	-8.5	ARG A: 162	1	2	2
Latrunculin A	-8.6	TRP A: 134	1	0	1
Latrunculin B	-7.7	None	0	0	3
Latrunculin S	-7.7	GLN A: 141	1	1	1
Xestodecalactone A	-7.3	ARG A: 49, SER A: 40, LEU A: 41	3	0	1
Xestodecalactone B	-7.2	PRO A: 39, LEU A: 41, SER A: 40, ARG A: 49	4	0	1
Xestodecalactone C	-6.8	PRO A: 10	1	0	3
Xestodecalactone D	-6.5	SER A: 151, SER A: 40	2	1	3
Xestodecalactone E	-6.7	ASP A: 89, GLY A: 138	2	0	4
Xestodecalactone F	-6.3	SER A: 151	1	0	3
Aurantoside K	-8.3	ILE A: 121, PHE A: 133, ARG A: 136, TYR A: 13	4	0	5
Aurantoside I	-8.3	VAL A: 139, ARG A: 136, GLY A: 138, LYS A: 3	4	1	5

Table S10: List of all interacting amino acids for RNA dependent/directed RNA polymerase

RdRp	
**Ligands**	**Interacting residues**
Naamine A	TRP A: 134, ASP A: 89, PRO A: 135, PRO A: 15, TYR A: 13, VAL A: 123
Naamine B	PHE A: 148, GLU A: 35, PRO A: 39, HIS A: 50, LEU A: 41
Naamine D	PRO A: 39, SER A: 40, LEU A: 41, ALA A: 38, ARG A: 49
Naamine E	GLU A: 35, ALA A: 38, GLU A: 35, PHE A: 148, ARG A: 49
Naamine F	TRP A: 134, PRO A: 135, ILE A: 121, VAL A: 139, PRO A: 10, TYR A: 13, VAL A: 123
Naamine G	PRO A: 15, VAL A: 125, VAL A: 123, PRO A: 10, ILE A: 121, LEU A: 8
Naamidine A	TYR A: 13, PRO A: 10, PHE A: 133, VAL A: 123, PRO A: 15
Naamidine B	ARG A: 49, SER A: 40, PHE A: 48, LEU A: 41, ILE A: 152, ALA A: 38, PHE A: 148
Naamidine C	VAL A: 123, PHE A: 133, LEU A: 8, PRO A: 10, VAL A: 125, PRO A: 15
Hyrtimomine A	TYR A: 13, PRO A: 10, VAL A: 123, PRO A: 15
Hyrtimomine B	PRO A: 10, ILE A: 121, ASP A: 89, PRO A: 15, PRO A: 135
Hyrtimomine C	ASP A: 89, ILE A: 121, PRO A: 15, PRO A: 135
Hyrtimomine F	SER A: 151, LEU A: 41, PHE A: 48, PHE A: 148, ARG A: 49, GLU A: 35, SER A: 40
Hyrtimomine G	GLU A: 35, SERA : 151, LEU A: 41, SER A: 40, ARG A: 49
Topsentin	ARG A: 136, PRO A: 10, VAL A: 123, PRO A: 15, PRO A: 135
Topsentin A	ILE A:121, VAL A: 125, PRO A: 10, VAL A: 123, PRO A: 15, PRO A: 135
Topsentin D	ARG A: 162, TRP A: 185, ARG A: 165, LEU A: 166, VAL A: 164
Latrunculin A	TRP A: 134, PRO A: 15
Latrunculin B	PHE A: 133, VAL A: 123, TYR A: 13
Latrunculin S	GLN A: 141, PHE A: 133, LEU A: 140
Xestodecalactone A	ARG A: 49, SER A: 40, LEU A: 41, SER A: 151
Xestodecalactone B	PRO A: 39, LEU A: 41, SER A: 40, ARG A: 49, SER A: 151
Xestodecalactone C	PRO A: 10, VAL A: 123, ILE A: 121, PRO A: 135
Xestodecalactone D	SER A: 151, SER A: 40, PHE A: 148, ALA A: 38, ILE A: 152, LEU A: 41
Xestodecalactone E	ASP A: 89, GLY A: 138, VAL A: 123, PRO A: 10, TYR A: 13, PRO A: 15
Xestodecalactone F	SER A: 151, LEU A: 41, PHE A: 148, PRO A:39
(+)-Curcudiol	PHE A: 133, VAL A: 123,PRO A: 10, VAL A: 125
(+)-Curcuphenol	TYR A: 2, VAL A: 125, PRO A: 10, TYR A: 13, VAL A: 123, PHE A: 133, PRO A: 15, ILE A: 121
Tetillapyrone	ARG A: 165, TRP A: 185, LEU A: 166, VAL A: 164
Nortetillapyrone	HIS A: 50, SER A: 151
Aurantoside K	ILE A: 121, PHE A: 133, ARG A: 136, TYR A: 13, VAL A: 123, PRO A: 10, VAL A: 125, PHE A: 128, GLY A: 138
Aurantoside I	VAL A: 139, ARG A: 136, GLY A: 138, LYS A: 3, ASP A: 89, VAL A: 125, VAL A: 123, PRO A: 10, TYR A: 13, SER A: 86
**Drugs**	
Amphotericin B	ARG A: 136, ASP A: 89, TRP A: 134, PHE A: 128
Isavuconazole	PRO A: 10, VAL A: 123, PRO A: 15, PRO A: 135, ILE A: 121
Posaconazole	TRP A: 134, VAL A: 123, PRO A: 10, PHE A: 128, PRO A: 15, PRO A: 135, ILE A: 121

Table S11: Molecular docking output for the target Rhizopuspepsin

Rhizopuspepsin					
**Ligands**	**Binding affinity (Kcal/mol-1)**	**H bond residues**	**H bonds**	**C-H bonds**	**Hydrophobic bonds**
Naamine A	-7.6	ASP A: 218	1	0	2
Naamine B	-7.3	ASP A: 79	1	2	3
Naamine E	-7.0	ASP A: 218, ASP A: 79	2	0	2
Naamine F	-7.1	SER A: 76, GLY A: 220, THR A: 221	3	0	4
Naamine G	-6.1	ASP A: 276, VAL A: 277	2	0	3
Naamidine A	-8.5	ASP A: 35, GLY A: 78, ASP A: 79, SER A: 113	4	0	3
Naamidine B	-7.8	ASP A: 218, TRP A: 294, ILE A: 130, SER A: 76	4	2	3
Naamidine C	-7.8	THR A: 221, ARG A: 192, GLY A: 37	3	0	3
Hyrtimomine A	-9.1	GLU A: 16	1	0	6
Hyrtimomine B	-9.3	ASP A: 218, ASP A: 35, THR A: 222, GLU A: 16	4	0	3
Hyrtimomine C	-7.9	GLY A: 37, SER A: 76	2	0	4
Hyrtimomine F	-9.0	GLY A: 37, GLY A: 78	2	2	3
Topsentin	-9.3	ASP A: 79	1	2	3
Topsentin A	-9.5	ASP A: 35, GLY A: 37, GLU A: 16, ASP A: 79, GLY A: 78	5	2	3
Topsentin D	-9.6	GLY A: 220, GLU A: 16, ASP A: 33, SER A: 81	4	0	2
Latrunculin A	-8.3	ASP A: 79	1	0	0
Latrunculin B	-7.9	None	0	0	0
Xestodecalactone A	-6.7	PHE A: 278	1	0	1
Xestodecalactone B	-6.7	ASP A: 274, ASN A: 13, LYS A: 305	3	0	0
Xestodecalactone C	-7.7	ASN A: 13, ASP A: 14, SER A: 275	3	0	2
Xestodecalactone D	-6.9	None	0	1	1
Xestodecalactone E	-7.0	THR A: 222, GLY A: 220	2	0	2
Xestodecalactone F	-7.7	None	0	0	7
(+)-Curcudiol	-6.8	ASP A: 79	1	0	5
Tetillapyrone	-6.4	ASP A: 79, ASP A: 35	2	0	4
Nortetillapyrone	-6.5	ASP A: 79, ASP A: 33	2	0	1
Aurantoside I	-8.8	GLY A: 78, GLY A: 37, ASP A: 33	3	1	7
Aurantoside K	-8.1	GLU A: 16, GLY A: 220	2	2	5

Table S12: List of all interacting amino acids for rhizopuspepsin

	Rhizopuspepsin
**Ligands**	**Interacting residues**
Naamine A	ASP A: 218, TYR A: 77, SER A: 39
Naamine B	ASP A: 79, ILE A; 75, TRP A: 194, ASP A: 218, GLY A: 220, GLY A: 78
Naamine D	ASN A: 13, VAL A: 277, ALA A: 161, LYS A: 162, ASP A: 14, GLU A: 280
Naamine E	ASP A: 218, ASP A: 79, ILE A: 298, TYR A: 77
Naamine F	SER A: 76, GLY A: 220, THR A: 221, ILE A: 130, TYR A: 77, ASP A: 218, ILE A: 298
Naamine G	ASP A: 276, VAL A: 277, LYS A: 162, ASP A: 14, LYS A: 305
Naamidine A	ASP A: 35, GLY A: 78, ASP A: 79, SER A: 113, TYR A: 77, LEU A: 223, ILE A: 225
Naamidine B	ASP A: 218, TRP A: 294, ILE A: 130, SER A: 76, GLY A: 220, GLY A: 78, PHE A: 296, TRP A: 194, THR A: 132
Naamidine C	THR A: 221, ARG A: 192, GLY A: 37, ASP A: 218, TRP A: 194, ILE A: 130
Hyrtimomine A	GLU A: 16, ASP A: 33, LEU A: 122, TYR A: 77, ASP A: 79, ASP A: 218, ILE A: 298
Hyrtimomine B	ASP A: 218, ASP A: 35, THR A: 222, GLU A: 16, TYR A: 77, ASP A: 33, LEU A: 122
Hyrtimomine C	GLY A: 37, SER A: 76, ASPA: 218, ASP A: 35, TRP A: 194, ILE A: 130
Hyrtimomine F	GLY A: 37, GLY A: 78, ILE A: 130, TRP A: 194, ASP A: 218
Hyrtimomine G	ASP A: 33, THR A: 222, ILE A: 15, ASP A: 79, ASP A: 35, ASP A: 218, GLU A: 16
Topsentin	ASP A: 79, LEU A: 122, TRP A: 194, ILE A: 130, ASP A: 218, GLY A: 78
Topsentin A	ASP A: 35, GLY A: 37, GLU A: 16, ASP A: 79, GLY A: 78, PHE A: 114, ASP A: 218, ILE A: 298, ASP A: 33, GLY A: 220
Topsentin D	GLY A: 220, GLU A: 16, ASP A: 33, SER A: 81, ASP A: 218, ASP A: 79
Latrunculin A	ASP A: 79
Latrunculin B	No residue interaction (Van der Waals only)
Latrunculin S	ASP A: 79, GLY A: 220, THR A: 222
Xestodecalactone A	PHE A: 278, ASN A: 13
Xestodecalactone B	ASP A: 274, ASN A: 13, LYS A: 305
Xestodecalactone C	ASN A: 13, ASP A: 14, SER A: 275, LYS A: 162, VAL A: 277
Xestodecalactone D	SER A: 38, TRP A: 194
Xestodecalactone E	THR A: 222, GLY A: 220, LEU A: 223, ASP A: 79
Xestodecalactone F	ILE A: 298, TRP A: 194, TRP A: 294, PHE A: 296, ASP A: 79, THR A: 221, THR A: 222
(+)-Curcudiol	ASP A: 79, PHE A: 296, TRP A: 294, ILE A: 298, TRP A: 194, TYR A: 77
(+)-Curcuphenol	ASP A: 218, ASP A: 35, LEU A: 122, ILE A: 216, ILE A: 298, PHE A: 296, TRP A: 194
Tetillapyrone	ASP A: 79, ASP A: 35, TYR A: 77, LEU A: 122, ASP A: 33, GLU A: 16
Nortetillapyrone	ASP A: 79, ASP A: 33, ASP A: 218
Aurantoside K	GLU A: 16, GLY A: 220, TRP A: 294, TRP A: 194, PHE A: 296, ILE A: 216, ILE A: 298, ASP A: 79, SER A: 113
Aurantoside I	GLY A: 78, GLY A: 37, ASP A: 33, TYR A: 77, ASP A: 79, GLU A: 16, PHE A: 278, ILE A: 286, PHE A: 281, LEU A: 223, GLY A: 220
**Drugs**	
Amphotericin B	GLY A: 220, GLU A: 279, GLY A: 246
Isavuconazole	SER A: 81, SER A: 113, LEU A: 122, ASP A: 33, GLU A: 16
Posaconazole	ARG A: 192, GLY A: 78, GLY A: 37, GLY A: 220, ASP A: 33, ASP A: 79

Table S13: Molecular docking output for the target lanosterol 14 alpha-demethylase

		**Lanosterol 14 alpha-demethylase**			
**Ligands**	**Binding affinity (Kcal/mol-1)**	**H bond residues**	**H bonds**	**C-H bonds**	**Hydrophobic bonds**
Naamine A	-8.7	HIS A: 297, PHE A: 293, HIS A: 453	3	1	8
Naamine B	-8.1	PHE A: 293, HIS A: 297	2	3	9
Naamine D	-6.9	GLN A: 459	1	0	4
Naamine E	-6.9	LYS A: 466	1	0	5
Naamine F	-7.2	SER A: 147	1	1	5
Naamine G	-6.8	None	0	0	6
Naamidine A	-7.8	GLN A: 459, LYS A: 341	2	2	3
Naamidine B	-7.8	LYS A: 341	1	2	3
Naamidine C	-8.1	LYS A: 341, GLN A: 459	2	3	4
Hyrtimomine A	-8.6	None	0	2	6
Hyrtimomine B	-8.7	ASN A: 429, ALA A: 436, LYS A: 142, ILE A: 456	4	0	1
Hyrtimomine C	-9.2	THR A: 492, SER A: 493	2	0	3
Hyrtimomine F	-9.1	ASN A: 146, LYS A: 142, ASN A: 429, ILE A: 456	4	1	3
Hyrtimomine G	-8.9	HIS A: 453	1	3	9
Topsentin	-9.0	ASP A: 417, THR A: 344, LYS A: 466	3	0	4
Topsentin A	-9.2	LYS A: 466, ASP A: 417, THR A: 344	3	0	5
Topsentin D	-8.9	THR A: 344, ASP A: 417, SER A: 147, LYS A: 341	4	0	2
Latrunculin A	-8.4	ALA A: 436	1	1	0
Latrunculin B	-8.7	TYR A: 178, ASP A: 214, GLN A: 215	3	0	4
Latrunculin S	-8.9	ASP A: 214	1	1	0
Xestodecalactone A	-8.4	GLN A: 459, PHE A: 337	2	0	2
Xestodecalactone B	-7.5	TYR A: 127	1	0	5
Xestodecalactone C	-7.7	GLN A: 459, LEU A: 463	2	0	3
Xestodecalactone D	-7.0	ALA A: 290, TRY A: 127	2	1	5
Xestodecalactone E	-8.9	ARG A: 365, ALA A: 290	2	0	8
Xestodecalactone F	-7.0	ASP A: 347	1	4	3
Tetillapyrone	-7.5	TYR A: 113, HIS A: 453	2	0	2
Nortetillapyrone	-6.8	ARG A: 365, TYR A: 127	2	0	3
Aurantoside I	-8.4	ASP A: 417, LYS A: 341, ASP A: 347, LYS A: 466	4	3	4
Aurantoside K	-8.3	LYS A: 327, GLU A: 342, GLU A: 323, ASP A: 319	4	1	6

Table S14: List of all interacting amino acids for Lanosterol 14 alpha-demethylase

Lanosterol 14 alpha-demethylase	
**Ligands**	**Interacting residues**
Naamine A	HIS A: 297, PHE A: 293, HIS A: 453, TYR A: 113, ILE A: 360, ARG A: 365, CYS A: 455, ALA A: 290, VAL A: 291, ILE A: 456, ILE A: 141, TYR A: 127
Naamine B	PHE A: 293, HIS A: 297, PHE A: 217, TYR A: 113, ARG A: 365, ILE A: 360, MET A: 363, ILE A: 141, VAL A: 291, ALA A: 290, ILE A: 456, CYS : 455, PHE A: 448, TYR A: 127
Naamine D	GLN A: 459, HIS A: 420, LEU A: 463, LYS A: 341, VAL A: 419
Naamine E	LEU A: 463, PHE A: 337, VAL A: 419, PRO A: 418, ASP A: 417, LYS A: 466
Naamine F	VAL A: 419, LEU A: 463, LEU A: 340, LYS A: 341, ASP A: 417 SER A: 147, PHE A: 337
Naamine G	LYS A: 218, VAL A: 497, ASP A: 214, HIS A: 179, HIS A: 297, TYR A: 178
Naamidine A	GLN A: 459, LYS A: 341, ASP A: 417, ASP A: 347, VAL A: 419, LYS A: 466, LYS A: 341
Naamidine B	LYS A: 341, ASP A: 417, ASP A: 347, VAL A: 419, LYS A: 466, HIS A: 420
Naamidine C	LYS A: 341, GLN A: 459, SER A: 147, ASP A:347, ASP A: 417, LYS A: 466, VAL A: 419, PRO A: 418, LYS A: 341
Hyrtimomine A	TYR A: 127, ARG A: 454, CYS A: 455, ILE A: 456, LYS A: 138, VAL A: 126, ALA A: 290, MET A: 494
Hyrtimomine B	ASN A: 429, ALA A: 436, LYS A: 142, ILE A: 456, ARG A: 454
Hyrtimomine C	THR A: 492, SER A: 493, ASP A: 214, VAL A: 497, LYS A: 218
Hyrtimomine F	ASN A: 146, LYS A: 142, ASN A: 429, ILE A: 456, ALA A: 436, PHE A: 434, ARG A: 454, ALA A: 424
Hyrtimomine G	HIS A: 453, LYS A: 138, VAL A: 126, MET A: 494, ILE A: 360, TYR A: 113, ARG A: 365, CYS A: 455, MET A: 363, ILE A: 456, TYR A: 127, ALA A: 290, GLY A: 294
Topsentin	ASP A: 417, THR A: 344, LYS A: 466, PRO A: 418, VAL A: 419, PHE A: 337, LEU A: 463
Topsentin A	LYS A: 466, ASP A: 417, THR A: 344, LEU A: 463, PHE A: 337, VAL A: 419, ASP A: 347, PRO A: 418
Topsentin D	THR A: 344, ASP A: 417, SER A: 147, LYS A: 341, LEUA: 463, PRO A: 418
Latrunculin A	ALA A: 436, HIS A: 420
Latrunculin B	TYR A: 178, ASP A: 214, GLN A: 215, HIS A: 297, VAL A: 497, HIS A: 179, TYR A: 211
Latrunculin S	ASP A: 214, GLN A: 215
Xestodecalactone A	GLN A: 459, PHE A: 337, LYS A: 341, LEU A: 463
Xestodecalactone B	TYR A: 127, ILE A: 360, CYS A: 455, ARG A: 365, PHE A: 217, HIS A: 297
Xestodecalactone C	GLN A: 459, LEU A: 463, LYS A: 341, PHE A: 337, LYS A: 466
Xestodecalactone D	ALA A: 290, TYR A: 127, ILE A: 360, HIS A: 297, VAL A: 495, PHE A: 217,MET A: 494, GLY A: 294
Xestodecalactone E	ARG A: 365, ALA A: 290, TYR A: 127, ILE A: 141, VAL A: 291, HIS A: 297, MET A: 494, PHE A: 217, CYS A: 455, ILE A: 456
Xestodecalactone F	ASP A: 347, ASP A: 417, PHE A: 337, LEU A: 463, PRO A: 418, THR A: 344, LYS A: 341, SER A: 147
(+)-Curcudiol	VAL A: 495, MET A: 494, HIS A: 297, PHE A: 217, TYR A: 127, VAL A: 126, ALA A: 290, PHE A: 121
(+)-Curcuphenol	LYS A: 466, LYS A: 341, LEU A: 463, PHE A: 337
Tetillapyrone	TYR A: 113, HIS A: 453, PHE A: 217, MET A: 494
Nortetillapyrone	ARG A: 365, TYR A: 127, TYR A: 113, HIS A: 453, PHE A: 217
Aurantoside I	ASP A: 417, LYS A: 341, ASP A: 347, LYS A: 466, PRO A: 418, THR A: 416, LYS A: 341, LEU A: 345, ILE A: 413, LEU A: 463, PHE A: 337
Aurantoside K	LYS A: 327, GLU A: 342, GLU A: 323, ASP A: 319, LEU A: 463, VAL A: 419, PRO A: 418, TYR A: 462, LYS A: 341, PHE A: 337, ASN A: 414
**Drugs**	
Amphotericin B	ASP A: 176, ASN A: 503, PRO A: 501, LYS A: 484, ARG A: 500, TYR A: 168
Isavuconazole	LEU A: 340, PHE A: 337, LYS A: 341, LEU A: 463, GLN A: 459, ASP A: 417, PRO A: 418, VAL A: 419
Posaconazole	LYS A: 466, SER A: 147, ILE A: 413, LEU A: 345, ASP A: 417, LYS A: 341, TYR A: 462, VAL A: 419, THR A: 344

Table S15: Molecular docking output for the target lipase

Fungal Lipase					
**Ligands**	**Binding affinity (Kcal/mol-1)**	**H bond residues**	**H bonds**	**C-H bonds**	**Hydrophobic bonds**
Naamine A	-7.5	None	0	0	3
Naamine B	-7.5	None	0	1	4
Naamine D	-6.1	ASP A: 217, ILE A: 221	2	1	5
Naamine E	-6.1	ASN A: 265, SOA : 4401, VAL A: 216, PHE A: 223	4	1	2
Naamine F	-7.1	SER A: 115	1	2	3
Naamine G	-6.2	SER A: 142	1	0	4
Naamidine A	-7.0	VAL A: 121	1	0	3
Naamidine B	-7.9	SER A: 115, ASP A: 119, THR A: 88, THR A: 110	4	1	2
Naamidine C	-7.9	SER A: 198, HIS A: 224, PHE A: 223, SER A: 250	4	1	3
Hyrtimomine A	-7.6	THR A: 88, SER A: 142	2	0	2
Hyrtimomine B	-7.8	GLN A: 187, ASN A:144	2	0	3
Hyrtimomine C	-6.9	GLN A: 187	1	0	4
Hyrtimomine F	-7.2	ASP A: 184	1	1	2
Hyrtimomine G	-7.7	GLN A: 187, ASP A: 184, LEU A: 180, ASN A: 144	4	0	2
Topsentin	-7.6	SER A: 128, ASN A: 144, GLN A: 187, ASP A: 184	4	0	4
Topsentin A	-7.5	ARG A: 188, GLN A: 187, ASN A: 144,	3	0	4
Topsentin D	-7.6	ASP A: 217, THR A: 263	2	1	3
Latrunculin A	-7.5	VAL A: 121, HIS A: 136, THR A: 88	3	0	1
Latrunculin B	-6.9	VAL A: 121	1	1	1
Latrunculin S	-7.3	ASP A: 119	1	0	0
Xestodecalactone A	-7.2	None	0	0	2
Xestodecalactone B	-6.4	None	0	0	5
Xestodecalactone C	-6.2	THR A: 45	1	1	1
Xestodecalactone D	-6.2	SER A: 198, ASP A: 119	2	1	1
Xestodecalactone E	-6.9	THR A: 88, THR A: 110	2	0	5
Xestodecalactone F	-6.1	HIS A: 136, VAL A: 121	2	0	2
Tetillapyrone	-5.9	PHE A: 223, ASP A: 217	2	0	2
Nortetillapyrone	-6.2	ASN A: 265, ASP A: 217, VAL A: 216, PHE A: 223	4	0	1
Aurantoside I	-7.4	SER A: 128, ARG A: 188, LYS A: 148	3	1	3
Aurantoside K	-7.0	ARG A: 188, ASP A: 184, GLN A: 187, ASN A: 144	4	0	2

Table S16: List of all interacting amino acids for fungal lipase

Lipase	
**Ligands**	**Interacting residues**
Naamine A	VAL A: 121, ALA A: 116, HIS A: 136
Naamine B	ALA A: 116, THR A: 88, LEU A: 173, PHE A: 139, MET A: 120
Naamine D	ASP A: 217, ILE A: 221, VAL A: 248, PRO A: 222, ASN A: 265, GLY A: 247, PHE A: 223, THR A: 263
Naamine E	ASN A: 265, SOA : 4401, VAL A: 216, PHE A: 223, ILE A: 221, VAL A: 248, PRO A: 222
Naamine F	SER A: 115, LEU A: 173, SER A: 142, PHE A: 139, THR A: 88, ALA A: 116
Naamine G	SER A: 142, ALA A: 116, LEU A: 173, THR A: 88, PHE A: 139
Naamidine A	VAL A: 121, ALA A: 116, THR A: 88, THR A: 110
Naamidine B	SER A: 115, ASP A: 119, THR A: 88, THR A: 110, ALA A: 116, LEU A: 173, ALA A: 137
Naamidine C	SER A: 198, HIS A: 224, PHE A: 223, SER A: 250, ILE A: 261, VAL A: 243, PRO A: 222, TYR A: 200
Hyrtimomine A	THR A: 88, SER A: 142, LEU A: 173, VAL A: 121
Hyrtimomine B	GLN A: 187, ASN A:144, ASP A: 184, TYR A: 127, PRO A: 129
Hyrtimomine C	GLN A: 187, ARG A: 188, ASP A: 184, LEU A: 140, PRO A: 129
Hyrtimomine F	ASP A: 184, PRO A: 129, LEU A: 140
Hyrtimomine G	GLN A: 187, ASP A: 184, LEU A: 180, ASN A: 144, ARG A: 188, PRO A: 129
Topsentin	SER A: 128, ASN A: 144, GLN A: 187, ASP A: 184, VAL A: 147, LYS A: 148, TYR A: 148, TYR A: 127, PRO A: 129
Topsentin A	ARG A: 188, GLN A: 187, ASN A: 144, PRO A: 129, SER A: 128, TYR A: 127, ASP A: 184
Topsentin D	ASP A: 217, THR A: 263, ASN A: 265, VAL A: 248, ILE A: 261, SOA : 4401
Latrunculin A	VAL A: 121, HIS A: 136, THR A: 88, PHE A: 139
Latrunculin B	VAL A: 121, LEU A: 173, GLY A: 138
Latrunculin S	ASP A: 119
Xestodecalactone A	THR A: 88, LEU A: 173
Xestodecalactone B	ALA A: 116, VAL A: 121, LEU A: 173, PHE A: 139, ALA A: 116
Xestodecalactone C	THR A: 45, ILE A: 41, LYS A: 42
Xestodecalactone D	SER A: 115, ASP A: 119, ALA A: 116
Xestodecalactone E	THR A: 88, THR A: 110, PHE A: 139, LEU A: 173, HIS A: 136, VAL A: 121, MET A: 120
Xestodecalactone F	HIS A: 136, VAL A: 121, LEU A: 173, PHE A: 139
(+)-Curcudiol	ASN A: 144, LEU A: 140, TYR A: 127, PRO A: 129, TYR A: 143, LEU A: 180, ASP A: 184
(+)-Curcuphenol	ASN A: 144, ASP A: 184, TYR A: 143, LEU A: 180, TYR A: 127, PRO A: 129
Tetillapyrone	PHE A: 223, ASP A: 217, VAL A: 248, HIS A: 224
Nortetillapyrone	ASN A: 265, ASP A: 217, VAL A: 216, PHE A: 223, HIS A: 224
Aurantoside I	SER A: 128, ARG A: 188, LYS A: 148, PRO A: 129, ASN A: 144, TYR A: 186, TYR A: 215
Aurantoside K	ARG A: 188, ASP A: 184, GLN A: 187, ASN A: 144, LEU A: 140, ALA A: 137
**Drugs**	
Amphotericin B	GLN A: 100, ASN A: 46, PRO A: 162, ASN A: 196, LYS A: 42
Isavuconazole	ASP A: 217, VAL A: 216, PRO A: 194, PRO A: 222, VAL A: 248, LYS A: 195
Posaconazole	GLN A: 100, TYR A: 78, PRO A: 75, LYS A: 42, LEU A: 44, HIS A: 224, ASN A: 46, GLU A: 48, ALA A: 99, SER A: 250

Table S17: Lipinski and additional parameters to examine drug-likeness

Drug Like Physicochemical Properties						
**Ligands**	**Mol. Weight (g/mol)**	**Rotatable bonds**	**H bond donors**	**H bond acceptors**	**C Log P**	**TPSA**
	**MW ≤ 500**	**RB ≤ 10**	**HBD ≤ 5**	**HBA ≤ 10**	**Log p ≤ 5**	**(Å^2) ≤ 140**
Naamine A	323.39	5	2	3	2.7	73.30
Naamine B	367.44	6	2	4	2.91	72.4
Naamine E	369.41	6	3	5	2.39	102.76
Naamine F	353.41	6	2	4	2.7	82.53
Naamine G	383.44	7	2	5	2.7	91.76
Naamidine A	433.46	6	2	6	2.53	109.05
Naamidine B	463.49	7	2	7	2.39	118.28
Naamidine C	447.49	6	1	6	2.78	100.26
Hyrtimomine B	359.33	1	4	5	2.02	111.38
Hyrtimomine C	331.32	1	4	4	2.3	101.47
Hyrtimomine F	375.33	2	5	5	1.26	135.28
Hyrtimomine G	380.35	5	6	6	1.15	146.64
Topsentin	342.35	3	4	3	2.89	97.56
Topsentin A	326.35	3	3	2	3.24	77.33
Topsentin D	328.37	3	3	2	2.64	73.04
Latrunculin B	395.51	1	2	5	2.51	110.16
Xestodecalactone A	264.27	0	2	5	1.75	83.83
Xestodecalactone B	280.27	0	3	6	0.89	104.06
Xestodecalactone C	280.27	0	3	6	0.89	104.06
Xestodecalactone D	310.30	1	3	7	0.97	113.29
Xestodecalactone E	366.41	5	2	7	2.42	102.29
Xestodecalactone F	350.41	5	2	6	2.78	85.22
Tetillapyrone	242.23	2	3	6	0.11	100.13
Nortetillapyrone	228.20	2	3	6	-0.25	100.13
Aurantoside K	743.15	12	8	15	-1.11	268.23

Table S18: Swiss-ADME selective parameters

Swiss-ADME analysis				
**Ligands**	**Water solubility**	**Bioavailability**	**GI Absorption**	**BBB Permeant**
Naamine A	Moderate	0.55	High	Yes
Naamine B	Moderate	0.55	High	Yes
Naamine E	Moderate	0.55	High	No
Naamine F	Moderate	0.55	High	No
Naamine G	Poor	0.55	High	No
Naamidine A	Poor	0.55	High	No
Naamidine B	Poor	0.55	High	No
Naamidine C	Poor	0.55	High	No
Hyrtimomine A	Poor	0.55	High	No
Hyrtimomine B	Poor	0.56	High	No
Hyrtimomine C	Poor	0.55	High	No
Hyrtimomine F	Moderate	0.55	High	No
Hyrtimomine G	Moderate	0.55	Low	No
Topsentin	Poor	0.55	High	No
Topsentin A	Poor	0.55	High	No
Topsentin D	Poor	0.55	High	Yes
Latrunculin B	Soluble	0.55	High	No
Xestodecalactone A	Soluble	0.55	High	No
Xestodecalactone B	Soluble	0.55	High	No
Xestodecalactone C	Soluble	0.55	High	No
Xestodecalactone D	Soluble	0.55	High	No
Xestodecalactone E	Moderate	0.55	High	No
Xestodecalactone F	Soluble	0.55	High	No
Tetillapyrone	Soluble	0.55	High	No
Nortetillapyrone	Soluble	0.55	High	No
Aurantoside K	Soluble	0.11	Low	No

Table S19: OSIRIS analysis for potential harmful properties and druggability

OSIRIS							
**Ligands**	**Irritant potential**	**Mutagenic potential**	**Tumorigenic potential**	**Reproductive effectivity**	**Drug Score**	**Drug likeness**	
	**Risk Level**					**Score**	**Yes/No**
Naamine A	Low risk	Low risk	Low risk	Low risk	0.79	3.45	Yes
Naamine B	Low risk	Low risk	Low risk	Low risk	0.82	4.63	Yes
Naamine E	Low risk	Low risk	Low risk	Low risk	0.80	4.13	Yes
Naamine F	Low risk	Low risk	Low risk	Low risk	0.78	3.83	Yes
Naamine G	Low risk	Low risk	Low risk	Low risk	0.76	4.68	Yes
Naamidine A	Low risk	Low risk	Low risk	Low risk	0.74	4.83	Yes
Naamidine B	Low risk	Low risk	Low risk	Low risk	0.71	5.63	Yes
Naamidine C	Low risk	Low risk	Low risk	Low risk	0.74	4.13	Yes
Hyrtimomine B	Low risk	Low risk	Low risk	Low risk	0.65	0.51	Yes
Hyrtimomine C	Low risk	Low risk	Low risk	Low risk	0.58	0.48	Yes
Hyrtimomine F	Low risk	Low risk	High risk	Low risk	0.26	-2.19	No
Hyrtimomine G	Low risk	Low risk	Low risk	Low risk	0.53	-0.75	No
Topsentin	Low risk	Low risk	Low risk	Low risk	0.74	3.72	Yes
Topsentin A	Low risk	Low risk	Low risk	Low risk	0.70	3.2	Yes
Topsentin D	Low risk	Low risk	Low risk	Low risk	0.78	3.82	Yes
Latrunculin B	Low risk	Low risk	Low risk	Low risk	0.34	-10.38	No
Xestodecalactone A	Low risk	Low risk	Low risk	Low risk	0.45	-6.35	No
Xestodecalactone B	Low risk	Low risk	Low risk	Low risk	0.59	-0.93	No
Xestodecalactone C	Low risk	Low risk	Low risk	Low risk	0.59	-0.93	No
Xestodecalactone D	Low risk	Low risk	Low risk	Low risk	0.58	-1.01	No
Xestodecalactone E	High risk	Low risk	Low risk	Low risk	0.24	-6.96	No
Xestodecalactone F	High risk	Low risk	Low risk	Low risk	0.24	-6.99	No
Tetillapyrone	Low risk	Low risk	Low risk	Low risk	0.71	0.03	Yes
Nortetillapyrone	Low risk	Low risk	Low risk	Low risk	0.72	0.04	Yes
Aurantoside K	High Risk	Low risk	Low risk	High Risk	0.15	0.83	Yes

Table S20: Predictions for potential toxicity using ProTox-II and pkCSM servers

.	ProTox-II		PkCSM Toxicity Analysis		
**Ligands**	**LD50 value (mg/kg)**	**Toxicity Class**	**Hepatotoxicity**	***T.Pyriformis* (log ug/L)**	**Minnow (log mM)**
Naamine A	2000	4	Yes	0.285	0.448
Naamine B	3679	5	Yes	0.286	0.993
Naamine E	3740	5	No	0.285	1.149
Naamine F	2000	4	Yes	0.285	0.695
Naamine G	3740	5	No	0.285	1.04
Naamidine A	3200	5	Yes	0.285	3.248
Naamidine B	3250	5	Yes	0.285	3.593
Naamidine C	3200	5	Yes	0.285	3.136
Hyrtimomine B	2000	4	Yes	0.285	-0.04
Hyrtimomine C	2000	4	Yes	0.287	2.196
Hyrtimomine F	1500	4	Yes	0.286	2.083
Hyrtimomine G	200	3	No	0.292	3.537
Topsentin	1600	4	No	0.285	2.089
Topsentin A	1264	4	No	0.285	1.775
Topsentin D	750	4	Yes	0.299	0.219
Latrunculin B	1000	4	Yes	0.314	2.261
Xestodecalactone A	450	4	No	0.198	2.456
Xestodecalactone B	450	4	No	0.278	3.475
Xestodecalactone C	450	4	No	0.278	3.475
Xestodecalactone D	1500	4	No	0.306	3.789
Xestodecalactone E	1500	4	No	0.322	2.29
Xestodecalactone F	5000	5	No	0.382	1.843
Tetillapyrone	144	3	No	0.319	2.829
Nortetillapyrone	144	3	No	0.289	3.132
Aurantoside K	5000	5	No	0.285	9.573

Table S21: StopTox acute toxicity analysis:

StopTox Acute Toxicity Analysis					
**Ligands**	**Inhalation toxicity**	**Oral toxicity**	**Dermal toxicity**	**Skin sensitization**	**Irritation and corrosion**
Naamine A	No	Yes	No	Yes	Eyes (Yes), Skin (Yes)
Naamine B	No	Yes	No	Yes	Eyes (Yes), Skin (No)
Naamine E	No	Yes	No	Yes	Eyes (Yes), Skin (No)
Naamine F	No	Yes	No	Yes	Eyes (Yes), Skin (No)
Naamine G	No	Yes	No	Yes	Eyes (Yes), Skin (No)
Naamidine A	No	No	No	No	Eyes (Yes), Skin (No)
Naamidine B	No	No	No	No	Eyes (Yes), Skin (No)
Naamidine **C**	No	Yes	No	No	Eyes (Yes), Skin (No)
Hyrtimomine B	No	No	No	No	Eyes (Yes), Skin (No)
Hyrtimomine C	No	Yes	No	No	Eyes (Yes), Skin (No)
Hyrtimomine F	No	No	No	No	Eyes (Yes), Skin (No)
Hyrtimomine G	No	No	No	No	Eyes (Yes), Skin (No)
Topsentin	No	No	No	No	Eyes (Yes), Skin (No)
Topsentin A	No	Yes	No	No	Eyes (No), Skin (No)
Topsentin D	No	Yes	No	No	Eyes (No), Skin (No)
Latrunculin B	No	No	No	No	Eyes (Yes), Skin (No)
Xestodecalactone A	No	No	Yes	No	Eyes (Yes), Skin (No)
Xestodecalactone B	No	No	Yes	No	Eyes (Yes), Skin (No)
Xestodecalactone C	No	No	Yes	No	Eyes (Yes), Skin (No)
Xestodecalactone D	No	No	Yes	No	Eyes (No), Skin (No)
Xestodecalactone E	No	No	No	Yes	Eyes (No), Skin (No)
Xestodecalactone F	No	No	Yes	Yes	Eyes (No), Skin (No)
Tetillapyrone	No	No	No	No	Eyes (No), Skin (No)
Nortetillapyrone	No	No	No	No	Eyes (No), Skin (No)
Aurantoside K	No	No	No	No	Eyes (Yes), Skin (No)

Table S22: Molinspiration output for potential bioactivity

	Molinspiration Prediction					
**Ligands**	**GPCR Ligand**	**Ion channel Modulator**	**Kinase Inhibitor**	**Nuclear receptor Ligand**	**Protease Inhibitor**	**Enzyme Inhibitor**
Naamine A	0.36	0.10	0.20	-0.05	0.01	0.28
Naamine B	0.06	-0.35	-0.14	-0.23	-0.29	-0.06
Naamine D	0.29	0.13	0.34	-0.14	0.07	0.28
Naamine E	0.29	0.05	0.16	-0.13	-0.03	0.23
Naamine F	0.30	0.05	0.16	-0.11	-0.03	0.24
Naamine G	0.25	0.04	0.15	-0.12	-0.02	0.24
Naamidine A	0.24	-0.13	0.06	-0.27	-0.28	0.04
Naamidine B	0.20	-0.15	0.04	-0.30	-0.30	0.03
Naamidine C	0.24	-0.15	0.01	-0.32	-0.26	0.01
* Hyrtimomine A	0.17	0.09	0.53	-0.13	-0.23	0.30
Hyrtimomine B	0.32	0.09	0.18	0.05	-0.03	0.38
Hyrtimomine C	0.48	0.38	0.10	-0.03	0.01	0.29
Hyrtimomine F	0.19	0.17	-0.01	-0.29	-0.14	0.10
Hyrtimomine G	0.16	0.11	0.13	0.06	0.03	0.12
*Topsentin	0.50	0.16	0.70	0.17	-0.18	0.47
* Topsentin A	0.47	0.12	0.64	0.01	-0.20	0.42
Topsentin D	0.21	0.29	0.08	-0.22	-0.02	0.17
Latrunculin A	0.17	0.14	-0.53	0.55	0.06	0.51
Latrunculin B	0.15	0.11	-0.6	0.54	0.05	0.51
Latrunculin S	0.33	0.07	-0.54	0.50	0.28	0.47
Xestodecalactone A	-0.10	-0.11	-0.49	0.25	-0.19	0.26
Xestodecalactone B	0.12	0.02	-0.35	0.61	0.02	0.53
Xestodecalactone C	0.12	0.02	-0.35	0.61	0.02	0.53
Xestodecalactone D	0.12	-0.01	-0.30	0.40	-0.01	0.43
Xestodecalactone E	0.12	-0.05	-0.34	0.30	0.03	0.32
Xestodecalactone F	0.14	0.04	-0.20	0.34	-0.01	0.46
(+)-Curcudiol	-0.14	-0.01	-0.49	0.32	-0.38	0.01
(+)-Curcuphenol	-0.40	-0.1	-0.73	0.03	-0.67	-0.03
Tetillapyrone	-0.08	-0.66	-0.65	-0.65	0.03	0.59
Nortetillapyrone	-0.10	-0.65	-0.75	-0.78	-0.18	0.58
Aurantoside I	-0.85	-1.92	-1.59	-1.73	-0.49	-0.91
Aurantoside K	-0.70	-1.74	-1.40	-1.56	-0.37	-0.72
**Drugs**						
Amphotericin B	-3.06	-3.51	-3.54	-3.45	-2.45	-2.95
Isavuconazole	0	-0.07	0	-0.18	-0.07	0

Posaconazole	-0.63	-1.78	-1.40	-1.50	-0.57	-1.20

(* potential to be strong kinase inhibitors – effective against target CotH3)
